# Nuclear Outsourcing of RNA Interference Components to Human Mitochondria

**DOI:** 10.1371/journal.pone.0020746

**Published:** 2011-06-13

**Authors:** Simonetta Bandiera, Silvia Rüberg, Muriel Girard, Nicolas Cagnard, Sylvain Hanein, Dominique Chrétien, Arnold Munnich, Stanislas Lyonnet, Alexandra Henrion-Caude

**Affiliations:** 1 INSERM U781 Hôpital Necker – Enfants Malades, Paris, France; 2 Miltenyi Biotec GmbH, Bergisch Gladbach, Germany; 3 Paris-Descartes Bioinformatics Platform, Faculté de Médecine, Site Necker – Enfants Malades, Paris, France; French National Center for Scientific Research - Institut de biologie moléculaire et cellulaire, France

## Abstract

MicroRNAs (miRNAs) are small non-coding RNAs that associate with Argonaute proteins to regulate gene expression at the post-transcriptional level in the cytoplasm. However, recent studies have reported that some miRNAs localize to and function in other cellular compartments. Mitochondria harbour their own genetic system that may be a potential site for miRNA mediated post-transcriptional regulation. We aimed at investigating whether nuclear-encoded miRNAs can localize to and function in human mitochondria. To enable identification of mitochondrial-enriched miRNAs, we profiled the mitochondrial and cytosolic RNA fractions from the same HeLa cells by miRNA microarray analysis. Mitochondria were purified using a combination of cell fractionation and immunoisolation, and assessed for the lack of protein and RNA contaminants. We found 57 miRNAs differentially expressed in HeLa mitochondria and cytosol. Of these 57, a signature of 13 nuclear-encoded miRNAs was reproducibly enriched in mitochondrial RNA and validated by RT-PCR for hsa-miR-494, hsa-miR-1275 and hsa-miR-1974. The significance of their mitochondrial localization was investigated by characterizing their genomic context, cross-species conservation and instrinsic features such as their size and thermodynamic parameters. Interestingly, the specificities of mitochondrial versus cytosolic miRNAs were underlined by significantly different structural and thermodynamic parameters. Computational targeting analysis of most mitochondrial miRNAs revealed not only nuclear but also mitochondrial-encoded targets. The functional relevance of miRNAs in mitochondria was supported by the finding of Argonaute 2 localization to mitochondria revealed by immunoblotting and confocal microscopy, and further validated by the co-immunoprecipitation of the mitochondrial transcript *COX3*. This study provides the first comprehensive view of the localization of RNA interference components to the mitochondria. Our data outline the molecular bases for a novel layer of crosstalk between nucleus and mitochondria through a specific subset of human miRNAs that we termed ‘mitomiRs’.

## Introduction

Mitochondria are eukaryotic organelles that maintain and express their own genome, known as the mitochondrial DNA (mtDNA). The transcription and translation of the mtDNA as well as the processing of mitochondrial transcripts requires the involvement of several types of non-coding RNAs (ncRNA), which can be either mitochondrially encoded or transcribed within the nucleus and subsequently localized to mitochondria [1]. In human mitochondria, the full set of mitochondrial transfer RNAs (tRNAs) and two ribosomal RNAs (rRNAs), namely the 12S and 16S rRNAs, are transcribed from the mtDNA [2], while the RNA moiety of the RNase MRP enzymes [3], [4], [5], the 5S rRNA [6], [7], and two species of tRNA^Gln^ [8] are all RNAs delivered into mitochondria from the nucleus.

Among ncRNAs, microRNAs (miRNAs) have emerged as an important class of post-transcriptional regulators of gene expression in virtually all fundamental cellular processes [9]. MiRNAs are transcribed within the nucleus and are then extensively processed and matured in the cytosol as ∼22-bp double-stranded RNA. Mature miRNAs associate with Argonaute (AGO) proteins to form the core of a ribonucleoprotein complex named RNA-induced silcencing complex (RISC), which exerts RNA interference (RNAi) [10], [11]. RNAi occurs upon pairing one of the two miRNA strands, embedded in an AGO protein, with target sites in an mRNA, thereby affecting the stability/translation of this mRNA [11]. In mammals, there are four AGO proteins, AGO1 through AGO4, which were all shown to function in translational repression [12], but only AGO2 can catalyze the cleavage of the targeted transcript [13], [14]. Furthermore, knockdown and knockout of AGO2, respectively in human cells and in mice, suggest that the protein may have specific functions that may not be complemented by the other AGOs [13], [15].

Initially, mature miRNAs and AGO2 were believed to accumulate and function exclusively in the cytosol and/or into unstructured cytosolic foci, such as P-bodies and stress granules [16], [17]. However, mounting evidence suggests that they can also localize to and, possibly, function within different cellular compartments. So far, in human, miRNAs and AGO2 have been found to localize to the nucleus [18], [19], [20] and to multivesicular bodies [21], [22]. Recently, miRNAs were also identified in mitochondria purified from rat liver [23]. Interestingly, another possible link between mitochondria and RNAi came from the co-immunoprecipitation of human AGO2 with mitochondrial tRNA^Met^ [24].

In mitochondria, post-transcriptional regulation via miRNAs would provide a sensitive and rapid mechanism by which to adjust the expression of the mitochondrial genome in relation to the conditions and metabolic demands of the cell. Therefore, our aim was to investigate the possible link between miRNAs and mitochondria in human cells. Our study provides the first comprehensive view of the localization of RNAi components to human mitochondria.

## Results

### Investigating AGO2 at the mitochondria

**Figure 1:**
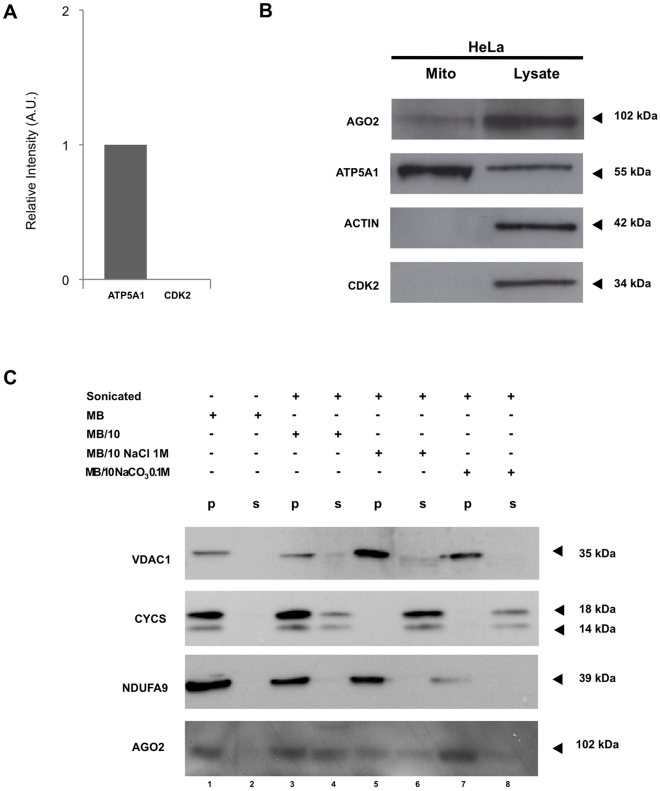
Novel localization of Argonaute 2 (AGO2) to human mitochondria. A. Purity assessment of mitochondrial fraction. Cyclin-dependent kinase 2 (CDK2) was assessed in the mitochondrial fraction by immunoblot analyses to check for nuclear and cytoplasmic contaminants relatively to mitochondrial protein ATP synthase subunit α (ATP5A1). The density of bands was measured using the ImageJ software and is represented as a relative intensity. Values are means±SD of three independent experiments. B. Western blot analysis of AGO2 in subcellular mitochondrial fraction from HeLa cells. AGO2 protein was detected using a rabbit polyclonal antibody anti-AGO2 in mitochondrial protein fraction (Mito) and total protein extracts (Lysate) from HeLa cells. ATP5A1 was used as a mitochondrial marker, actin as a cytosolic marker, and CDK2 as a nuclear/cytosolic marker. Representative image is shown of three independent experiments. C. Western blot analysis of AGO2 at mitochondrial membranous and soluble fraction from U2OS cells. Mitochondria were suspended either in isotonic mitochondrial buffer (MB) or in hypo-osmotic mitochondrial buffer (MB/10) alone or supplemented either with NaCl 1 M or Na_2_CO_3_ 0.1 M, and fragilized by freeze-thaw cycles and sonication when indicated. All samples were centrifuged (150,000× g) to separate the membrane pellet (p) from the soluble protein supernatants (s). 15 µg of protein extracts of each sample were subjected to Western Blot. The following proteins were immunodecorated on blots: voltage-dependent anion channel 1 (VDAC1) and NADH dehydrogenase (ubiquinone) 1 α subcomplex 9 (NDUFA9) as markers of mitochondrial membranes and cytochrome c (CYCS), as a intermembrane marker. Immunodetection of AGO2 is shown as the lower panel.

We first addressed the possibility that endogenous AGO2 could localize to mitochondria. To isolate highly-purified mitochondria, we performed cell fractionation combined with subsequent immunoisolation of mitochondria. This isolation procedure adapted from Hornig-Do et al. [25] was checked for efficiency by measuring the activity of mitochondrial and cytosolic marker enzymes (Supporting Information S1). Mitochondrial fraction was analyzed for its purity by immunoblot as assessed by the mitochondrial marker ATP synthase subunit α 1 (ATP5A1) and the nuclear/cytosolic marker cyclin-dependent kinase 2 (CDK2), which indicated the lack of nuclear and cytosolic contaminants reproducibly (Figure 1A). To study the localization of endogenous AGO2, we chose the same polyclonal antibody as that used in the founding studies of AGO2 localization [16], [26], [27]. By immunoblotting, we detected AGO2 as a band of ∼102 kDa in HeLa mitochondrial proteins as well as in the total protein extract from the same cells (Figure 1B). Assessment of actin as a cytosolic marker further validated the lack of cytosolic contaminants (Figure 1B). To further ascertain AGO2 at mitochondria, we extracted the crude membrane pellet and the soluble protein fraction from mitochondria isolated from a U2OS cell line. Mitochondria were incubated in hypo-osmotic buffer alone or supplemented with either 1 M NaCl or 0.1 M Na_2_CO_3_, pH 11 prior and after fragilization. Efficacy of treatments was ensured by immunoblotting detection of the following proteins: voltage-dependent anion channel 1 (VDAC1), cytochrome c (CYCS) and NADH dehydrogenase (ubiquinone) 1 α subcomplex 9 (NDUFA9), which all showed patterns consistent with the known location of those markers, respectively as a mitochondrial membrane marker, an intermembrane marker and a mitochondrial membrane marker (Figure 1C). As shown in Figure 1C, AGO2 was detected in the mitochondrial membrane fraction and to a lesser extent in the soluble fraction, suggesting its preferential association to mitochondrial membranes.

**Figure 2:**
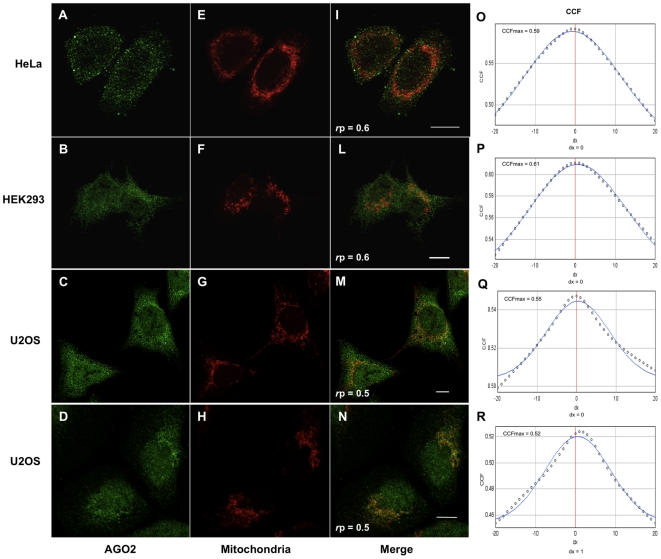
AGO2 protein co-localizes with mitochondria in human cell lines. Cells were fixed and endogenous AGO2 detected with two distinct antibodies (rabbit polyclonal anti-AGO2, clone 7C6, A, D; mouse-monoclonal anti-AGO2, clone 4G8, B, C) and visualized by indirect immunofluorescence. Mitochondria were either stained with Mitotracker Red CMXRos (E–G) or immunostained with anti-ATP5A1 (H). Overlay images show colocalization of AGO2 and mitochondria (I–N). A sample of 21 cells was examined for dual channel co-localization for each cell type. Co-localization was also confirmed by Pearson's correlation coefficient (*r*p), whose value is indicated at the bottom of each overlay image. Cross-correlation functions (CCFs) were calculated for each co-localization. Representative plots obtained from the analysis of a single microscopic field are shown (O–R). Maximum CCF and pixel shift (dx) values are respectively indicated at the top and the bottom of each plot. Scale bar, 10 µm.

Mitochondrial localization of AGO2 was further assessed by immunofluorescence confocal microscopy. Immunostaining of AGO2 showed a consistent punctuated cytoplasmic and nuclear pattern. Overlay of AGO2 immunostaining with mitochondrial staining indicated a partial co-localization as assessed by two distinct antibodies (Figure 2). Correlation between the intensities of green (AGO2) and red (mitochondria) in dual-channel images was studied using the Pearson's correlation coefficient (*r*p) in three different human cell lines, whether tumoral or transformed i.e HeLa, U2OS and HEK293 cells (Figure 2). Average values of *r*p≥0.5 indicated a significant correlation of green and red pixels, which was consistent in all cell types. Evidence of co-localization was improved using an additional parameter, which is the Van Steensel's cross-correlation function (CCF; [28]). Plotted CCF revealed curves with a bell-like shape further indicating that AGO2 and mitochondria were positively correlated (Figure 2).

Interestingly, the use of four prediction programs to identify subcellular protein localization (i.e TargetP [29], MitoProt II [30], Predotar [31] and ESLPred [32]) all consistently predicted a mitochondrial localization of AGO2, specifically when assessing the CRA-b isoform (Table S1). For that isoform, TargetP and MITOProt II delineated an N-terminal region of 9–24 amino acids that could support a mitochondrial targeting sequence (Table S1).

**Figure 3:**
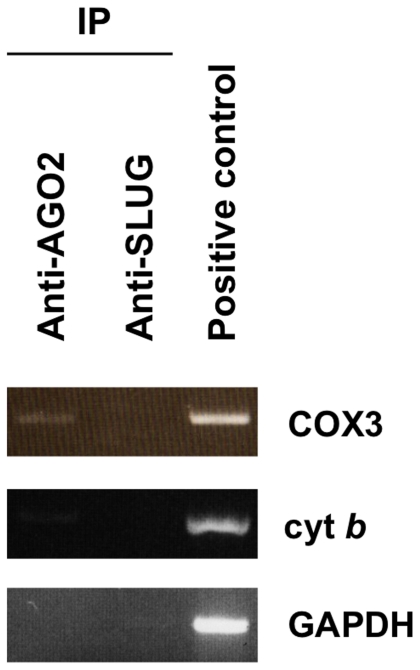
Co-immunoprecipitation of AGO2 and mitochondrial transcripts. Either AGO2, or SLUG, which serves as a negative control were co-immunoprecipitated with associated mRNAs in HeLa protein extracts. Coimmunoprecipitated RNA was extracted with Trizol and subjected to RT-PCR amplification with the indicated primers: cytochrome c oxidase III (*COX3*), cytochrome *b* (*cyt b*) and glyceraldehyde-3-phosphate dehydrogenase (*GAPDH*). Results are indicative of three independent experiments.

To assess the possibility of AGO2 to function at the mitochondria, we then examined whether AGO2 could interact with the mitochondrial transcript cytochrome c oxidase III (*COX3*) as previously found in HEK293 cells [33]. By co-immunoprecipitating endogenous AGO2 with the associated RNAs in HeLa cell extracts and subsequent RT-PCR, we consistently identified *COX3* as reproducibly associated, in comparison to a mitochondrial transcript cytochrome *b* (*cyt b*) and a cytosolic transcript glyceraldehyde-3-phosphate dehydrogenase (*GAPDH*) that were not associated (Figure 3). Those co-immunoprecipitation results were performed along the control immunoprecipitation of a transcriptional factor SLUG, which as expected did not lead to the identification of any targets. Collectively, our data support the novel localization and functioning of AGO2 at the human mitochondria, a finding that prompted us to search for mitochondrial miRNAs.

### Isolation of mitochondrial and cytosolic RNAs

**Figure 4:**
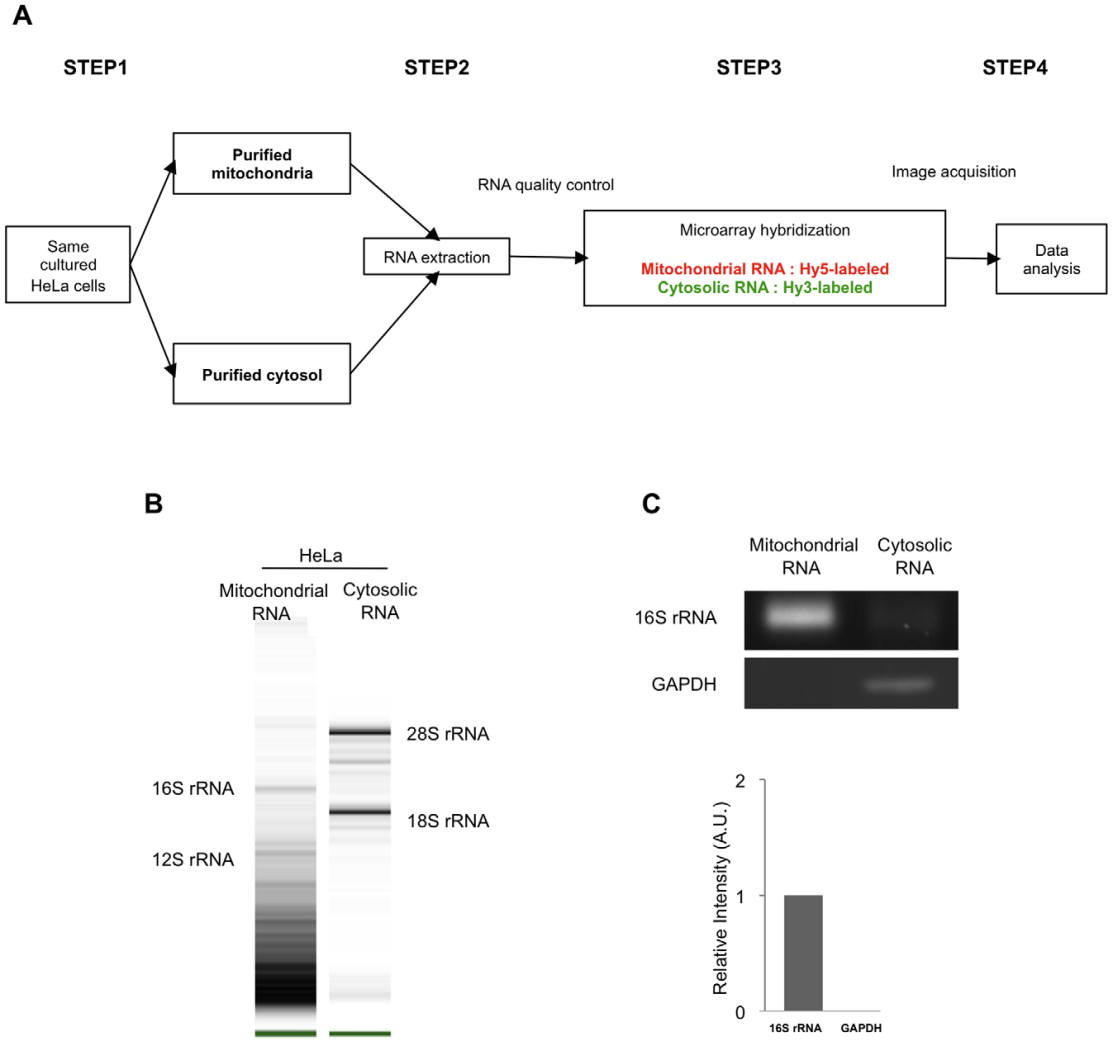
Isolation of mitochondrial and cytosolic miRNA. A. Schematic workflow of the experimental design. B. Integrity, quality and purity analyses of RNA fractions. Electrophoretic images of mitochondrial and cytosolic RNAs were retrieved from analysis using the Agilent 2100 Bioanalyzer. Bands corresponding to ribosomal RNAs (rRNAs) 16S, 12S, 28S and 18S are indicated when present. Representative image is shown of three independent experiments. C. Purity assessment of mitochondrial RNA fraction. 16S rRNA and *GAPDH* were amplified by RT-PCR in each fraction as shown by electrophoretic image. *GAPDH* was assessed in the mitochondrial fraction to check for cytoplasmic contaminant relatively to mitochondrial 16S ribosomal RNA. Representative image is shown of three independent experiments. The density of bands was measured using the ImageJ software and is represented as a relative intensity. Values are means±SD of three independent experiments.

We reasoned that differential identification of miRNAs in subcellular compartments from the same cells would provide the most reliable and effective method to investigate localization of miRNAs to mitochondria. Our experimental design enabled us to isolate mitochondria and cytosol fractions from the same cells and to profile differentially expressed miRNAs in each fraction using the miRXplore™ microarrays (Figure 4A). Total RNA was isolated respectively from mitochondrial and cytosolic fractions. Each RNA fraction was examined for its integrity, quality and purity through the Agilent 2100 Bioanalyzer. Electrophoretic gel images were observed for mitochondrial and cytosolic RNA fractions (Figure 4B). Consistently with the electrophoregrams, the 28S rRNA and 18S rRNA, which are located exclusively in the cytoplasm, were not observed in the mitochondrial RNA fraction indicating that mitochondrial and cytoplasmic RNA fractions were distinct. We further applied the RNA Integrity Number (RIN) algorithm to each sample to assign an integrity value [34]. Mean RIN values were respectively of 2.8 and 8.3 for the mitochondrial and cytosolic fractions. The cytosolic value was indicative of RNA of high quality. Since no standard exists as to the mitochondrial fraction, which consists of a distinct RNA population, we considered a value smaller than 6 as an indication of the depletion of cytosolic RNAs that we further ascertained by the depletion of cytosolic 18S and 28S rRNAs from mitochondrial RNA in the Bioanalyzer run. Finally, we assessed the purity of mitochondrial and cytosolic RNA fractions by reverse transcription-polymerase chain reaction (RT-PCR). 16S rRNA, which was chosen as a mitochondrial marker and thereby as a positive control for mitochondrial RNA, was enriched in the mitochondrial RNA fraction whereas it was depleted from the cytosolic RNA (Figure 4C). In contrast, *GAPDH*, an unambiguous cytosolic marker, could be amplified exclusively in the cytosolic fraction (Figure 4C). Altogether, these data indicated that no cytosolic contaminants could be detected in mitochondrial RNA, while faint signals of mitochondrial RNA were detected in the cytosol. These results are consistent with a high level of purity in either fraction.

### Differential expression of mature miRNAs in the mitochondria and the cytosol

**Figure 5:**
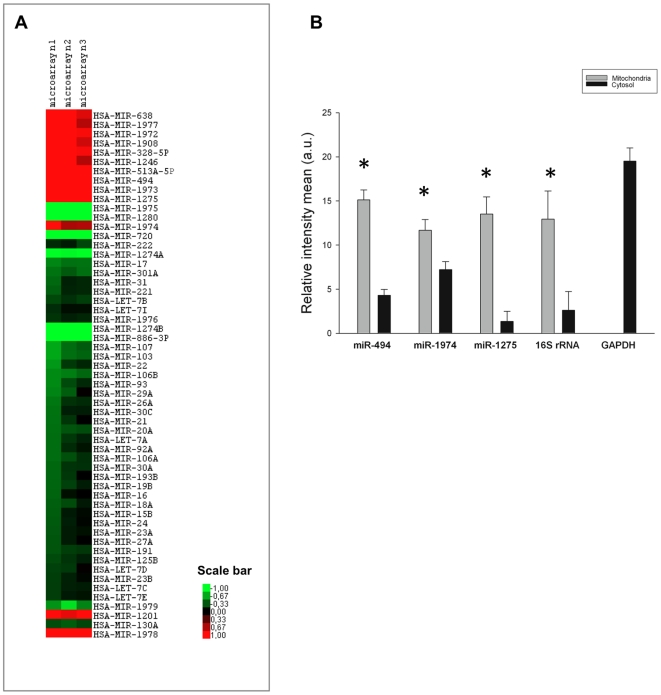
Evidence for a mitochondrial miRNA signature in HeLa cells. A. Heatmap showing the 57 miRNAs differentially expressed in HeLa cells from mitochondrial and cytosolic RNA. Three independent microarray profiling experiments (microarray n1, n2 and n3) are shown and reproducibly revealed 13 miRNAs enriched in mitochondrial RNA. Log2 Hy5/Hy3 ratios are color scaled in gradient from green (low levels) to red (high levels) as indicated by the scale bar. B. Validation of microarray data by RT-PCR. Five genes (hsa-miR-494, hsa-miR-1974, hsa-miR-1275, 16S rRNA and *GAPDH*) were selected for microarray data validation of differential expression in either subcellular compartment. 16S rRNA was used as a positive mitochondrial control, while *GAPDH* as a negative mitochondrial control. Quantitative analysis of band intensities are shown for three independent RT-PCR experiments and indicated as arbitrary units (a.u.) in either mitochondria (grey) or cytosol (black). Error bars represent the standard error of the mean. Asteriscs indicate statistically significant differences as compared to the cytosol (hsa-miR-494, p-value = 3.32×10^−5^; hsa-miR-1974, p-value = 0.02; hsa-miR-1275, p-value = 6×10^−4^; 16S rRNA, p-value = 0.03).

We profiled miRNAs at the genome-wide scale in the mitochondrial and cytosolic RNA fractions purified from HeLa cells (Figure 4A). Mitochondrial and cytosolic RNA were labeled using the fluorescent dyes Hy5 and Hy3, respectively, and hybridized to microarrays in three independent analyses. MiRNAs showing significant hybridization signals were analyzed for their enrichment either in the mitochondrial or the cytosolic RNA fractions by determining the Hy5/Hy3 ratio values. Using a cutoff fold of enrichment of 1.5, we identified a subset of 57 miRNAs differentially expressed in the mitochondrial and cytosolic RNA fractions (Figure 5A). Two subgroups were clearly identified suggesting that a specific population of miRNAs was likely compartmentalized in mitochondria. While 44 miRNAs showed a greater enrichment in the cytosolic Hy3-labeled RNA fraction, 13 miRNAs were significantly and reproducibly enriched in the mitochondrial Hy5-labeled RNA sample (ranging from 1.5- to 56-fold), namely hsa-miR-1973, hsa-miR-1275, hsa-miR-494, hsa-miR-513a-5p, hsa-miR-1246, hsa-miR-328, hsa-miR-1908, hsa-miR-1972, hsa-miR-1974, hsa-miR-1977, hsa-miR-638, hsa-miR-1978 and hsa-miR-1201 (Figure 5A). In parallel, microarray experiments were repeated thrice with RNase A-treated mitochondria, giving a consistent signature of the same mitochondrial-enriched miRNAs (Figure S1). This latter result emphasized the actual localization of those miRNAs within the mitochondria. The data are accessible through GEO Series (http://www.ncbi.nlm.nih.gov/geo/query/acc.cgi?acc=GSE24761).

Microarray data was independently verified by RT-PCR analysis assessing hsa-miR-494, hsa-miR-1275 and hsa-miR-1974. For each we assessed the differential expression in HeLa mitochondria relative to the mitochondrial control, 16S rRNA (Figure 5B). All miRNAs were significantly enriched in mitochondria as compared to the cytosol (p<0.03). Finally, we further validated our results through a systematic comparison with previously released miRNA expression data in HeLa cells, using total RNA [35], [36] (Table S2). We observed that the vast majority of cytosolic-enriched miRNAs (84%) were reproducibly identified in previous HeLa expression data while most of the mitochondrial-enriched miRNAs (69%) were absent as expected from their dilution in total RNA (Figure S2). Thus, our findings were consistent with a signature of 13 miRNAs significantly enriched in the mitochondrial RNA fraction. Notably, three of those miRNAs, namely hsa-mir-1974, hsa-mir-1977 and hsa-mir-1978 are non-canonical miRNAs for they map to the mitochondrial genome, and they map to tRNA and rRNA genes. However, their size, which ranges from 21–23 nucleotides is similar to miRNAs. The identification of several small RNA similar in size to miRNAs that are derived from abundant non-coding RNAs [37], [38] prompted us to consider hsa-mir-1974, hsa-mir-1977 and hsa-mir-1978 as potential components of RNAi at mitochondria. For simplicity, we termed those three miRNA-like RNAs and the other detected miRNAs, ‘mitomiRs’ in reference to their preferential localization in HeLa mitochondria. We examined the conservation of all mitomiRs by systematic BLAST of their sequences against those from metazoan species. To our surprise, despite the fact that most miRNAs are conserved accross metazoans [39], only two of the 13 mitomiRs, i.e hsa-miR-494 and hsa-miR-328, were highly conserved while the others were either human-specific or only conserved in primates (Table 1).

**Table 1:** Cross-species conservation of pre-miRNA sequences in metazoans.

miRNA identifier	Species (taxonomic orders)	Conservation score*
hsa-miR-1973	*Hsa (Primates)*	0
hsa-miR-1275	*Hsa, Ptr, Ppy (Primates)*	1
hsa-miR-494	*Hsa, Ptr, Ppy, Mml, (Primates), Mmu, Rno (Glires), Cfa (Carnivora), Eca, Bta (Perissodactyla), Ssc (Cetertiodactyla)*	2
hsa-miR-513a	*Hsa, Ptr, Ppy, Mml, Age, Ssy, Pbi (Primates)*	1
hsa-miR-1246	*Hsa, Ptr, Ppy (Primates)*	1
hsa-miR-328	*Hsa, Ptr, Ppy, (Primates), Mmu, Rno (Glires), Cfa (Carnivora), Eca, Bta (Perissodactyla), Ssc (Cetertiodactyla)*	2
hsa-miR-1908	*Hsa, Ppy (Primates)*	1
hsa-miR-1972	*Hsa (Primates)*	0
hsa-miR-1974	*Hsa (Primates)*	0
hsa-miR-1977	*Hsa (Primates)*	0
hsa-miR-638	*Hsa, Ppy, Mml (Primates)*	1
hsa-miR-1978	*Hsa (Primates)*	0
hsa-miR-1201	*Hsa, Ptr, Ppy (Primates)*	1

*Conservation score was assigned as follows: 0 for miRNA sequences that were human specific; 1 for miRNAs sequences conserved in primates; 2 for miRNA sequences conserved in more than 2 orders.

### MitomiRs targeting analyses

We systematically assessed the computational targeting of all mitomiRs. To obtain unbiased predictions, we analyzed the targeting of the 13 mitomiRs in parallel to a control sample of 13 cytosolic-enriched miRNAs. For each miRNA, targets were predicted (Table S3) and then analyzed for enrichment in nuclear genes coding for proteins known as mitochondrial [40]. Mean percentage of these genes within the mitomiR target set reached 17±3 versus 17.6±2.8 in the control set. This difference was not statistically significant (Table S4 and Figure S3). Thus, mitomiRs appeared to lack any preferential predicted targeting of the mitochondrial genes encoded by the nuclear genome.

**Figure 6:**
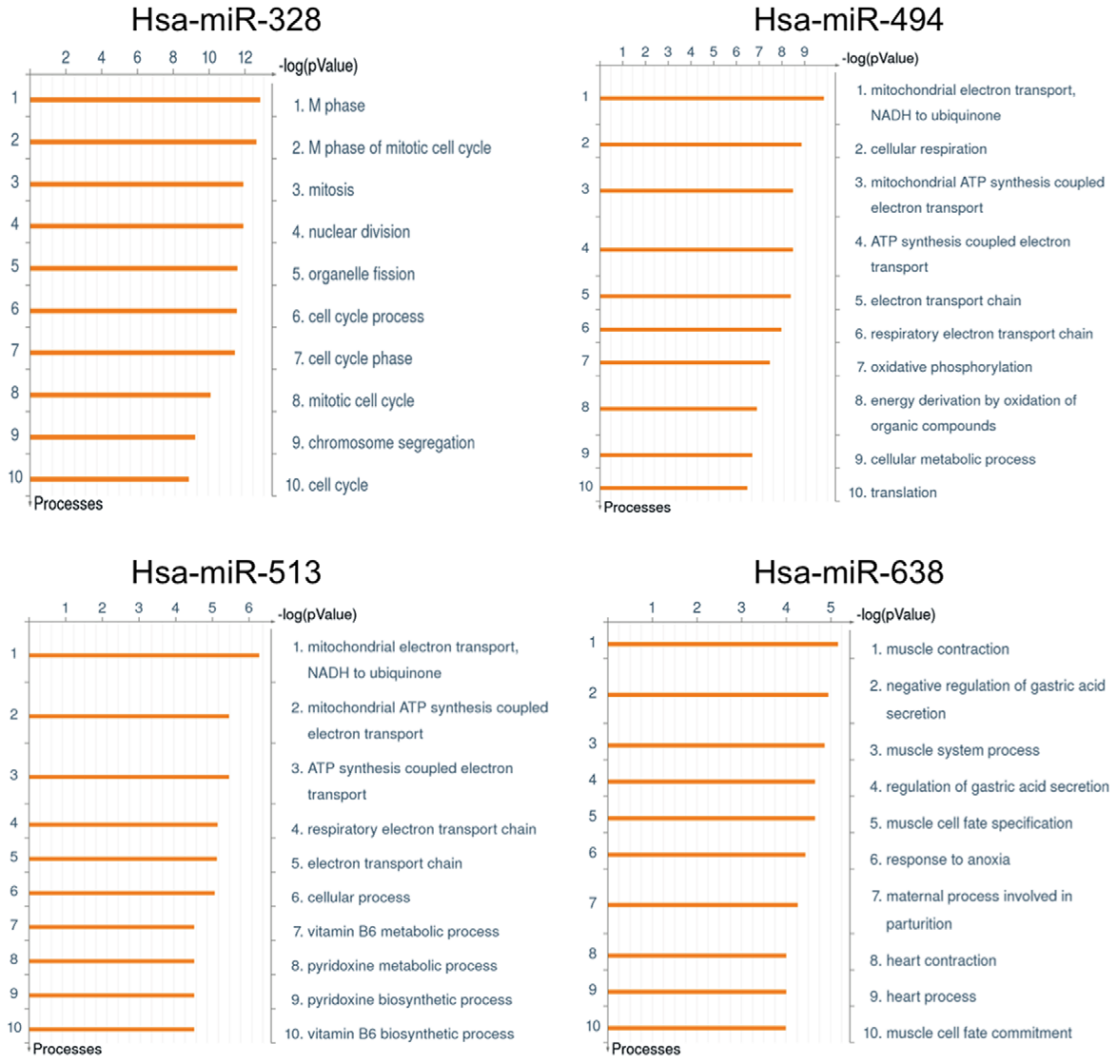
Ontology enrichment analysis for target genes of hsa-miR-328, hsa-miR-494, hsa-miR-513 and hsa-miR-638. The ExParser algorithm was used to compile datasets of genes whose expression patterns were comparable and statistically correlated to the expression patterns of the four mitomiRs. The datasets were uploaded into MetaCore™ and analyzed in respect to the Gene Ontology Process. Ten most significantly enriched processes for the genes targeted by hsa-miR-328, hsa-miR-494, hsa-miR-513 and hsa-miR-638 were scored and ranked in respect to the obtained p-values. Bars represent significance as −log(p-value) for hypergeometric distribution. Ontology enrichments were all filtered to allow no more than 5% false discovery rate.

We then questioned the functional relevance of those mitomiRs. To this end, we used the ExParser algorithm with the publicly available datasets to retrieve the mRNA genes experimentally classified as targets of miRNAs and/or as co-regulated in their expression with mitomiRs [36]. While lack of available experimental data precluded systematic questioning, we were able to analyze the target and/or co-regulated mRNAs for hsa-miR-328, hsa-miR-494, hsa-miR-513 and hsa-miR-638 (Table S5). For each mitomiR, the compiled mRNAs were then assessed for their biological significance using a systems biology pathway analysis tool [41]. We found that all four mitomiRs were significantly involved in mitochondrial homeostasis, e.g hsa-mir-494 and hsa-mir-513 are both involved in ATP synthesis coupled electron transport (Figure 6). One common feature of their involvement pointed out their role in translation initiation and in cell cycle (Figure S4). In particular, the most significant result of hsa-mir-494 is in mitochondrial translation (p-value = 3.5×10^−7^) (Figure S4).

Subsequently, we hypothesized that mitomiRs may directly affect the mitochondrial genome. To scan the mitochondrial genome for potential miRNA target sites, we used four independent and complementary algorithms RNA22, RegRNA, miRWalk and Target Scan (described in the Methods). Ten of the 13 mitomiRs were predicted to target a total of 120 target sites along the mtDNA sequence. We found target sites mapping each mtDNA-encoded protein genes, except for *ND4L* (Table 2). Merging the targeting analyses from the independent searches highlighted that the most frequent target sites were located at *ND1, ND4, ND5, ND6, COX1 and COX2* (Table 2). Interestingly, the first four genes encode the components of the first complex of the respiratory chain. However, it should be emphasized that the actual location of targets does not necessarily concern the targeted gene given the polycistronic transcription of mitochondrial genome (reviewed in [42]). To gain insight into the biological relevance of those predictions, we performed the same analysis with the control set of cytosol-enriched miRNAs, and found a preferential targeting of mitomiRs versus cytosolic miRNAs (Table S6).

**Table 2:** List of mitomiR-predicted target sites in the mitochondrial genome.

	Computational tools				
	RNA22	RegRNA	MiRWalk		Target Scan
mitomiR	Gene hosting miRNA target sites	Gene hosting miRNA target sites	Gene hosting miRNA target sites	p-value (<0.05)	Gene hosting miRNA target sites
hsa-miR-1973	-	-	-		-
hsa-miR-1275	ND5, CYTB, COX2, ATP8, ND2, COX1, D-loop	**ND4**, **ND5**, D-loop	**ND6**	0.008	COX2, ND4, ND6
hsa-miR-494	-	-	-		-
hsa-miR-513a-5p	-	-	-		-
hsa-miR-1246	ND5, COX1	-	**COX1**	0.002	ND5, COX1
hsa-miR-328-5p	RNR1	-	-		-
hsa-miR-1908	ND6, CYTB, ND4, COX2, ND1	COX1, RNR2, D-loop, ND3, TRNN, ND5, ND4, ATP8/ATP6	COX1	0.02	D-loop, RNR2, COX1, ND3
hsa-miR-1972	COX1, COX3, L-strand replication origin region	**COX1**	-		COX1
hsa-miR-1977	ND4, TRNN, TRNP, ND2, RNR2, ND5, TRNL2	**TRNN**	-		TRNN, COX1, ND5, CYTB
hsa-miR-638	ATP6, TRNP, TRNY, D-loop, COX1, ND5, ND2, CYTB, ATP8, ND1, ND3, RNR2	**COX2**	COX2	0.01	COX2
hsa-miR-1974	ND6/TRNE, ND5, CYTB, ND4, ATP6, TRNS2/TRNL, ND1	**ND6/TRNE**, **ND1**	-		Dloop, ND1, ND2, ND5, TRNE
hsa-miR-1978	ND1, COX2, COX1	-	-		Dloop, RNR2, ND2, COX1, COX2
hsa-miR-1201	TRNF, COX2, ATP6	-	-		-

– indicates the lack of targeting sites predicted at the level of mitochondrial genome.

The mitochondrial genes indicated in bold present overlapping target sites between RegRNA and RNA22 or RegRNA and miRWalk. The (promoter or mRNA sequence) is calculated by. P-values were calculated using Poisson distribution as a probability distribution of random matches of a subsequence (miRNA 5′ end sequence) in the given sequence calculated. Low probability implies a significant hit (p<0.05).

### Genomics and intrinsic features of the mitomiRs

To gain insights into the molecular basis underlying the mitochondrial localization of miRNAs, we first questioned their genomics. Since it is widely accepted that most miRNAs share regulatory elements with their genomic environment, and when intragenic are typically co-processed from the host gene mRNAs, we inferred that the genomic location of mitomiRs might be informative [43]. Interestingly, the 13 mature mitomiRs appear to be expressed from 15 miRNA genes (Table 3). Of those 15 genes, 9 were intragenic while 6 were intergenic (Table 3). We found that genomic locations of the mitomiRs, besides hsa-miR-513a and hsa-miR-1275 were all relevant to mitochondria. In particular, of 9 intragenic mitomiRs, 4 were hosted in mitochondrial genes (Table 3). Strikingly, hsa-miR-1974, hsa-miR-1977 and hsa-miR-1978 also exhibited a perfect match in the mitochondrial genome with two mitochondrial tRNA genes, *TRNE* and *TRNN* and with a stretch of the mitochondrial rRNA sequence *RNR1*, respectively. Thus, in addition to the nuclear transcription that can be assumed from the detection of hsa-miR-1974 in the cytosol (Figure 5B), it will remain to be ascertained whether the transcription of those 3 mitomiRs could also occur from the mitochondrial genome.

**Table 3:** Genomic and chromosomal locations of mitomiRs.

miRNA (miRBase v.13)	Genomic location (hg19)	Chromosomal location (hg19)	Host gene	Genomic link to mitochondria - Evidence
hsa-miR-1973	Chr4: 117220905–117220924 (+)	4q26	Intergenic	Embedded in a region of mitochondrial pseudogenes.
hsa-miR-1275	Chr6: 33967794–33967810 (−)	6p21.31	Intergenic	None.
hsa-miR-494	Chr14: 101496018–101496039 (+)	14q32.31	Intergenic	Embedded in a syntenic conserved region of mitochondrial carrier proteins [75].
hsa-miR-513a	(1) Chr X: 146295056–146295073 (−)	Xq27.3	Intergenic	Localization (1) and (2): none.
	(2) Chr X: 146307418– 146307435 (−)			
hsa-miR-1246	Chr2: 177465752–177465770 (−)	2q31.1	Intergenic	Embedded in a region of mitochondrial genes – Locus involved in mitochondrial disorders (EOMFC).
hsa-miR-328	Chr16: 67236230–67236251 (−)	16q22.1	Intragenic	Locus involved in mitochondrial disorders (CDG2H, MDDS2).
hsa-miR-1908	Chr11: 61582681–61582701 (−)	11q12.2	Intragenic	Hosted in FADS1 intron (mitochondrial localization [76]).
hsa-miR-1972	(1) Chr16: 151041224–15104246 (−)	(1) 16q13.11	Intragenic	Localization (1): None.
	(2) Chr16: 70064295–70064317 (+)	(2) 16q22.1		Localization (2): Locus involved in mitochondrial disorder (CDG2H, MDDS2).
hsa-miR-1974	Chr5: 93905172–93905194 (−)	5q15	Intragenic	Hosted in mitochondrial pseudogene (AC093311.4) and embedded in a region of mitochondrial pseudogenes. Likely transcription from the mitochondrial genome (TRNE, M: 14675–14697 (−)).
hsa-miR-1977	Chr1: 566242–566263 (−)	1p36.33	Intragenic	Hosted in mitochondrial pseudogene (AC114498.7) and embedded in a region of mitochondrial pseudogenes. Likely transcription from the mitochondrial genome (TRNN, M:5693–5714 (−)).
hsa-miR-638	Chr19: 10829095–10829119 (+)	19p13.2	Intragenic	Hosted in DNM2 (mitochondrial localization [40]).
hsa-miR-1978	Chr2: 149639365–149639385 (−)	2q23.1	Intragenic	Possible transcription from the mitochondrial genome (RNR1, M: 654–674 (+)).
hsa-miR-1201	Chr14: 19864456–19864479 (−)	14q11.2	Intragenic	Locus involved in mitochondrial disorders (PCK2D).

**Figure 7:**
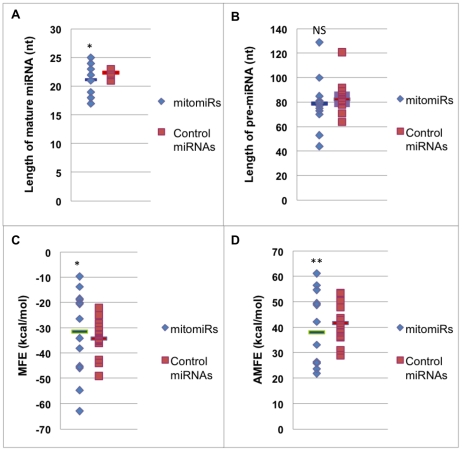
Intrinsic features of mitomiRs versus a control sample of cytosolic-enriched miRNAs. Values for each miRNA subgroup are shown in blue for the mitomiRs and red for the control miRNAs. Length of mature miRNAs (A) and of pre-miRNAs (B), and values of minimal folding free energy (MFE) (C) and adjusted MFE (AMFE) (D) are plotted for each miRNA from the 2 subgroups. Average values are shown as bars in each subgroup. Asterics indicate significant differences between mitomiRs versus control miRNAs (p-value<0.05). NS indicates not significant p-value.

Secondly, we systematically analyzed the intrinsic features of mitomiRs. We assessed their lengths and thermodynamic features in comparison to the same control sample of 13 cytosolic-enriched miRNAs. Unequivocally, the control sample shared all expected features of miRNAs, in particular an average length of 22-nt for the mature and of 82-nt for the pre-miRNA sequence (Figure 7A). In contrast, the length of the mature mitomiRs varied substantially. Three mitomiRs were smaller than 19 nt (Table S7). In fact, the length distribution of the mature mitomiRs was significantly different from the control (p<0.005) but did not correlate with a difference at the pre-miRNA sequence level, which remained comparable (p = 0.2) (Figure 7A–B). Thus, we assessed the thermodynamic stability of the secondary structures of the mitomiRs by calculating their minimum folding energy (MFE). MFE displayed a significantly different distribution than the control group (p = 0.01) (Figure 7C, Table S7, and Table S8). Because MFE is strongly correlated with the length of the sequence, we also calculated the adjusted MFE (AMFE) (Table S7). Again, the distribution of AMFE values was significantly different from that of the control miRNAs (p<0.05), which revealed that mitochondrial-enriched miRNAs shared specific distinctive features as a group (Figure 7D, Table S7 and Table S8).

Altogether, our findings illustrate a species-specific signature of mitochondrial miRNAs of unusual sizing that exhibit unique thermodynamic features. These results suggest a set of emerging features of structural properties that should help in the identification of other mitomiRs.

## Discussion

**Figure 8:**
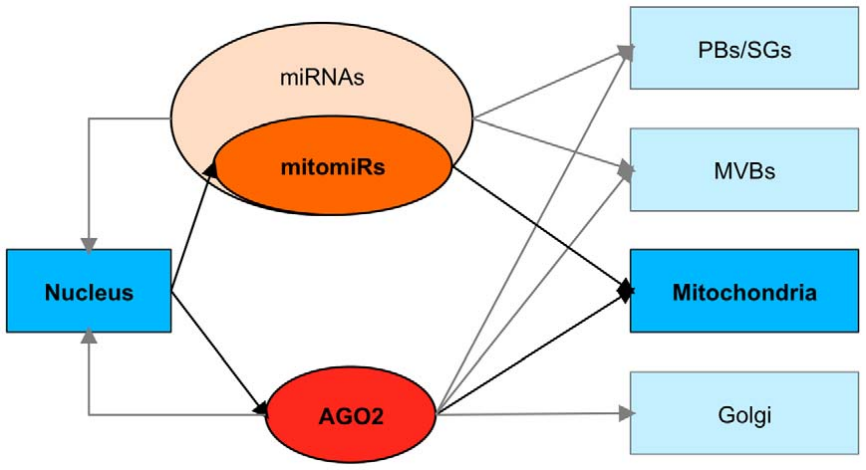
Integrative model for subcellular localization of RNAi components. Boxes represent the subcellular compartments. RNAi components are encircled. Arrows indicate the molecular fluxes documented herein in regards to the mitochondrial miRNAs (mitomiRs), and previously in P-bodies (PBs) [17], stress granules (SGs) [16], [54], multivesicular bodies (MVBs) [21] and Golgi [26]. Depending on the localization, different functions are ascribed to AGO2, such as post-transcriptional gene silencing or reversible translational regulation in P-bodies [50] and stress granules [16], and transcriptional regulation in the nucleus [51]. In mitochondria, the presence of mitochondrial genome adds another possible layer of regulation by AGO2.

Little is known about the crosstalk between the nucleus and mitochondria, despite the fact that this communication has great relevance for understanding integrative cellular signaling pathways. In this report, we have used complementary approaches to provide evidence that all components of RNA interference are present at the mitochondria in human cells (Figure 8).

We identified a set of 13 miRNAs significantly enriched in mitochondria purified from HeLa cells that we referred to as mitomiRs. We believe that our experimental design, focusing on the differential expression of miRNAs in subcellular fractions isolated from the same cells, favored the identification of a specific signature of miRNAs in mitochondria. Recently, comparable approaches were successfully used for the identification of nuclear and nucleolar small RNAs [19], [44]. In our methodology, the choice of immunoisolating mitochondria after differential centrifugation gave us the additional opportunity to wash the organelles in stringent conditions, leading to highly purified mitochondrial fractions. Our findings not only identify a novel set of mitochondrial miRNAs in humans, but also confirm the previous finding of enrichment of miR-494 in rat liver mitochondria [23]. Our analysis of genes hsa-mir-494 functionally-related revealed its involvement in regulating translation in mitochondria. Thus beyond a role in the cytosol, hsa-miR-494 likely displays conserved functions relevant to its mitochondrial localization.

Our study further introduces the idea that a nuclear outsourcing of miRNAs to mitochondria is conserved in mammals. Indeed, extensive mapping of our mitomiRs, as well as the mitochondrial miRNAs identified in rat liver [23] (data not shown) demonstrated that they mostly originated from the nucleus. Noteworthy, hsa-miR-1974, hsa-miR-1977 and hsa-miR-1978 are considered non-canonical miRNAs because they map to mitochondrial tRNA and rRNA genes. At this stage, our data on other mitomiRs and AGO2 at the mitochondria prompt to gain more insights into the accuracy of those three miRNA-like RNA production, whether from nuclear or mitochondrial genome, and from specific enzymatic cleavage or from random degradation. As for hsa-miR-1201, which is also questioned as a genuine miRNA due to the overlap with an annotated small nucleolar RNA (SNORD126), we considered that its binding to both AGO2 and mRNAs [45] sufficiently argued for its possible functioning as a mitomiR. Although the mechanism of miRNA localization to mitochondria is unclear at this stage, our analysis suggests that their hosting in specific genomic regions, together with their lengths and thermodynamics may play a significant role in the specificity of this subcellular localization. Our observation also indicates that most human mitomiRs are not conserved beyond primates, which may suggest a species-specific targeting of miRNAs to human mitochondria. We propose that this biased lack of conservation throughout species likely relates to the peculiar evolutionary pressure that concerns mitochondrial genomes [46], [47]. We speculate that further investigation specifically on hsa-miR-1974, hsa-miR-1977 and hsa-miR-1978, which are human-specific only may greatly serve our understanding into both the origin and evolution of miRNAs.

The actual mitochondrial localization of some miRNAs implies that small RNA-mediated processes may regulate mitochondrial biogenesis and function. This hypothesis involving an additional level of nuclear control of mitochondria is emphasized by our finding that AGO2 localizes to mitochondria, and that we and, others, identified its binding to the mitochondrial transcripts *COX3* and tRNA^Met^ [24], [33]. AGO2 may be considered as a versatile protein, localized to several sites within the cell, including unstructured foci and vesicles [16], [17], [21], [48], the Golgi apparatus and the endoplasmic reticulum [26] but also the nucleus [20]. Yet, it is not clear which of these cellular structures are necessary for AGO2 functions [49]. Depending on its localization, different functions have already been ascribed to AGO2, such as post-transcriptional gene silencing or reversible translational regulation in P-bodies [50] and stress granules [16], and transcriptional regulation in the nucleus [51]. Given the unique features of mitochondria in terms of genomic organization and regulation [52], it is difficult at this stage to predict role of AGO2 at the mitochondria. Here, our prediction that the isoforms of AGO2 may be differently regulated in respect to their localization to mitochondria adds an additional layer of complexity. Which of the AGO2 isoforms actually localizes to mitochondria, where in the mitochondria and what is the underlying mechanism of such mitochondrial targeting remain to be explored in future studies. In this respect, interesting insights come from the proteomic studies of AGO2 partners, which identified mitochondrial proteins mostly from the inner membrane, including many ATP/ADP translocases, carriers and ribosomal proteins as binding partners [53]. These data, altogether with the recent finding that AGO2 also associates with HSP90 [54] provide interesting rationales as to a possible mechanism for a mitochondrial import of AGO2. Furthermore, our computational identification of miRNA targets in the mitochondrial genome actually provided the first step towards elucidating the functions of AGO2 at the mitochondria. However, technical limitations of directly transfecting mitochondria *in vivo* will make it challenging to test the classical approaches to characterize the regulatory role of mitomiRs and AGO2 at the mitochondria.

To conclude, the nuclear outsourcing of miRNAs and AGO2 at the mitochondria is likely an additional mechanism mediating the crosstalk between the nucleus and mitochondria (Figure 8). Based on our study, consideration of the mitomiRs as a new species for mitochondrial regulatory RNAs may lead to a deeper understanding of signaling pathways that require nuclear-mitochondrial cooperation. Elucidating the preferential distribution of miRNAs to mitochondria should then provide a first framework to further investigate their organelle-specific functions and to unravel their potential in devising new therapeutic strategies.

## Materials and Methods

### Genomic and protein sequences

NCBI entries of the protein sequences of human AGO2 (also referred to as EIF2C2) were systematically retrieved (Gene Identity: 6468775, 14043279, 62913977, 119612613, 29171734, 257467482, 133777965 and 119612614). The dataset for miRNA sequences were downloaded from the Sanger miRBase database Release 13.0 [55]. The studied mitochondrial genomic DNA (mtDNA) sequence was retrieved from NCBI (NC_012920).

### Genomic and protein bioinformatic analyses

Genomic and chromosomal locations were analyzed through the Ensembl and UCSC genome browsers (release 59), and our in-house developed bioinformatic tool to question miRNAs and genetic loci/diseases (http://www.mirifix.com, Henrion-Caude personal communication). Mapping of the mature miRNA sequences on genomic nuclear and mitochondrial DNA was performed using BLAT [56]. Conservation of each miRNAs was assessed through BLASTN with default parameter values [55].

Molecular weight of each AGO2 entry was calculated using the ProtParam tool [57] to identify the adequate protein sequences to be further analyzed. Predictions of subcellular localization were done using the following three network-based approaches, namely TargetP 1.1 [29], MitoPROT II v. 1.101 [30], PREDOTAR 1.03 [31], and a support vector machine-based method integrative of multiple features of the protein: physicochemical properties, amino acid compostion, dipeptide compostion of proteins and PSI-BLAST information [32].

### Cell culture

HeLa cells were grown in DMEM medium completed with 10% fetal bovine serum and 100 U/ml penicillin-streptomycin. Similar conditions of culture were used for HEK293 and U2OS cells. Cell lines were purchased from ATCC (ATCC, Manassas, VA, USA). For cell fractionation, HeLa cells were allowed to reach 80–100% confluence. For immunocytochemistry, cells were allowed to reach 60% on glass coverslips.

### Isolation of mitochondria and cytosol

We isolated mitochondria with the Mitochondria Isolation kit (Miltenyi Biotec, Bergisch Gladbach, Germany) as described [25], with several modifications that allowed the isolation of the cytosol from the same cells. Briefly, HeLa cells were harvested at 80–100% confluency and washed twice with phosphate-buffered saline (PBS). Cells (3×10^7^) were lysed in 1 ml of Lysis Buffer from the kit, complemented with Complete Protease Inhibitor Cocktail Tablets (Roche, Mannheim, Germany). Then, the lysate was divided into two aliquots. In order to isolate mitochondria, the first aliquot was homogenized by shearing through a 29G needle 55 times. To magnetically label mitochondria, the cell lysate was incubated with 50 µl of monoclonal anti-TOM22-conjugated microBeads for 1 hour at 4°C. Then, the suspension of labeled mitochondria was loaded onto a pre-equilibrated MACS Column (Miltenyi Biotec, Bergisch Gladbach, Germany), previously placed in the magnetic field of a MACS Separator (Miltenyi Biotec,Bergisch Gladbach, Germany). Due to the strong magnetic field generated by the MACS Separator, labeled-mitochondria were retained into the column. The column was washed three times with 3 ml of PEB buffer (PBS pH 7.2, 2 mM EDTA and 0.5% BSA). Subsequently, the column was removed from the magnetic field and the retained mitochondria were eluted in 1.5 ml of PEB buffer. After elution, mitochondria were suspended again in 9 ml of PEB buffer, applied to a fresh column, washed and eluted for a second time. When noted, mitochondria were treated with RNase A. Briefly, isolated mitochondria were resuspended in suspension buffer (0.25 M sucrose, 2 mM MgCl2, 10 mM Tris HCl pH 7.4) and treated with 50 µg protease-free RNase A (USB-Amersham) either for 15 min at room temperature as described in [58], or for 30 min at 4°C as described in [23] and washed twice in suspension buffer. Mitochondrial pellet was recovered after centrifugation at 13,000× g for 2 min at 4°C. Mitochondrial integrity was monitored after resuspension of the mitochondrial pellet by measuring citrate synthase activity, before and after membrane disruption by adding Triton X-100, as previously described [59].

Mitochondrial fraction of U2OS was isolated using standard method (protocol Mitosciences, adapted from [60]). Extraction of mitochondrial soluble proteins from integral membrane proteins was performed through sodium carbonate treatment as described [61].

In order to isolate the cytosolic fraction, the second aliquot of crude HeLa lysate was homogenized through a glass homogenizer in homogenization buffer (210 mM manitol, 70 mM sucrose, 1 mM EDTA, 10 mM Hepes-NaOH, pH 7.5), as previously described [62]. The homogenate was centrifuged at 2,000× *g* for 30 min at 4°C to remove nuclei and unbroken cells. The supernatant was recovered and subsequently centrifuged at 13,000× *g* for 10 min to give the cytosolic fraction, which was subjected to RNA extraction.

### Protein and RNA extraction

For total protein extraction, HeLa cells were lysed as previously described [33]. Pelleted mitochondria were lysed as described previously [63]. For RNA extraction, pelleted mitochondria and cytosolic fractions were subjected to RNA isolation by Trizol and Trizol LS (Life Technologies, Carlsbad, USA) extraction methods, respectively, according to instructions of the manufacturer.

### Microarray miRNA profiling

MicroRNA microarray analysis was performed by Miltenyi Biotec Company (Milteny Biotec, Bergisch Gladbach, Germany). Briefly, the concentration of mitochondrial and cytosolic RNAs was measured by spectrophotometry at *A*260/280 and the quality of the RNA sample was assessed using the Agilent 2100 Bioanalyzer (Agilent Technologies, Palo Alto, USA). 2 µg of respective mitochondrial and cytosolic RNA were each mixed with 2.5 fmol of miRControl 3, which comprises 18 RNA oligonucleotides, used as calibrators for normalization. Then mitochondrial RNA was Hy5-labeled while cytosolic RNA was Hy3-labeled using a commercial kit (miRCury™ LNA microRNA Array Power labeling kit, Exiqon, Copenhagen, Denmark). The corresponding total RNA samples were hybridized overnight in a dual colour approach to miRXplore™ Microarrays using the a-Hyb™ Hybridization Station (Miltenyi Biotec, Bergisch Gladbach, Germany). The miRXplore™ Microarray is composed of 6228 DNA oligonucleotides with a sequence being reverse complement to the respective mature miRNA or control RNA, representing a total of 1460 miRNA sequences and 97 controls with all probes spotted in quadruplicates. The 1460 miRNA sequences covered sequence verified miRNAs of the species human (878) mouse (696) and rat (426) as well as viral miRNAs (141) and further mammalian species (76) as deposited in the miRBase sequence database version 13.0. Image capture of microarrays was done with the Agilent's Microarray Scanner System (Agilent Technologies, Palo Alto, USA). Signal quantification of the scanned microarrays was done using ImaGene software Version 8.0 (BioDiscovery, Los Angeles, USA). The data generated for each sample on the array were analyzed with PIQOR Analyzer software. Local background was subtracted from the signal to obtain the net signal intensity and the Hy5/Hy3 ratios. Subsequently, the mean of the ratios of 4 corresponding spots representing the same miRNA was computed. The mean ratios were normalised using the miRControl 3.

### Primers, reverse transcription and PCR experiments

For miRNAs, primers were designed as the exact mature miRNA sequences (as indicated at the Sanger miRBase database, v13.0), in comparison to the 16S rRNA (5′-TATACCCACACCCACCCAAG-3′). Oligonucleotides for *GAPDH* were designed through the Primer3 Tool [64] as follows: forward 5′-CGACCACTTTGTCAAGCTCA-3′ and reverse 5′-AGGGGTCTACATGGCAACTG-3′, and used at 60°C for annealing. Primer sequences for *COX3* and cyt *b* were designed as described [65], respectively used at 48°C and 58°C for annealing. Each oligonucleotide was applied at a final concentration of 0.4 µM. Reverse-transcription of 1 µg of mitochondrial and cytosolic RNA after treatment with the RQ1 RNase-free DNase I (Promega, Madison, USA) was performed using the miScript Reverse transcriptase kit (Qiagen, Weiden, Germany) according to manufacturers' instructions. PCR were performed in triplicate in 20 µl final volume. Specificity of each PCR products was ensured by the results of melting curve analysis and agarose gel electrophoresis. Separated products were quantitated by band densities using the ImageJ software and normalized to ethidium bromide staining.

### Antibodies, immunoblotting and immunocytochemistry

The following primary antibodies were commercially purchased: mouse monoclonal anti-ATP5A1, MS507, Mitosciences, Eugene, USA, 1∶1000 for immunoblotting and 1∶750 for immunofluorescence; mouse monoclonal anti-NDUFA9, MS111, Mitosciences, Eugene, USA, 1∶5000; mouse monoclonal anti-VDAC1, MSA03, Mitosciences, Eugene, USA, 1∶2500; mouse monoclonal anti-CDK2, sc-6248, Santa Cruz Biotechnology, Santa Cruz, USA, 1∶1000; mouse monoclonal anti-actin, VMA1501R, AbCys, Paris, France, 1∶500; rabbit monoclonal anti-cythocrome c, 1896-1, Epitomics, Burlingame, USA, 1∶2000 and rabbit polyclonal anti-SLUG, sc-15391X, Santa Cruz Biotechnology, Santa Cruz, USA, 1∶250. Rabbit polyclonal anti-AGO2 was kindly provided by Pr Tom Hobman (clones 7C6 and 7C3) and the antibody was used at a dilution of 1∶1000 for immunoblotting and 1∶250 for immunofluorescence and co-immunoprecipitation. Mouse monoclonal anti-AGO2 was kindly provided by Pr Haruhiko Siomi and Pr Mikiko C. Siomi (clone 4G8) and the antibody was used at a dilution of 1∶50 for immunofluorescence and at a dilution of 1∶300 for Western blot. Secondary antibodies HRP-conjugated anti-mouse and anti-rabbit were purchased (GE Healthcare Bio-Sciences, Little Chalfont, UK). Fluorescein isothiocyanate (FITC)- and tetramethylrhodamine isothiocyanate (TRITC)-labeled secondary antibodies for immunofluorescence microscopy were from Invitrogen (Life Technologies, Carlsbad, USA). Equal amounts of protein (40 µg) were size-separated through a 3–8% sodium dodecyl sulfate-polyacrylamide gel electrophoresis (NuPage Novex Mini gel; Life Technologies, Carlsbad, USA) and were transferred to PVDF membranes (Bio-Rad, Hercules, USA). Membranes were blocked with 4% fat-free milk diluted in PBST (0,05% Tween-20, 1× PBS) for 1 hour at room temperature and incubated with primary antibodies. Antigen-antibody complexes were detected by incubating the membrane with the appropriate secondary antibodies at room temperature for 1 hour. Immunoreactive proteins were visualized with enhanced chemiluminescence and exposed to autoradiographic film (Amersham, GE Healthcare Bio-Sciences, Little Chalfont, UK). Immunocytochemical experiments were performed in HeLa, HEK293 and U2OS cells (American Type Culture Collection (ATCC): CCL-2, Manassas, VA). Mitochondria were stained with Mitotracker Red CMXRos (Molecular Probes; Life Technologies, Carlsbad, USA) before fixation according to manufacturer's instructions. In all experiments cells were fixed with 4% PFA or 2% formaldehyde for 20 min at room temperature. Cells were then washed three times with PBS, incubated with primary antibodies (1 hour at room temperature), washed, incubated with appropriate secondary antibodies (1 hour at room temperature), washed and mounted with Prolong Gold Antifade medium with Dapi (Life Technologies, Carlsbad, USA). In all experiments, the following controls were included: (i) to assess possible autofluorescence of the samples, the primary and secondary antibodies were omitted in the incubation steps; (ii) to assess possible autofluorescence of secondary antibodies, the primary antibodies were omitted in incubation step. Images were acquired using a confocal microscope Leica SP5. Co-localization of AGO2 with mitochondria was assessed by statistical analysis of the correlation of the intensity values of green (AGO2) and red (mitochondria) pixels in dual-channel images. The JACoP plug-in from ImageJ software was used to calculate the Pearson's correlation coefficient (*r*p) and to perform cross-correlation analysis (Van Steensel's cross-correlation function, CCF) [28], [66], [67]. The Pearson's correlation coefficient (*r*p) describes the correlation of the distributions of signal intensity of pixels between green and red channels [60]. It lies between +1 and −1. From 0 to +1, values indicate significant correlation of green and red images, with 1 indicating 100% co-localization. In the Van Steensel's approach, the CCF is calculated as the value of *r*p while operating a shift of one of the images relative to the other, then plotting the retrieved *r*p as a function of the displacement. Co-localization is identified by a Gaussian distribution, while in the opposite situation, the curve appears as a hollow.

### Co-immunoprecipitation of mitochondrial transcripts

Co-immunoprecipitation of AGO2 with associated transcripts was performed using μMACS protein A microbeads (MiltenyiBiotec, Bergisch Gladbach, Germany) according to manufacturer's instructions with the following modifications. Briefly, proteins were incubated with protein A microbeads and either rabbit polyclonal anti-AGO2 (clone 7C6 or 7C3) or rabbit polyclonal anti-SLUG as a negative control (sc-15391X, Santa Cruz Biotechnology, Santa Cruz, USA) for 1 hour at 4°C on ice. After 6 washes of varying stringency onto microcolumns, proteins were treated with proteinase K in washing buffer complemented with 0.1% SDS, 25 mM EDTA for 30′ at 42°C. Transcripts were then eluted at 80°C with pre-heated RNase-free water. Subsequently, RNA was extracted with Trizol reagent (Lifetechnologies, Carlsbad, USA) (according to manufacturer's protocol), treated with the RQ1 RNase-free DNase I (Promega, Madison, USA) (according to manufacturer's instructions), reverse transcribed with the GeneAmp RNA PCR kit (Applied Biosystems, Life Technologies, Carlsbad, USA) (according to manufacturers' instructions), and subsequently amplified by PCR using standard conditions.

### Structural analyses of miRNAs

The minimal folding energy (MFE), expressed in kcal/mol, is a method of calculating the thermodynamic stability of the secondary structure of RNA [68]. The lower the MFE of a molecule, the more stable the secondary structure. Minimal folding free energies (MFEs) of pre-miRNAs were estimated using the program RNAfold (Vienna RNA Package, version 2.0.0) with default parameter values [69], [70]. Because MFE values are strongly correlated with the length of the sequence we normalized the MFE by calculating the adjusted MFE (AMFE) using the following equation: AMFE = [(−MFE/length of RNA sequence)×100] [71]. MFE index (MFEI) was calculated using the following equation: MFEI = AMFE/(G+C)% [71].

### Computational prediction of miRNA targets

For each miRNA, genome-wide miRNA nuclear targets were determined using miRDB as a tool of primary focus on mature miRNAs, which are the functional carriers of miRNA-mediated regulation of gene expression [72]. Enrichment in mitochondrial protein-coding gene was identified from the overlap between predicted targets and mitochondrial proteome [40]. To scan the mitochondrial genome for potential miRNA target sites, we used four independent algorithms RNA22, Target Scan, RegRNA and miRWalk. RegRNA is based on the miRanda algorithm, which relies on both the complementarity of miRNA and target sequences and on the conservation of the target site [73]. Target Scan algorithm (http://www.targetscan.org/) searches for the presence of conserved target sites that match the seed region of each miRNA and assesses the structural accessibility of the predicted target site. RNA22 is based on the Teiresias algorithm, which relies on a pattern-based approach without using conservation filters [74]. The miRWalk algorithm is based on a computational approach starting with a heptamer seed of miRNA and identifies possible complementary on the complete mitochondrial genome (Ruprecht-Karls-Universität Heidelberg, Medizinische Fakultät Mannheim, Germany). A probability distribution of random matches of a subsequence (miRNA 5′ end sequence) in the given sequence was calculated by using Poisson distribution where a low probability implies a significant hit. All predictions algorithms were run under default parameters.

### Expression data and pathway database analysis

All data is MIAME compliant and the data presented in this manuscript have been deposited in NCBI's Gene Expression Omnibus. miRNA expression profiling data was accessed through mimiRNA [36] and MirZ [35]. The ExParser algorithm [36] was used to compile datasets of gene that multiple experimental sources classified as targets of one mitomiR and/or as genes co-regulated with the mitomiR. The datasets obtained for each miRNA were uploaded into MetaCore™, a systems biology pathway analysis tool [41]. Ontology enrichment analysis was performed using general enrichment categories, i.e. Gene Ontology and GeneGo™ Processes, which represents prebuilt networks of manually curated protein-protein, or protein-nucleic acid interactions, assembled on the basis of proven literature evidence. The enrichment calculation uses the Fisher exact test or hypergeometric distribution to calculate the probability that the degree of overlap between the list of miRNA targets (generated from the ExParser query), and the protein represented in the functional ontology category can happen by chance, given an identical number of proteins selected at random from the protein universe annotated within the ontology. The p-value generated is used to rank the functional representation of the miRNA targets in each ontology by their significance to the list of targets, thereby identifying biological process likely to be affected. Probabilities were calculated according to the manufacturer's recommendations.

### Statistics and microarray analyses

Statistical significance was validated using a two-tailed Student's t-test assuming unequal variance, respectively a Fisher-Snedecor F-test for variance distribution whereby significance was achieved for p<0.05 in each test. Data analysis of the microarray was performed as follows: first, scan images of the microarrays were analyzed using the ImaGene Software (Biodiscovery). During the image analysis, irregular spots, dust particles or areas of high background were discarded, and exact position of each spot, its identity, its signal intensity, and surrounding background are saved. Second, primary data analysis was then performed calculating the net signal intensities (spot signal intensity minus background signal intensity). As filter criteria, we applied the 50% percentile of the background signal intensities. For calculation of the Hy5/Hy3 ratio, only spots/genes with signal equal or higher than the 50% percentile of the background signal intensities were taken into account. For each microarray this Hy5 and Hy3 default/threshold value was adjusted by the median of the calibrators. Third, data were normalized to correct for dye bias such as inconsistent labelling efficiencies, varying quantum yields of the dyes, or different scanning parameters. To overcome these systematic variations a normalization using spiked calibration controls was performed. After normalization, ratios of the mitochondrial vs. cytosol for each spot were calculated, and subsequently the mean of the ratio from 4 spots was calculated. Ratios were calculated for values above the adjusted threshold value (calculated based on the 50% percentile of the background signal intensities and adjusted by the mean of the calibration oligos): avg(Hy5-bkg)<adjust_Hy5_default AND avg(Hy3-bkg)<adjust_Hy3_default.

## Supporting Information

Figure S1 MiRNA profiling of HeLa mitochondria with or without RNase A treatment.
(PDF)

Figure S2 Comparison of miRNA expression data in HeLa cells.
(TIFF)

Figure S3 Mitochondrial enrichment among predicted miRNA targets of mitomiRs and control miRNAs.
(TIFF)

Figure S4 Ontology enrichment analysis for target genes of hsa-miR-328, hsa-miR-494, hsa-miR-513 and hsa-miR-638.
(TIFF)

Supporting Information S1 Measurement of enzymatic activities in mitochondrial fraction.
(DOC)

Table S1 Prediction of subcellular localizations of AGO2.
(DOC)

Table S2 Compiled miRNA expression profiling data in HeLa cells.
(DOC)

Table S3 Nuclear-encoded computational target genes of mitomiRs and control miRNAs.
(DOC)

Table S4 Mitochondrial enrichment in miRNA targets predicted for mitomiRs and control miRNAs.
(DOC)

Table S5 Compilation of target genes and/or genes co-regulated with hsa-miR-328, hsa-mir-494, hsa-mir-513 and hsa-mir-638.
(DOC)

Table S6 Number of miRNA target sites on the mitochondrial genome predicted for mitomiRs and control miRNAs.
(DOC)

Table S7 Lengths and thermodynamic features of mitomiRs.
(DOC)

Table S8 Comparison of MFE, AMFE, MFEI of mitomiRs and control miRNAs.
(DOC)

## References

[1] Entelis NS, Kolesnikova OA, Martin RP, Tarassov IA (2001). RNA delivery into mitochondria.. Adv Drug Deliv Rev.

[2] O'Brien TW, Denslow ND, Anders JC, Courtney BC (1990). The translation system of mammalian mitochondria.. Biochim Biophys Acta.

[3] Chang DD, Clayton DA (1987). A mammalian mitochondrial RNA processing activity contains nucleus-encoded RNA.. Science.

[4] Li K, Smagula CS, Parsons WJ, Richardson JA, Gonzalez M (1994). Subcellular partitioning of MRP RNA assessed by ultrastructural and biochemical analysis.. J Cell Biol.

[5] Topper JN, Clayton DA (1990). Secondary structure of the RNA component of a nuclear/mitochondrial ribonucleoprotein.. J Biol Chem.

[6] Magalhaes PJ, Andreu AL, Schon EA (1998). Evidence for the presence of 5S rRNA in mammalian mitochondria.. Mol Biol Cell.

[7] Smirnov A, Tarassov I, Mager-Heckel AM, Letzelter M, Martin RP (2008). Two distinct structural elements of 5S rRNA are needed for its import into human mitochondria.. Rna.

[8] Rubio MA, Rinehart JJ, Krett B, Duvezin-Caubet S, Reichert AS (2008). Mammalian mitochondria have the innate ability to import tRNAs by a mechanism distinct from protein import.. Proc Natl Acad Sci U S A.

[9] Bartel DP (2004). MicroRNAs: genomics, biogenesis, mechanism, and function.. Cell.

[10] Kawamata T, Tomari Y Making RISC.. Trends Biochem Sci.

[11] Ketting RF The many faces of RNAi.. Dev Cell.

[12] Fabian MR, Sonenberg N, Filipowicz W Regulation of mRNA translation and stability by microRNAs.. Annu Rev Biochem.

[13] Liu J, Carmell MA, Rivas FV, Marsden CG, Thomson JM (2004). Argonaute2 is the catalytic engine of mammalian RNAi.. Science.

[14] Meister G, Landthaler M, Patkaniowska A, Dorsett Y, Teng G (2004). Human Argonaute2 mediates RNA cleavage targeted by miRNAs and siRNAs.. Mol Cell.

[15] Schmitter D, Filkowski J, Sewer A, Pillai RS, Oakeley EJ (2006). Effects of Dicer and Argonaute down-regulation on mRNA levels in human HEK293 cells.. Nucleic Acids Res.

[16] Leung AK, Calabrese JM, Sharp PA (2006). Quantitative analysis of Argonaute protein reveals microRNA-dependent localization to stress granules.. Proc Natl Acad Sci U S A.

[17] Liu J, Valencia-Sanchez MA, Hannon GJ, Parker R (2005). MicroRNA-dependent localization of targeted mRNAs to mammalian P-bodies.. Nat Cell Biol.

[18] Hwang HW, Wentzel EA, Mendell JT (2007). A hexanucleotide element directs microRNA nuclear import.. Science.

[19] Liao JY, Ma LM, Guo YH, Zhang YC, Zhou H Deep sequencing of human nuclear and cytoplasmic small RNAs reveals an unexpectedly complex subcellular distribution of miRNAs and tRNA 3′ trailers.. PLoS One.

[20] Ohrt T, Mutze J, Staroske W, Weinmann L, Hock J (2008). Fluorescence correlation spectroscopy and fluorescence cross-correlation spectroscopy reveal the cytoplasmic origination of loaded nuclear RISC in vivo in human cells.. Nucleic Acids Res.

[21] Gibbings DJ, Ciaudo C, Erhardt M, Voinnet O (2009). Multivesicular bodies associate with components of miRNA effector complexes and modulate miRNA activity.. Nat Cell Biol.

[22] Lee YS, Pressman S, Andress AP, Kim K, White JL (2009). Silencing by small RNAs is linked to endosomal trafficking.. Nat Cell Biol.

[23] Kren BT, Wong PY, Sarver A, Zhang X, Zeng Y (2009). MicroRNAs identified in highly purified liver-derived mitochondria may play a role in apoptosis.. RNA Biol.

[24] Maniataki E, Mourelatos Z (2005). Human mitochondrial tRNAMet is exported to the cytoplasm and associates with the Argonaute 2 protein.. Rna.

[25] Hornig-Do HT, Gunther G, Bust M, Lehnartz P, Bosio A (2009). Isolation of functional pure mitochondria by superparamagnetic microbeads.. Anal Biochem.

[26] Cikaluk DE, Tahbaz N, Hendricks LC, DiMattia GE, Hansen D (1999). GERp95, a membrane-associated protein that belongs to a family of proteins involved in stem cell differentiation.. Mol Biol Cell.

[27] Jakymiw A, Lian S, Eystathioy T, Li S, Satoh M (2005). Disruption of GW bodies impairs mammalian RNA interference.. Nat Cell Biol.

[28] van Steensel B, van Binnendijk EP, Hornsby CD, van der Voort HT, Krozowski ZS (1996). Partial colocalization of glucocorticoid and mineralocorticoid receptors in discrete compartments in nuclei of rat hippocampus neurons.. J Cell Sci.

[29] Emanuelsson O, Brunak S, von Heijne G, Nielsen H (2007). Locating proteins in the cell using TargetP, SignalP and related tools.. Nat Protoc.

[30] Claros MG, Vincens P (1996). Computational method to predict mitochondrially imported proteins and their targeting sequences.. Eur J Biochem.

[31] Small I, Peeters N, Legeai F, Lurin C (2004). Predotar: A tool for rapidly screening proteomes for N-terminal targeting sequences.. Proteomics.

[32] Bhasin M, Raghava GP (2004). ESLpred: SVM-based method for subcellular localization of eukaryotic proteins using dipeptide composition and PSI-BLAST.. Nucleic Acids Res.

[33] Beitzinger M, Peters L, Zhu JY, Kremmer E, Meister G (2007). Identification of human microRNA targets from isolated argonaute protein complexes.. RNA Biol.

[34] Fleige S, Pfaffl MW (2006). RNA integrity and the effect on the real-time qRT-PCR performance.. Mol Aspects Med.

[35] Hausser J, Berninger P, Rodak C, Jantscher Y, Wirth S (2009). MirZ: an integrated microRNA expression atlas and target prediction resource.. Nucleic Acids Res.

[36] Ritchie W, Flamant S, Rasko JE mimiRNA: a microRNA expression profiler and classification resource designed to identify functional correlations between microRNAs and their targets.. Bioinformatics.

[37] Kawaji H, Nakamura M, Takahashi Y, Sandelin A, Katayama S (2008). Hidden layers of human small RNAs.. BMC Genomics.

[38] Pederson T Regulatory RNAs derived from transfer RNA?. Rna.

[39] Lu M, Zhang Q, Deng M, Miao J, Guo Y (2008). An analysis of human microRNA and disease associations.. PLoS One.

[40] Pagliarini DJ, Calvo SE, Chang B, Sheth SA, Vafai SB (2008). A mitochondrial protein compendium elucidates complex I disease biology.. Cell.

[41] Ekins S, Nikolsky Y, Bugrim A, Kirillov E, Nikolskaya T (2007). Pathway mapping tools for analysis of high content data.. Methods Mol Biol.

[42] Falkenberg M, Larsson NG, Gustafsson CM (2007). DNA replication and transcription in mammalian mitochondria.. Annu Rev Biochem.

[43] Baskerville S, Bartel DP (2005). Microarray profiling of microRNAs reveals frequent coexpression with neighboring miRNAs and host genes.. Rna.

[44] Politz JC, Hogan EM, Pederson T (2009). MicroRNAs with a nucleolar location.. Rna.

[45] Yang JH, Li JH, Shao P, Zhou H, Chen YQ starBase: a database for exploring microRNA-mRNA interaction maps from Argonaute CLIP-Seq and Degradome-Seq data.. Nucleic Acids Res.

[46] Willkomm DK, Hartmann RK (2006). Intricacies and surprises of nuclear-mitochondrial co-evolution.. Biochem J.

[47] Lynch M, Koskella B, Schaack S (2006). Mutation pressure and the evolution of organelle genomic architecture.. Science.

[48] Sen GL, Blau HM (2005). Argonaute 2/RISC resides in sites of mammalian mRNA decay known as cytoplasmic bodies.. Nat Cell Biol.

[49] Hock J, Meister G (2008). The Argonaute protein family.. Genome Biol.

[50] Chen CY, Zheng D, Xia Z, Shyu AB (2009). Ago-TNRC6 triggers microRNA-mediated decay by promoting two deadenylation steps.. Nat Struct Mol Biol.

[51] Janowski BA, Huffman KE, Schwartz JC, Ram R, Nordsell R (2006). Involvement of AGO1 and AGO2 in mammalian transcriptional silencing.. Nat Struct Mol Biol.

[52] Anderson S, Bankier AT, Barrell BG, de Bruijn MH, Coulson AR (1981). Sequence and organization of the human mitochondrial genome.. Nature.

[53] Hock J, Weinmann L, Ender C, Rudel S, Kremmer E (2007). Proteomic and functional analysis of Argonaute-containing mRNA-protein complexes in human cells.. EMBO Rep.

[54] Pare JM, Tahbaz N, Lopez-Orozco J, LaPointe P, Lasko P (2009). Hsp90 regulates the function of argonaute 2 and its recruitment to stress granules and P-bodies.. Mol Biol Cell.

[55] Griffiths-Jones S, Grocock RJ, van Dongen S, Bateman A, Enright AJ (2006). miRBase: microRNA sequences, targets and gene nomenclature.. Nucleic Acids Res.

[56] Kent WJ (2002). BLAT–the BLAST-like alignment tool.. Genome Res.

[57] Gasteiger EHC, Gattiker A, Duvaud S, Wilkins MR, Appel RD, Bairoch A, Walker JohnM (2005). Protein Identification and Analysis Tools on the ExPASy Server.. The Proteomics Protocols Handbook.

[58] Gaines G, Attardi G (1984). Highly efficient RNA-synthesizing system that uses isolated human mitochondria: new initiation events and in vivo-like processing patterns.. Mol Cell Biol.

[59] Asmann YW, Stump CS, Short KR, Coenen-Schimke JM, Guo Z (2006). Skeletal muscle mitochondrial functions, mitochondrial DNA copy numbers, and gene transcript profiles in type 2 diabetic and nondiabetic subjects at equal levels of low or high insulin and euglycemia.. Diabetes.

[60] Smith (1967). Preparation, properties and conditions for assay of mitochondria: Slaughter-house material, small scale. .. Methods in Enzymology.

[61] Olichon A, Emorine LJ, Descoins E, Pelloquin L, Brichese L (2002). The human dynamin-related protein OPA1 is anchored to the mitochondrial inner membrane facing the inter-membrane space.. FEBS Lett.

[62] Antonsson B, Montessuit S, Sanchez B, Martinou JC (2001). Bax is present as a high molecular weight oligomer/complex in the mitochondrial membrane of apoptotic cells.. J Biol Chem.

[63] Malka F, Aure K, Goffart S, Spelbrink JN, Rojo M (2007). The mitochondria of cultured mammalian cells: I. Analysis by immunofluorescence microscopy, histochemistry, subcellular fractionation, and cell fusion.. Methods Mol Biol.

[64] Rozen S, Skaletsky H (2000). Primer3 on the WWW for general users and for biologist programmers.. Methods Mol Biol.

[65] Sarkar M, Das S, Bandyopadhaya A, Ray K, Chaudhuri K (2005). Upregulation of human mitochondrial NADH dehydrogenase subunit 5 in intestinal epithelial cells is modulated by Vibrio cholerae pathogenesis.. FEBS Lett.

[66] Bolte S, Cordelieres FP (2006). A guided tour into subcellular colocalization analysis in light microscopy.. J Microsc.

[67] Adler J, Parmryd I (2007). Recent review on colocalization seem to misunderstand the Pearson correlation coefficient.. J Microsc.

[68] Zuker M (2003). Mfold web server for nucleic acid folding and hybridization prediction.. Nucleic Acids Res.

[69] Gruber AR, Bernhart SH, Hofacker IL, Washietl S (2008). Strategies for measuring evolutionary conservation of RNA secondary structures.. BMC Bioinformatics.

[70] Hofacker IL (2003). Vienna RNA secondary structure server.. Nucleic Acids Res.

[71] Zhang BH, Pan XP, Cox SB, Cobb GP, Anderson TA (2006). Evidence that miRNAs are different from other RNAs.. Cell Mol Life Sci.

[72] Wang X (2008). miRDB: a microRNA target prediction and functional annotation database with a wiki interface.. Rna.

[73] Huang HY, Chien CH, Jen KH, Huang HD (2006). RegRNA: an integrated web server for identifying regulatory RNA motifs and elements.. Nucleic Acids Res.

[74] Miranda KC, Huynh T, Tay Y, Ang YS, Tam WL (2006). A pattern-based method for the identification of MicroRNA binding sites and their corresponding heteroduplexes.. Cell.

[75] Tan MG, Ooi LL, Aw SE, Hui KM (2004). Cloning and identification of hepatocellular carcinoma down-regulated mitochondrial carrier protein, a novel liver-specific uncoupling protein.. J Biol Chem.

[76] Torchetti EM, Brizio C, Colella M, Galluccio M, Giancaspero TA Mitochondrial localization of human FAD synthetase isoform 1.. Mitochondrion.

