# Supplementary material for: Nuclear Outsourcing of RNA Interference Components to Human Mitochondria
Source: PLoS One. 2011 Jun 13;6(6):e20746. doi: 10.1371/journal.pone.0020746 (PMC3113838; doi:10.1371/journal.pone.0020746)
Supplement: Figure S1 — MiRNA profiling of HeLa mitochondria with or without RNase A treatment. (PDF) [file pone.0020746.s001.pdf]

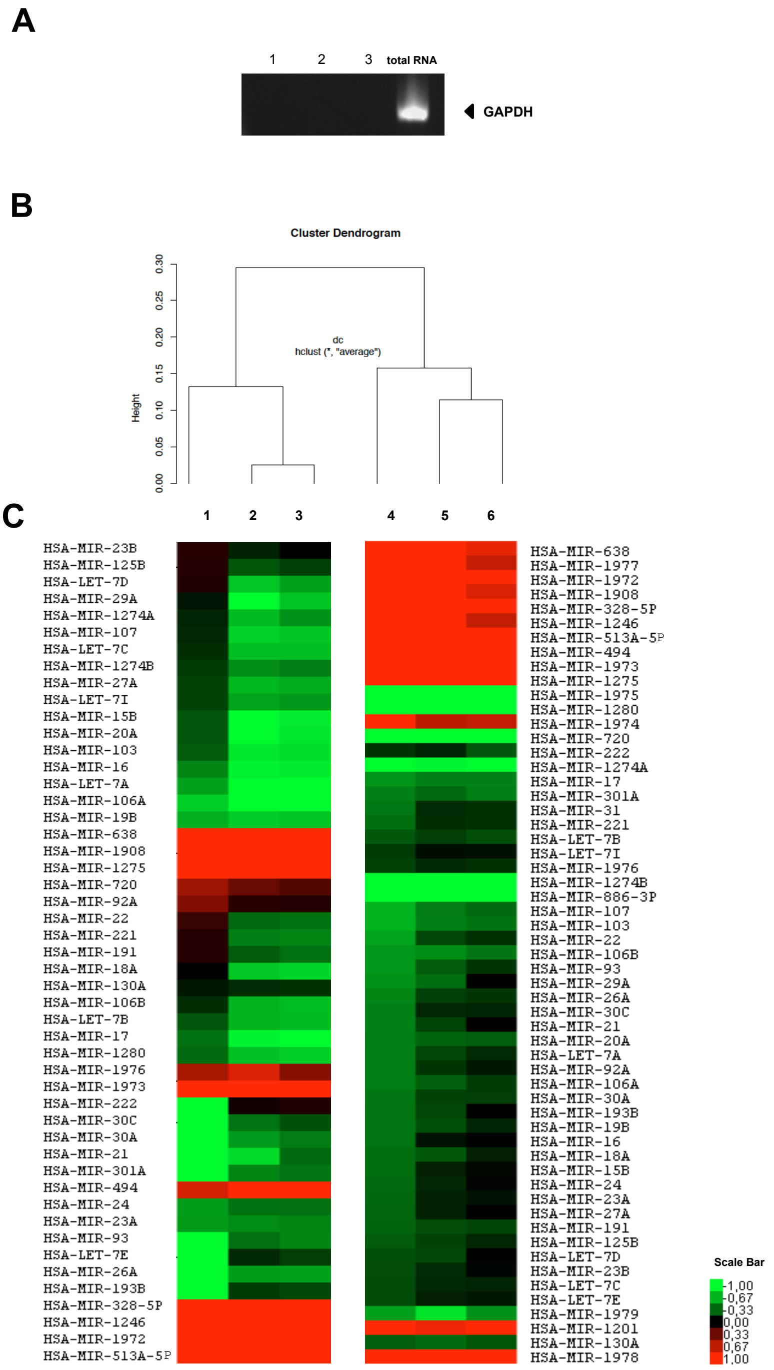

Figure S1: MiRNA profiling of HeLa mitochondria with or without RNase A treatment. A-C. HeLa mitochondrial fractions were treated with RNase A either for 15 min at 20°C (lanes 1) or for 30 min at 4°C (lanes 2 and 3). A. RT-PCR analysis of GAPDH in HeLa mitochondrial fractions. Total RNA is extracted from HeLa cells. B. Analysis of microarray data by hierarchical clustering in R software using Spearman correlation similarity measure and average. C. Heatmaps of microarray data with (left panel) or without (right panel) RNase A treatment. Each column (1 to 6) represents one independent mitochondria isolation experiment.
