# Supplementary figures and images for: Nuclear Outsourcing of RNA Interference Components to Human Mitochondria
Source: PLoS One. 2011 Jun 13;6(6):e20746. doi: 10.1371/journal.pone.0020746 (PMC3113838; doi:10.1371/journal.pone.0020746)

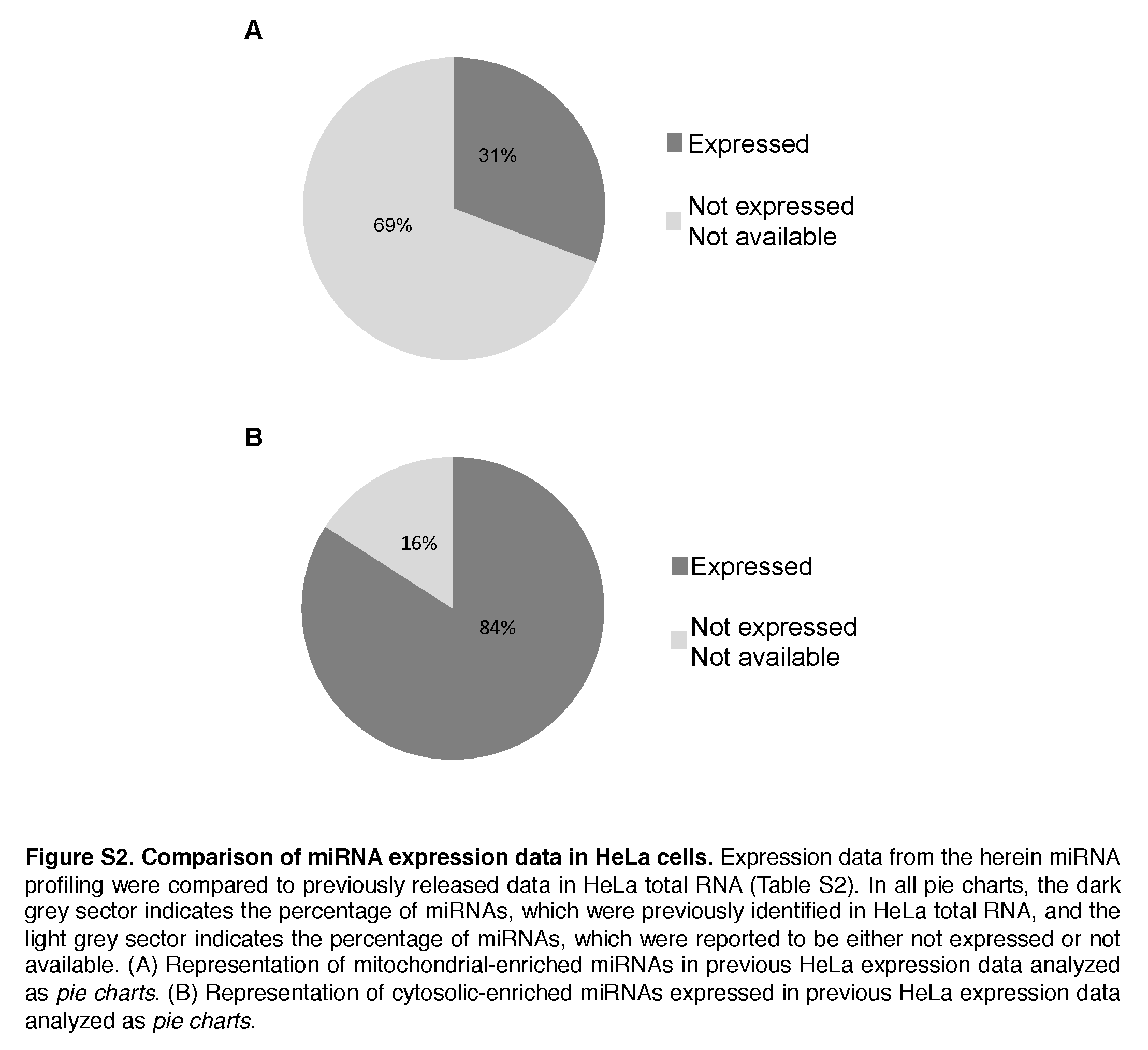

Supplement: Figure S2 — Comparison of miRNA expression data in HeLa cells. (TIFF) [file pone.0020746.s002.tiff]

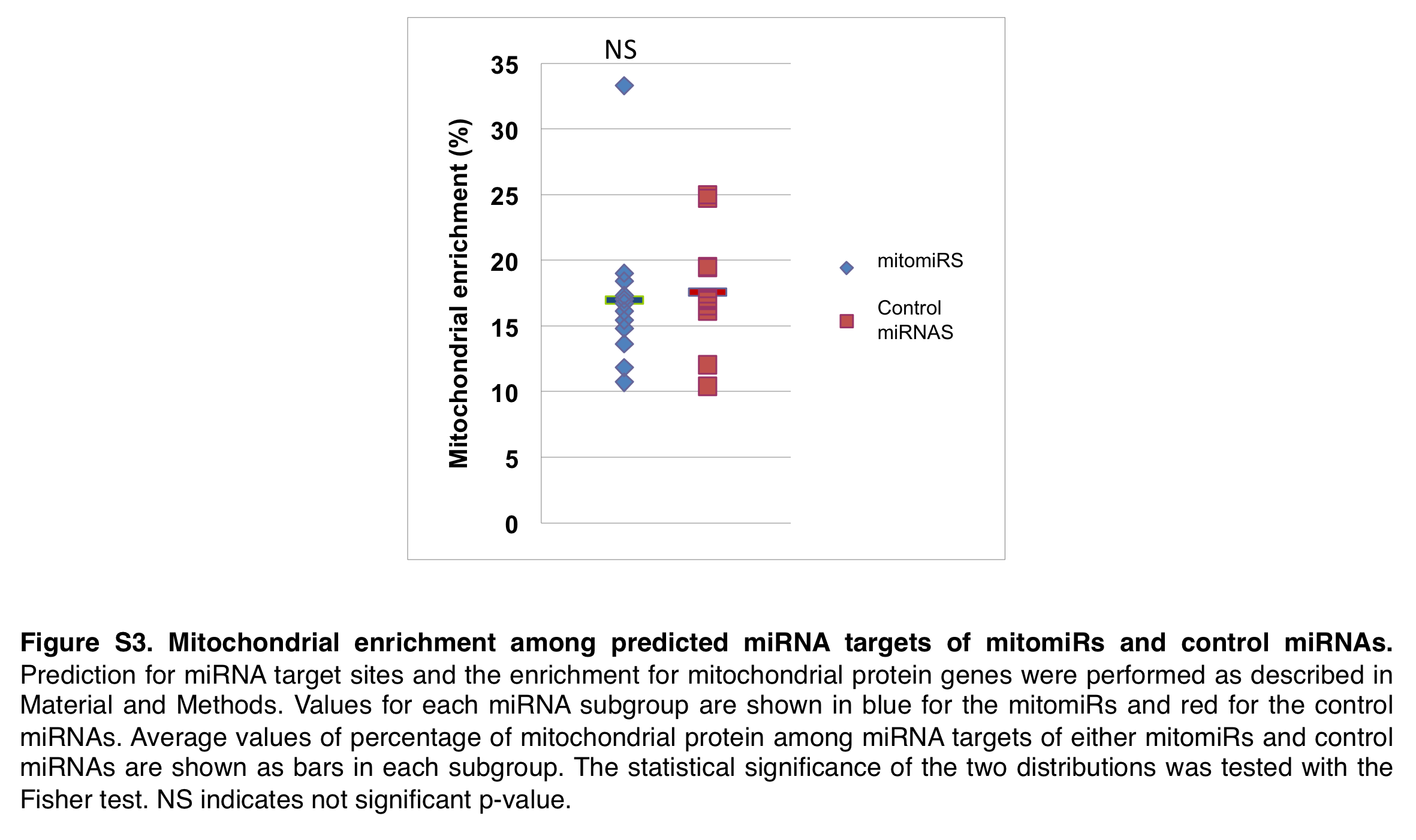

Supplement: Figure S3 — Mitochondrial enrichment among predicted miRNA targets of mitomiRs and control miRNAs. (TIFF) [file pone.0020746.s003.tiff]

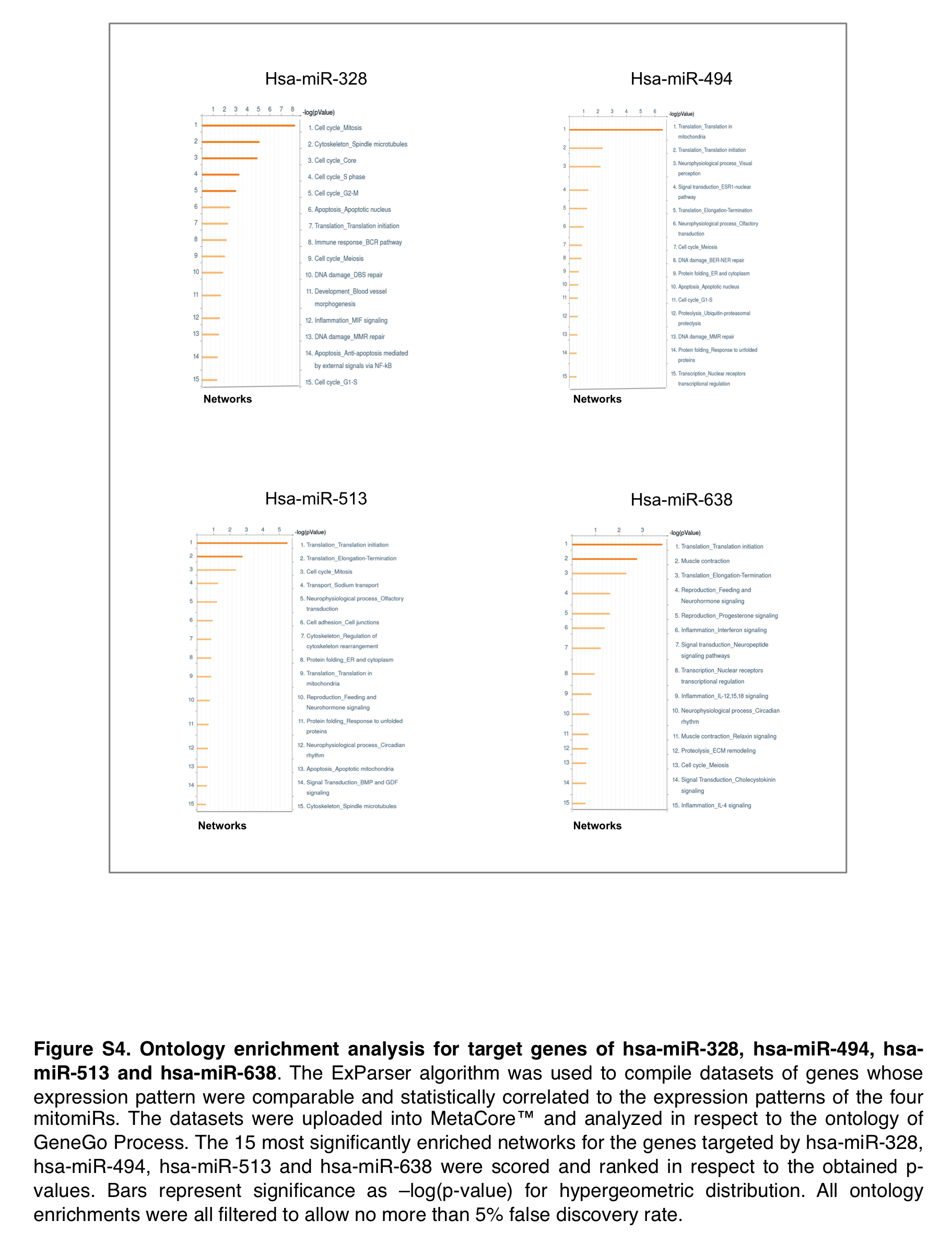

Supplement: Figure S4 — Ontology enrichment analysis for target genes of hsa-miR-328, hsa-miR-494, hsa-miR-513 and hsa-miR-638. (TIFF) [file pone.0020746.s004.tiff]
