# Supplementary material for: Nuclear Outsourcing of RNA Interference Components to Human Mitochondria
Source: PLoS One. 2011 Jun 13;6(6):e20746. doi: 10.1371/journal.pone.0020746 (PMC3113838; doi:10.1371/journal.pone.0020746)
Supplement: Supporting Information S1 — Measurement of enzymatic activities in mitochondrial fraction. (DOC) [file pone.0020746.s005.doc]

**Supporting Information S1- Measurement of enzymatic activities in mitochondrial fraction.**

The efficiency of the isolation procedure is determined by measuring the mitochondrial marker enzyme citrate synthase and the cytosol-specific marker enzyme lactate dehydrogenase remaining in the mitochondrial fraction following the extraction procedure. Citrate synthase activity is measured spectrophotometrically based on the absorption at 412 nm by the product thionitrobenzoic acid (TNB), which, in the presence of saturating concentrations of substrates acetyl-CoA, oxaloacetate, and dithionitrobenzoic acid (DNTB), is a function of the activity of citrate synthase [1]. Lactate dehydrogenase activity is based on conversion of pyruvate to lactate and simultaneous oxidation of NADH to NAD. The rate of decrease in NADH is directly proportional to the LDH activity and is determined spectrophotometrically at 340 nm. The percentage of residual LDH represents the fraction of LDH activity found in the mitochondria-enriched fraction with respect to the enzyme activity in initial homogenate. We found that only 0.16+0.03 % of the homogenate lactate dehydrogenase activity is found in the mitochondria-enriched fraction, indicating a very low cytoplasmic contamination of the final mitochondrial preparation. There is also a 3-fold enrichment in citrate synthase. The extraction yield is determined by expressing citrate synthase measured in the mitochondrial fraction as a percentage of the activity in the initial cell suspension homogenate [2,3]. This extraction method results in yields of 19.1+3%. Furthermore, to check for the integrity of isolated mitochondria, function of our mitochondrial preparation was assessed by measuring citrate synthase activity in isolated mitochondria before and after membrane disruption by extraction of enzyme by Triton X-100 [4]. Citrate synthase activity was measured using standard spectrophotometric techniques, as described above. We found an integrity range of our mitochondrial preparations of 95.4 ± 3.1%.

**References**

1. Srere PA (1969) Citrate synthase. Methods Enzymol 13: 3-5.

2. Rasmussen HN, Rasmussen UF (1997) Small scale preparation of skeletal muscle mitochondria, criteria of integrity, and assays with reference to tissue function. Mol Cell Biochem 174: 55-60.

3. Rasmussen HN, Andersen AJ, Rasmussen UF (1997) Optimization of preparation of mitochondria from 25-100 mg skeletal muscle. Anal Biochem 252: 153-159.

4. Asmann YW, Stump CS, Short KR, Coenen-Schimke JM, Guo Z, et al. (2006) Skeletal muscle mitochondrial functions, mitochondrial DNA copy numbers, and gene transcript profiles in type 2 diabetic and nondiabetic subjects at equal levels of low or high insulin and euglycemia. Diabetes 55: 3309-3319.
