# Supplementary material for: Nuclear Outsourcing of RNA Interference Components to Human Mitochondria
Source: PLoS One. 2011 Jun 13;6(6):e20746. doi: 10.1371/journal.pone.0020746 (PMC3113838; doi:10.1371/journal.pone.0020746)
Supplement: Table S1 — Prediction of subcellular localizations of AGO2. (DOC) [file pone.0020746.s006.doc]

**Supporting Information**

**Table S1: Prediction of subcellular localization of AGO2**

| **AGO2 isoform (NCBI gene identity GI)** | **TargetP 1.1** | | |
| --- | --- | --- | --- |
| **Sequence** | **Score for a mitochondrial target peptide** | **Predicted subcellular localiation** | **Predicted pre-sequence length (aminoacid residues)** |
| Isoform CRA_b (GI:119612614) | 0.79 | Mitochondria | 9 |
| Isoform 1 (GI:29171734) | 0.20 | none | 0 |
| Isoform 2 (GI:257467482) | 0.20 | none | 0 |
| EIF2C2 (GI:133777965) | 0.75 | Mitochondria | 12 |

| **AGO2 isoform (NCBI gene identity GI)** | **MitoProt II v1.101** | |
| --- | --- | --- |
| **Sequence** | **Probability for a mitochondrial targeting peptide** | **Predicted mitochondrial targeting peptide length (amino acid residues)** |
| Isoform CRA_b (GI:119612614) | 0.95 | 24 |
| Isoform 1 (GI:29171734) | 0.07 | Not predictable |
| Isoform 2 (GI:257467482) | 0.07 | Not predictable |
| EIF2C2 (GI:133777965) | n.a. | n.a. |

n.a. indicates that prediction was not available

| **AGO2 isoform (NCBI gene identity GI)** | **Predotar 1.03** | |
| --- | --- | --- |
| **Sequence** | **Probability for a mitochondrial targeting peptide** | **Predicted subcellular localization** |
| Isoform CRA_b (GI:119612614) | 0.21 | Possibly mitochondrial |
| Isoform 1 (GI:29171734) | 0 | none |
| Isoform 2 (GI:257467482) | 0 | none |
| EIF2C2 (GI:133777965) | n.a. | n.a. |

n.a. indicates that prediction was not available

| **AGO2 isoform (NCBI gene identity GI)** | **ESLPred** | |
| --- | --- | --- |
| **Sequence** | **Predicted subcellular localization** | **Expected accuracy** |
| Isoform CRA_b (GI:119612614) | Mitochondria | 94% |
| Isoform 1 (GI:29171734) | Mitochondria | 94% |
| Isoform 2 (GI:257467482) | Mitochondria | 94% |
| EIF2C2 (GI:133777965) | Mitochondria | 94% |
