# Supplementary material for: Nuclear Outsourcing of RNA Interference Components to Human Mitochondria
Source: PLoS One. 2011 Jun 13;6(6):e20746. doi: 10.1371/journal.pone.0020746 (PMC3113838; doi:10.1371/journal.pone.0020746)
Supplement: Table S2 — Compiled miRNA expression profiling data in HeLa cells. (DOC) [file pone.0020746.s007.doc]

**Supporting information**

**Table S2: Compiled miRNA expression profiling data in HeLa cells**

| **miRNA identity** | **MirZ** | **mimiRNA** | **Current profiling data** |
| --- | --- | --- | --- |
| hsa-let-7a | + | + | C |
| hsa-let-7b | + | + | C |
| hsa-let-7c | + | + | C |
| hsa-let-7d | + | + | C |
| hsa-let-7e | n.a. | + | C |
| hsa-let-7i | + | + | C |
| hsa-miR-103 | n.a. | + | C |
| hsa-miR-106a | n.a. | + | C |
| hsa-miR-106b | + | + | C |
| hsa-miR-107 | n.a. | + | C |
| hsa-miR-1201 | - | n.a. | M |
| hsa-miR-1246 | - | n.a. | M |
| hsa-miR-125b | + | + | C |
| hsa-miR-1274a | - | n.a. | C |
| hsa-miR-1274b | - | n.a. | C |
| hsa-miR-1275 | - | n.a. | M |
| hsa-miR-1280 | - | n.a. | C |
| hsa-miR-130a | n.a. | + | C |
| hsa-miR-15b | + | + | C |
| hsa-miR-16 | + | + | C |
| hsa-miR-17 | + | + | C |
| hsa-miR-18a | n.a. | + | C |
| hsa-miR-1908 | - | n.a. | M |
| hsa-miR-191 | + | + | C |
| hsa-miR-193b | + | + | C |
| hsa-miR-1972 | n.a. | n.a. | M |
| hsa-miR-1973 | n.a. | n.a. | M |
| hsa-miR-1974 | n.a. | n.a. | M |
| hsa-miR-1975 | n.a. | n.a. | C |
| hsa-miR-1976 | n.a. | n.a. | C |
| hsa-miR-1977 | n.a. | n.a. | M |
| hsa-miR-1978 | n.a. | n.a. | M |
| hsa-miR-1979 | n.a. | n.a. | C |
| hsa-miR-19b | + | + | C |
| hsa-miR-20a | + | + | C |
| hsa-miR-21 | + | + | C |
| hsa-miR-221 | n.a. | + | C |
| hsa-miR-222 | + | + | C |
| hsa-miR-23a | + | + | C |
| hsa-miR-24 | + | + | C |
| hsa-miR-26a | + | + | C |
| hsa-miR-27a | + | + | C |
| hsa-miR-29a | + | + | C |
| hsa-miR-301a | + | + | C |
| hsa-miR-30a | + | + | C |
| hsa-miR-30c | + | + | C |
| hsa-miR-31 | + | + | C |
| hsa-miR-328 | - | + | M |
| hsa-miR-494 | - | + | M |
| hsa-miR-513a | - | + | M |
| hsa-miR-638 | - | + | M |
| hsa-miR-720 | - | n.a. | C |
| hsa-miR-886-3p | + | + | C |
| hsa-miR-92a | + | + | C |
| hsa-miR-93 | + | + | C |

Data were compiled from MirZ (Hausser et al, 2009), mimiRNA (Ritchie et al, 2010) and our current data. Expression of miRNA in HeLa cells is indicated as + , conversely as – when not expressed and n.a. when not available. M and C respectively represent the enrichment in mitochondria and cytosolic HeLa fractions from our current data.
