# Supplementary material for: Nuclear Outsourcing of RNA Interference Components to Human Mitochondria
Source: PLoS One. 2011 Jun 13;6(6):e20746. doi: 10.1371/journal.pone.0020746 (PMC3113838; doi:10.1371/journal.pone.0020746)
Supplement: Table S3 — Nuclear-encoded computational target genes of mitomiRs and control miRNAs. (DOC) [file pone.0020746.s008.doc]

**Supporting information**

**Table S3: Nuclear-encoded computational target genes of mitomiRs and control miRNAs**

| **miRNA identifier** | **Computational targets** |
| --- | --- |
| **hsa-miR-1973** | CDK2AP1, C14orf4, MAFF, KCNJ15, GDAP1, MBNL2, LRRK2, CAPN3, TMEM80, GZF1, BCAS1, GOT1, CACNB1, KPNB1, TBX18, AZIN1, GABRP, TBX15, IL8, CTAGE5, CEP68 |
| **hsa-miR-1275** | ZBTB7A, SLC8A2, RP11-251J8.3, CXADR, septin6, FOXA3, USP41, SH2D4A, JMJD5, SPARC, SCUBE3, SLC36A1, LY6E, TTYH2, LHX6, NKAIN4 |
| **hsa-miR-494** | NOVA1, SLC1A2, FAM169A, ARHGAP5, ERCC4, MAP4, C10orf141, TLR6, CNR1, PITX2, RBM4B, MFAP3L, AKAP8, ZNF81, EVI5, UNC45B, ZXDB, LOC100129165, TFPI, ANKRD50, ZC3H6, BMPR2, VGLL3, PROS1, AKAP7, C13orf18, RDH10, APPL1, SHROOM3, SEMA3A, ENAH, CXCL5, SOCS6, IKBKAP, ANKRD22, C11orf30, GABRA1, KLF11, PTEN, CFTR, WIPF1, ZNF543, BTN2A2, EVI2B, CSNK1G1, MYO6, DZIP3, FGFR2, septin9, B3GALT2, LRRC19, ZFHX3, NR2C1, ZNF25, MYLIP, NAV3, ZBTB8, YAF2, SRFBP1, VCPIP1, DPYD, BCL2L15, SHOC2, RFX7, CGGBP1, PRTG, LARP2, LRAT, BACH1, PITPNB, WDR26, SCD5, SLC26A3, CRKRS, CMPK2, ROCK1, FBXO34, SLC6A15, RB1CC1, KIAA0895, CAPS2, CUL4A, NAALADL2, TNKS, ACSL4, ANKS1B, DCP2, ATXN3, DSG2, MTUS1, PRKAB2, C2orf21, ACVR1C, PCDH19, 7A5, KIF3A, GRAMD1C, JAKMIP2, PDE4B, DDEF2, MYST3, NAB1, ATP8A1, JMJD2C, PCLO, NMNAT2, PBRM1, TMEM135, DNAJA2, HNMT, NAP1L2, LOC652725, MGA, GALR1, TET1, NADK, USP31, TCEA1, SCAMP1, FER1L3, COL6A6, SLC39A6, ARHGAP11A, PDS5B, HS2ST1, VPS13A, CAMK1, ZNF233, WDR89, DNAJC6, DCP1B, USP47, PDCL3, ZKSCAN2, CCT5, KIAA1147, NBEA, FGL2, DNHL1, C1QTNF7, EXOC8, C11orf54, SLC7A11, TACC1, GALNT7, LOC642558, EIF5, TMTC3, ZC3H12C, TMEM154, GULP1, SLC30A10, RNF138, RTKN2, INSC, HADH, LGR4, ZNF654, OSBPL6, TBC1D3G, LEFTY2, PIGA, AGPS, TMEM87A, POLR3G, SFRS7, SFRS17A, ATP8A2, NHLH2, ZIC2, TRDMT1, GCC2, EIF2C4, C6orf151, SENP3, FBXO9, KIAA0776, PDIK1L, ANXA11, FAM111A, PTGER3, KIF4A, C1orf26, H2AFB3, ZRANB3, GIN1, FAM155A, ENPP1, USP48, LOC647286, TDP1, PGAP1, LOC100133836, UBP1, ELF1, FBXW11, ARHGAP29, TWSG1, SERPINE1, SMN1, SEC24B, ARHGEF17, ARHGAP28, LOC100133835, HEPACAM2, MEGF9, C1orf198, SMN2, RAPGEF5, RPP30, GLCE, ZFP62, UBE2L6, SERPINB9, TSHR, LOC653382, ARFGAP3, RAI14, TMED2, COL4A5, CPT1A, SYPL1, SNAI2, LYSMD3, C15orf29, TRPS1, FGF14, LUZP2, ABCG5, GALNT1, PDLIM4, GPLD1, PPFIA1, HEATR5B, RWDD4A, TACC2, C1orf149, FNBP1L, FAM159B, UACA, ZNF264, HOXA10, PLSCR4, H2AFV, INTS2, BBS7, CEP120, ZNF705A, PPP2R3A, TNRC18, C11orf41, LOC145814, DBX2, PCOLCE, ZNF705G, GLI3, C9orf150, CSGALNACT2, NNT, C14orf82, ZNF705D, FLJ42562, TCP10L, USP6, LOC100134348, CTR9, AK5, LOC100131539, TLE1, SHISA2, HDAC9, USP32, CLPTM1L, POU3F2, SMURF2, ENTPD1, CCNT2, CLEC4D, PLEKHG1, MORC3, SFRS2IP, KHDRBS1, IGF2BP1, PHF16, GABRA5, SPATA16, PPM1D, IL1RAP, RGPD5, RGPD8, API5, SH3PXD2A, TBC1D3C, TBC1D3F, RFWD3, LOC729837, GPR158, LOC653380, TBC1D3E, ZFX, LOC653498, BCLAF1, A1CF, MED21, TBC1D3H, GPNMB, RGPD6, ZNF767, RGPD4, RGPD3, KLRC4, SLAIN2, RAP2C, PI15, C6orf91, USO1, ZFHX4, WDR90, LMOD3, CASP2, ZNF207, CYP26B1, FMO5, SLC30A4, SLC25A16, IL1R1, LIPH, LBA1, LOC282992, LOC653550, UTP15, ZBTB41, LOC729264, CHD9, STAT1, EIF2AK2, SLC35F5, S100A1L, CAND2, BOLL, NAAA, SERPINB4, HS3ST3A1, LOC643475, RHOXF2B, SNX9, SERPINB3, MDFIC, ZC3H7A, KCTD10, RAD51, CD28, RHOXF2, LOC100130053, ARID4B, BCAP29, C11orf61, HMGCS1, RRS1, LOC729240, IL26, WDR33, ADAMTSL3, YTHDF2, FLJ40296, TGIF1, BCOR, LOC729250, EDN1, LOC729246, DUOX2, ZBTB44, DSCC1, GK, ZCCHC8, MORF4L2, EIF2C3, NFKBIZ, KIAA1244, FAM3C, HMP19, LOC729233, GANC, ZNF621, ZNF750, TMEM26, SMARCE1, PLS1, BHLHB2, C5orf39, NLGN4Y, POM121, MAGEE1, TMEM90A, POM121C, KLHDC10, PTER, ZC3H12B, LOC100133431, SLC5A8, SAP18, REEP1, ZNF396, TBC1D14, KPNA1, FAM35A, SOCS5, GALNT2, NXPH1, ELMOD1, PSG2, FAM102B, FZD6, PTPN12, PPP1CB, SLC35A5, WDR44, UBASH3B, EML4, PPM1A, TXNL1, KIAA1632, H3F3B, CDH17, CAV2, FOXL1, ARHGEF10, PHYHIPL, TAOK3, SLC46A3, HAS2, MUT, HNRNPA3, RNF8, LONRF2, LOC552891, STAM, MTMR9, PPWD1, WWC3, PRDM4, SMAD1, TEX15, POLR3F, IL6ST, ENPEP, NPNT, LRFN5, PCDHB10, CCDC59, NPHS2, GNG10, WDR45L, ZSCAN23, BCHE, LYCAT, HIST1H2AG, CSMD3, FAM91A1, ARNTL, SCARB2, TARSL2, B4GALT6, MRPL43, SH3BP2, KMO, FSTL5, AFF1, MBNL2, LOC100128191, RAB40B, G3BP1, PTPRB, ADRA2A, TUBA1C, DSC3, UBE2J1, SEMA5A, SPSB4, CHCHD8, SEH1L, ARPM1, GFPT1, STON1, GAGE1, RAP1B, GPRC5B, UBQLN2, EPHB1, POLS, tcag7.1228, CH25H, RBM39, DSC1, RICH2, FIGF, ANKHD1, GGNBP2, WDR37, SOX1, ZBTB39, IL17RD, SFXN1, AOF1, PRDM6, CRIPT, CCNYL1, PXMP4, SOCS4, C1orf144, CCDC141, KLHL7, MLLT4, TOR1AIP1, MAP3K7IP3, ZNRF2, GRIK2, KIAA1549, LATS2, SLC7A2, LOC100133914, ALG8, MAP4K4, PPP1R12B, TEAD1, ELF2, HLA-DQA1, CDK6, TCEA2, NAALAD2, STARD3NL, BMP2K, DERL1, LMOD2, CD5L, RNF103, FREM2, LOC161527, LRRIQ3, NEDD4, FOXJ3, PUS7L, MAGEB4, ELOVL5, BMPER, PHEX, ZNF711, SH3PXD2B, ZNF12, MFN1, UGT2A3, STOML3, TAPBP, RAB5A, MICAL1, DENND5B, PFDN4, ABCC12, GUCA1B, KCTD14, UGT2B17, CNOT6L, IL16, LOC100129095, SEC22B, TMPO, IL12B, RERE, LOC283788, HIVEP1, CTBS, ABCA1, ZNF460, PDP2, LOC727944, ABCC5, FAM29A, C18orf54, OSGEPL1, PAK7, IFI44L, GPR26, COPB1, SLC5A7, CYB5A, ANP32E, TMEM19, ZNF257, MMD, ZNF281, C5orf32, PTPRK, C16orf57, ALG10B, LOC100132735, C12orf66, PGR, DDX17, SFRS2, MSL3L1, SDK1, GCLC, CCDC108, LOC100134141, WDFY1, CHCHD3, IL1A, FAM116A, UNC84A, SLFN11, CNIH, TTC14, ANKFY1, LOC100132892, TXNDC8, LOC285620, MS4A6A, SLC38A1, RASA3, TBC1D2B, LOC729272, LOC100133649, USP15, PHF8, GRPEL2, RET, PAK1, FLJ36131, ASF1A, PRKAA2, SUSD1, ZPLD1, SCFD1, SRP54, GLDN, C21orf63, GLIS3, PTGS2, GPR85, PSEN1, EN2, LOC645202, LOC440243, LOC283767, PDCL, PECAM1, JUND, RFK, DBN1, LOC727942, PHACTR2, PCSK1, TAC1, PMAIP1, MYC, JMJD1B, FAM120A, PLEKHA2, TRAPPC10, NAMPT, IKBKB, SPCS1, SKIL, LOC644914, PAP2D, TOX4, LHFPL3, MXD1, DCN, CCDC4, POF1B, RB1, PPP1R8, PIGK, TMEM187, MNDA, KLHDC6, CAMTA1, ANKRD6, AAK1, SH3RF3, STX6, KLHL13, TLK2, RAD51L3, F5, STK3, KIAA1045, ELOVL3, C10orf28, KIAA0515, MLL, ST8SIA2, WDR1, RAPH1, C17orf100, LYSMD4, FAM136A, WDR20, MBD5, GPR103, ZNF623, ZNF425, UPF2, ISCA1, FBXO48, UBQLNL, CREM, LAPTM4A, LCORL, ERLIN2, BBS10, PURB, LOC100130886, LOC644538, MSI1, HOXD13, LOC729355, SLC38A9, USP36, CFHR5, LOC100130890, C5orf24, RRP15, LOC100133862, RAD23B, TP53TG3, KBTBD3, C8orf37, WHSC1, RAB12, TGOLN2, FLJ42177, NUTF2, TXNDC10, CMTM4, WDR35, ALDH1B1, KIAA1715, UNC5D, ZMAT1, septin8, RAB11FIP2, LOC100134367, LOC100134211, BRMS1L, APOBEC4, PCDHB9, RALYL, SEC62, SHISA3, SKAP2, KIF2C, HEATR3, ETV3, C14orf4, CYP2J2, SPTLC3, LOC647795, TRAPPC2, EIF1B, CDS1, ARHGAP20, TNFSF10, SRGAP1, LRRFIP2, CUGBP2, MAP2K1, MYNN, C1orf55, SETD7, POLQ, PRPF4B, C3orf64, YWHAE, TBL1X, IGF1R, ADRBK2, GLT6D1 |
| **hsa-miR-513a-5p** | CSNK1G1, GNG13, PNPLA7, FBXO34, ZNF329, CCDC52, GLT8D3, C12orf4, SLC1A2, KIAA2018, KLF3, TNPO1, LOC100134785, XDH, ZNF346, CUBN, SH3TC2, GPR39, HRB, EPS8, CREB3L2, VPS53, ZFP41, CLK2, ADAM9, C3orf34, UNC5D, ANO6, MORC3, LOC100130216, VPS35, USP9Y, RGS5, LOC100133609, DCTN4, tcag7.1228, ENC1, ATAD2B, KRAS, RXRB, SNN, C1orf173, MCHR2, BAALC, DENND5B, CCRL1, C10orf25, ARFGEF1, ZNF84, SRRM2, PPP1R9A, NAPB, SNX10, BTG1, AQP4, LIN7C, NEO1, PDZD11, ZNF44, PPARGC1A, SLC5A3, BNIP3L, TRIM44, CTBP2, ELAVL2, GRSF1, DSP, ZNF514, ELOVL5, XRCC6BP1, FN1, QKI, AP1GBP1, RCAN1, TNRC6B, ZNF200, SBF2, FAM35A, RAPGEF5, TLR7, PRKCSH, MKLN1, PHF19, SERP1, ATP6V1G2, MPPED2, C14orf108, PURA, GCET2, SCN1A, CAGE1, ADAMTSL3, CENTB2, CRIPAK, USP47, NEBL, KRR1, LYRM7, KIAA0586, ZNF599, YTHDF3, CYTIP, CNTNAP2, KLC1, CNR1, MAL, UHRF1BP1, CHST6, LOC100133876, MGAT4A, SLC26A9, LOC100131686, C14orf147, KLHL12, LOC100133607, DDAH1, PFTK1, SLC6A3, TP53INP1, SAPS3, LONRF2, KIAA1328, TRIM10, ZBTB1, METTL10, ARMC4, BTBD11, CA13, RGS16, PXN, NR2C2, ADD3, MT1F, CCDC82, AGPS, MAGI2, USP31, GTF2I, C16orf55, TMEM173, ERAP1, LAMB4, MSL2L1, SURF6, PCP4, SCML4, DTNA, ZNF197, CTNND1, TMEM50B, VSX2, SYN2, PPAP2B, BHLHB5, TRAPPC10, CYP39A1, DCP2, PAP2D, ADH1B, STAT1, POLI, THUMPD1, ARHGEF9, NKIRAS1, LIFR, MTF1, SARM1, LOC100131180, LCORL, NDUFS1, SEMA4F, QRSL1, TBC1D9B, HSDL1, PLCXD3, LOC100134022, NCALD, SLC16A12, MRE11A, GRB10, KCNB2, ASF1A, LOC100133205, EI24, ZNF665, CSNK1A1L, RBPMS2, PDXK, PEX5, JARID1A, AP2B1, FIZ1, RBM12B, KAT2B, ZNF562, HHLA3, SMAD2, SMC1A, CHD1, PARP12, TRIM2, UGT2B15, EPS15, UBXN7, BTN1A1, SOD1, ALDH5A1, LOC728160, EYA1, MAP2, DNAJA2, BDKRB2, PDGFD, RAC2, CBLB, ZNF257, SMCR8, C16orf61, SMAD4, C10orf105, MID1, CRK, ALG9, GPX7, ZNF440, TMEM66, TPR, GALR1, IMPA2, DNASE2B, TRPM3, DCX, LOC100130890, TEAD2, DGKB, DISC1, MRPL14, SH3BP2, UBE2K, CYP1B1, KBTBD4, IKZF2, LOC100134259, CYP8B1, C8orf40, FBXO31, SLC30A8, HHIP, AK2, PTER, C11orf30, RORB, CDC40, STX5, FLJ35379, EVI2A, C12orf69, WDR33, ARHGEF12, TLE4, ZDHHC17, KAL1, KLHL4, LOC727942, PSRC1, SH3GL3, LOC646509, ZNF189, CTSO, MALT1, SCN9A, COL5A3, NIPA2, LOC100128403, FAM184A, JPH1, HCG27, C9orf152, LOC728320, DCUN1D4, USP4, WDR43, ACOX3, CAMK2D, ZNF624, SFMBT2, SSBP2, C1orf21, GRPR, PET112L, HEMK1, SNTA1, MAGOHB, MAPK14, MPPE1, FAM53A, STX17, EDIL3, ZC3H4, EARS2, ZNF275, MRO, KCNMB2, KRTAP6-3, ARFGAP3, septin14, ABCA9, RAB40C, TBR1, LOC100133749, MITF, ZC3H12B, INO80D, SNX30, LOC732437, CDK6, CCDC65, HIPK2, UGT2B28, NEU1, UBE2A, EVI5, ANKRD34B, FAM123B, DDT, ST8SIA1, TXNIP, LOC100132436, TOX4, LOC100133711, GRM8, ZDHHC7, FGL2, FAM105B, GNE, SYT1, MAP3K12, LATS2, KCTD14, NODAL, MLPH, NAPEPLD, PDLIM5, DLGAP2, GMFB, TTR, TSHR, ZNF415, INHBE, PIP4K2B, LOC730011, ZNF609, LOC100129858, UBA5, ERLIN2, LOC730236, ONECUT2, POLD3, LOC100130955, KL, MIER3, KIAA0802, OTUD4, PTPRN2, ZRANB2 |
| **hsa-miR-1246** | GSG1L, SGOL1, KLRA1, RTKN2, MSR1, FAM53C, CREBL2, SLC16A7, BRWD1, SMG7, SEMA6A, ZNF267, GLRB, C6orf168, TMEM123, SCN3A, CCDC4, MUT, CLCC1, MYOT, SEPHS1, AGL, PRKAA2, PCSK6, TMEM184A, ATF1, KLHL14, DYNC1I1, DNAJB14, GPR161, SH3TC2, MCCC2, LOC730432, FAM45A, ZNF518B, ERBB4, C12orf71, PSD3, DYRK1A, LOC100132163, METAP2, MALT1, JARID1A, ZNF502, C3orf43, PIP4K2A, ANGEL2, SGK269, PRO0628, MSN, PHLDA1, TPRG1L, FAM153B, SEC63, CMTM6, BACH1, SYT14, ZNF566, GPR85, LOC727942, LY75, LOC100129413, AASDHPPT, HELZ, NCAPH, DMRT2, ARSJ, ZNF714, UBE2A, SORL1, ZCCHC6, YAF2, WDR77, SOCS4, FAM19A5, ZNF124, DUSP18, CLIP4, PROM1, IDS, SLC38A2, PTPLAD2, DLG1, DDI1, SLC25A27, NLRP3, IYD, C10orf30, KIAA1370, KCNS3, SLC35C1, VIM, C5orf36, KIAA0776, FAM84A, GNRHR, ADAMTS6, TSPYL4, CDH2, CAMK2G, FLJ20674, LHFPL3, STXBP5, RAD50, PTAR1, BAHD1, CHKA, DTL, LOC100132051, MITF, CD22, ACVR2A, CDO1, THRB, E2F8, TSPAN31, LOC26010, NDFIP1, IHPK1, CTSC, FRMD6, REPS2, FMNL3, C18orf34, RLBP1L1, EDA, GOLGA1, RRN3, AXIN2, SCML2, GRHL1, PRELP, RGMB, C20orf26, LIN54, LOC728849, UBE2G1, USP54, HCCS, SPAM1, ATRX, PMP2, PLEKHB2, FAM151B, KIAA0895, ACBD3, HECTD2, GRM1, SYT16, MGC3207, ARFIP1, CLEC6A, VPS53, ANKRD42, GPR26, LAX1, FSTL5, MIER1, ZNF425, KIAA0355, MIER3, KIAA1715, C16orf45, NIPBL, PAIP2B, KLC1, GRHL2, AP1S3, TAOK1, INPP4B, ESR2, BCL2L2, C10orf90, ZNF770, KCNAB1, EPC2, VCPIP1, GAPVD1, AFF3, LOC153469, C9, ZNF845, C21orf45, ALG10, SLAIN1, KIAA1377, SOX2, FAM98A, ZNF697, ANKFY1, SCN1A, ROPN1, DERA, IQCA1, NPAT, CLEC4C, CHST9, FGL2, SLC43A2, DCP2, XK, TET1, EHF, RIMBP2, ACADL, PEG3, PAPD1, PHYHIPL, GLIPR1, LOC100134793, AKAP9, TAF9B, QKI, LOC391343, LOC100130332, FGF5, ZIC3, IRAK2, FLJ44054, LOC286177, CISD1, DSCC1, EXOSC9, CALM2, ARL4C, FNDC3A, FLJ41481, CDC25A, FIGF, HHAT, ZNF254, FERMT2, KITLG, LRRTM2, UBXN2B, C16orf63, PLEKHG2, TNFRSF8, PCM1, TAP2, PML, C4orf40, TUBGCP3, SPCS2, RP5-1000E10.4, NDUFA5, ERCC4, CCNG2, RPP30, ADAT2, TRDMT1, CCDC88A, ZAK, ZNF470, GDAP1, MRPL19, RNF125, FLJ35220, GIGYF2, PLCXD3, MPZ, CALB1, TMEM132C, LOC400682, PGAP1, PHF19, MED22, GRAMD2, RAP2B, NRXN1, GDF2, DNAJC12, DENND2D, UNC5B, LOC283398, TBC1D4, C11orf54, KIAA1310, RNF182, AMMECR1, PRPF18, DUSP3, C9orf97, FECH, H2AFJ, PI15, ZCCHC14, TNFRSF10B, ARID3A, PITPNM1, C14orf45, NPFFR2, CIAO1, TRPS1, PTGER3, PCDHA9, PCDHA8, PCDHAC1, PCDHA12, WASF3, ENTPD7, FUT9, C17orf80, PSIP1, ISCA1, ZFP42, PCDHA6, PCDHA10, PCDHA13, NSL1, PCDHAC2, PCDHA5, GYS2, PCDHA1, IL7, PCDHA11, ATP6V1G3, PCDHA3, MIB1, RAB14, PCDHA7, GALNTL2, PCDHA2, PCDHA4, KIAA0196, PPHLN1, ATP1B1, USP37, AGPS, C1orf141, TM2D3, ATP2B1, ZNF304, RAVER2, DCX, ALG10B, C9orf123, PAPOLG, LOC100128006, LOC100130173, NUP160, TXNL4A, MCPH1, PPM1E, XG, RNF141, PRKCQ, THBS2, KAT2B, HLF, PBX3, AKAP2, PALM2-AKAP2, LOC727834, F8A1, ZNF227, COL6A6, OLFML2B, TRIM33, LRIT2, GYG2, QTRTD1, LOC153328, LOC642398, TMEM130, C5orf28, GANC |
| **hsa-miR-328** | A2BP1, LOC100133827, TSEN15, SARM1, ACPL2, CNOT2, DAPL1, CHRNA4, TMTC2, ESCO1, TNR, ANKRD23, C16orf89, LGR4, HIF1AN, WDR68, SLC5A7, RAB22A, PLCE1, PGM2, CLDN12, C3orf15, B3GALT1, PSTPIP2, NR3C1, PMP2, DDB2, LPPR2, C15orf57, NF2, KCNA7, EDC3, LYVE1, DIAPH2, C2orf30, TTYH3, ANXA11, EN2, SYNJ1, SNRK, MECP2, GPR116, EDARADD, TRIM39, HTF9C, ARHGEF3, LOC100134118, C6orf168, LOC100130311, TTC38, LOC100134245, MGC33894, LOC729010, DHDDS, REPS2, LOC648245, ARL6IP1, SCN9A, KNDC1, TRIM44, TRIM59, GEMIN8, UROC1, SNAP23, KREMEN1, ATAD3C, ZC3H7B, TMEM49, ATG4A, LOC100129034, PYGB, CXorf39, CLEC2B, FLJ14186, PBX2, FAM62A, GOLGA1, OTUD3, TIFA, C14orf82, YWHAZ, RSBN1L, CMTM4, FUT9, CA3, DLST, PTPN9, KIAA1147, RAD51L1, CD300LG, ARID4B, CRK, TESK2, RANBP10, MGC34761, VASH1, IPO9, MIB1, PTGIS, TIAM2, SFRP1, LOC348174, PPARD, LOC497190, CLIC5, ZAK, ACSS2, LOC338667, ARID1B, ORAI2, HIATL1, BDNF, PRF1, LRRTM4, KCNJ12, IL5, ORAOV1, RAG2, USP37, NECAB1, OLFML2A, KCTD16, DNAJA4, DNAJB1, KLHDC7A, CCDC76, CHST7, VSIG4, MAPRE1, FERMT2, FAM18B, CHRFAM7A, LOC651324, SLC2A1, FAM40A, LOC100130998, EPB41L5, FBXO10, NEUROD4, MAX, LOC100132901, ZSCAN22, SPTA1, SIX4, C1orf151, SOX11, USP8, C5orf4, ERMP1, ENTHD1 |
| **hsa-miR-1908** | ESPN, CMIP, CST9, LSP1, PRRT1, NLRP8, GRIN2D, ACSL3, NXPH4, SCAMP4, PITX1, IQSEC2, C7orf26, ISLR2, DHTKD1, SLC24A6, septin5, ABO, STK11, PABPC1L2B, SCRT1, PABPC1L2A, WNK3, LOC727748, ZNF784, PRSS22, C3orf70, CDKN2A, LOC644596, MUC21, UBE2NL, PRX, WWOX, SLC17A7, FAM43A, GJD3 |
| **hsa-miR-1972** | IGF2, FAM109A, MECP2, SLC9A2, BNIP3L, SLC41A1, TTC35, PQLC1, LPAL2, WIF1, C1orf21, GTDC1, ACYP1, PTMA, PTPRJ, FLJ40142, RGL1, MRPL49, TMBIM4, FAM120C, GBP7, RAB38, USP9X, RFPL4B, NCAPG, CDAN1, BCHE, DLG1, CASP10, ITPR3, LOC100128049, C3orf63, C10orf65, F11R, RBM33, FAM123B, FCRL2, PACS2, TNFAIP3, TMEM41B, C11orf49, ZMYND11, BRWD1, MAGEA5, HLTF, CCDC59, SH3BP4, DOPEY1, KIF14, HRG, SLC39A7, LOC100128090, LOC100132816, LOC400352, BACH1, TAL1, CASC1, LASP1, DDX17, LOC728320, LOC100129846, BMP8A, EPB41L1, UNQ1887, TRIM24, ACCN2, ADAMTS5, SCN4A, TCF7L1, ZFYVE27, IDH1, CMTM4, GNAI3, TMEM71, ACVR1, ENPP5, RSPO4, ARMC5, ZBTB44, COQ10A, ZNF45, LHFPL4, ZNF763, FANCC, IL16, GALNTL2, ODF2, C22orf29, PIK3CG, MEIS1, GLT8D1, SPATA21, PCDHB4, SMPX, MTHFR, RPL28, POU4F2, STX12, LOC51057, OR13A1, DUSP11, LOC100129323, LOC100133827, EFNB1, YTHDC1, WDR77, BAZ1A, BCR, REM1, CNTN2, CYP27B1, XPO7, SNPH, TBL1X, RHOJ, LOC643287, CACNB2, MDH1B, ZNF510, SH3PXD2B, ZWILCH, SEMA3C, CNTLN, TRIM6, ZNF783, GAS2, MBD1, PLEKHA6, CLDN9, ID3, PBX4, C5orf13, EMILIN3, LOC730015, FJX1, ALPK3, DEFB132, TMEM132B, FBXW8, DKFZP434I0714, MDGA2, TEF, PTCD3, GTSE1, TFAM, SLCO2B1, ZFAND5, CTNS, EDA, HDHD1A, PDE6A, DGCR2, NFAM1, PPP1R15B, PCYT1B, IKZF2, GGA2, RASSF6, RILPL2, hCG_2045710, ZBTB33, RNF41, PRKACB, HLA-DQA2, NIPA1, CCL25, C4orf34, CARKD, A2LD1, FLJ43582, ORAI2, ARIH2, ATAD3C, NAAA, GRB10, MIPOL1, LRRC17, TATDN2, FLJ41603, SRR, CTNND1, MAP4, LIN28, PPP1R9B, HHLA3, TTBK2, GART, SLC16A1, SMAD2, SPR, C5orf24, KCNJ10, MOXD1, ERBB4, IDS, PAK6, NPNT, LOC100128988, BLCAP, PHF17, SLCO1A2, ODZ4, LOC284067, PABPC4L, SRI, GNGT2, SIRPB2, OGFOD1, TADA3L, LOC100133276, TRDN, EFNB3, ARMC8, DAG1, PDCD6IP, CPNE5, LOC100129513, NOS1AP |
| **hsa-miR-1974** | SHMT1, C13orf31, ZBTB20, HEXA, LUZP2, RAD23B, ZRANB2, LOC100129185, TMTC1, C6orf97, KLHL31, PTGR2, ABCC4, C2orf73, RSPO1, FAM40B, ARL17P1, DOCK2, EPHA4, ITGB8, C10orf140, BMPR1A, RGS5, TP53RK, CDC42, OMD, AP3S1, LOC552891, GNG10, CCDC55, CLEC2D, LGR6, LOC100128054, ATP11A, PRKCA, PDZD8, CCDC25, IMPACT, C2orf56, EPM2AIP1, AMIGO2, HAS2, FAM78A, TNIP3, FAM122B, LRRC1, PEX13, SLC35A4, KLRA1, TXNDC3, LOC100127920, GBA3, MBNL2, PPP2R5E, SRFBP1, ITSN1, PCYOX1L, TLR10, CISD2, C12orf23, C6orf62, C8orf46, LACTB2, LHFPL3, POU2F2, ZNF764, MTRR, SLC2A13, ARL6IP5, WIPF2, LOC100132661, ST8SIA3, ARL17, C11orf61, ZIC1, GORASP2, FAM5B, HSPA1B, STK38L, C3orf17, MBD5, ZFX, PARP15, FAM105B, DPP6, SMC5, MBTPS1, RNF111, ZNF208, RHOBTB1, GNA13, MEX3B, hCG_1814486, VPS36, KAT2B, ASB5, KIAA0494, ORMDL2, LRRTM4, NOV, AGTR2, VGLL3, LOC645446, C7orf46, RAB11A, NT5DC1, PCYOX1, SMC6, AFAR3, LOC343052, CDH8, RNF125, LOC651820, SPATA5, CADPS, C20orf197, DNAJB9, WDR55, BID, C14orf104, SCN7A, KRTAP24-1, KLF11, BCO2, TAT, JPH1, CADM2, RFT1, MBD4, TMCC1, ALG9, SENP8, SOX18, DYNLT3, APPBP2, C3orf58, CD84, CDH19, C10orf47, ZCCHC8, CXorf50B, RWDD2B, MKI67IP, DDI1, TP53INP1, TNC, BRPF3, EXPH5, CDH13, FCRL4 |
| **hsa-miR-1977** | TRPC1, OLFML2A, LOC285556, TBX5, C3orf10, 7A5, PQLC3, CHST7, UBQLN2, GOLPH3, EIF2S1, SAMSN1, TMEM32, TRIM25, SPARCL1, CMBL, LOC727945, SSTR1, NUCKS1, MYC, NPHS2, CD274, GNA13, APOL4, KIAA1602, MBNL1, EIF2AK3, CPEB4, CAPN10, GRIA2, FEZ1, CUGBP2, BOLL, ACVR2B, CHRDL1, CDH9, ABT1, GNPDA2, MOBKL2B, MTUS1, C11orf61, XPA, ZDHHC2, HLA-C, DEPDC1, FOXF1, FUT9, ZNRF1, AICDA, ING5, PLGLB1, STOM, FAM104A, PLGLB2, ADAMTS6, LOC100134366, LOC100134363, LOC727773, ZRANB3, DNAJC14, SPTLC2, MIPOL1, SSR1, PDGFD, GLCCI1, CEACAM7, IL22RA2, PIP5K3, AKTIP, MNT, ZFP3, RFX5, YLPM1, VTA1, PLCL1, EBF3, ZFP106, VPS4B, WIPF2, CD200R1, PICALM, LOC100130665, C5orf41, FRMPD4, RPGRIP1L, SLC4A4, KCNMB2, C5orf22, PYGM, STXBP6, MAN2B2, GRPEL1, PTGER2, LOC100128281, WHSC1L1, PKNOX1, C5orf25, MAP4K4, RP3-439I14.1, PANX1, SLMO2, SNW1, RAP1GDS1, PDE11A, IKZF3, C8orf68, ARPP-21, KBTBD11, ICA1L, LOC389024, PHOX2A, KIF27, C12orf66, NCAPD3, NHS, LOC100129901, AVPR2, MAP3K5, LOC100130420, ZNF175, LOC100132082, UBR1, ARHGAP29, PTPRE, LOC100134499, BACH1, ZNF33A, FAM159B, CETN2, LOC100134809, DNMBP, ALS2CR8, KRT26, ZNFX1, ZBTB4, LOC340239, LOC100129000, KSR1, C7orf59, MRPS14, YME1L1, CDC7, LOXL4, FPR2 |
| **hsa-miR-638** | CCNG2, WDR47, TCERG1L, SLC46A3, GPR116, SUMF1 |
| **hsa-mir-1978** | CLDN18, A2BP1, CAP1, ACTR3, SLC26A7, ZSWIM6, CDH23, SGTB, IGF2BP2, ARHGAP6, GDI2, TMCC1, VPS37A, SLC30A7, ERMAP, FBXL20, YTHDF3, LOC100130600, ZXDC, GALNT7, OR51E2, IDS, BTG2, LOC100134513, LOC730351, ZNF136, KIF11, SLMAP, PCDH17, PRDM15, ASAM, CRISPLD1, FUT9, MCFD2, CDH19, PERP, SLC38A4, SGOL2, C1orf27, DYNLL2, INHBB, PAPSS1, TGFBR3, CLDN1, CLTA, LMO2, EIF2C4, PTPRZ1, CD2AP, HLF, B3GALNT1, PHYH, CTCF, RBPMS, C2orf30, UQCRB, ALOX12B, ARHGAP5, FGF13, ERMN, TRAPPC6B, FRAT1, DDX52, FAM134B, SCYL1BP1, TMEM26, PTPN13, MTDH, CCDC6, SERPINB4, SCN8A, ZBTB8, RNF169, ZNRF2, FOXK2, TDRD10, RAP2C, TXNDC6, AKTIP, ARL1, AQP9, CHM, NEGR1, CPA4, EIF1AX, RP11-345P4.4, ROBO1, LOC399715, PABPC1L2B, RBM43, SERPINB3, PABPC1L2A, ANKRD46, CBLN2, LOC401220, FAM20B, HMCN1, LOC646329, CRIPT, ZNF706, FSHB, POU3F1, CAPN7, C8orf4, LOC100131605, GRSF1, LOC641798, SCN3A, MPZ, C4orf33, ASB8, LOC220906, CDC20B, COL4A3, ANTXR2, LOC100134011, PMM2, AK3, ZNF470, RAB30, CDK6, YAF2, FAM91A1, TBC1D8B, CTSL2, ALDH1A2, AKAP1, ARHGEF9, USH2A, TMED2, C17orf62, WIPI2, SPATA5, KLHL6, GTF2E2, RGS6, EML4, C5orf24, COL9A1, NPTX1, RNF13, ZFYVE20, QKI, ELL3, PLCXD3, LOC100129781, YWHAZ, ABCC1, AREG, AKAP13, LOC100130729, EP400, CSGALNACT1, NFX1, ACSL5, CD5, MYB, GOT1, C16orf63, NOS1AP, XIRP2, C2orf37, SEMA4A, BRUNOL4, TRIM39, PRKAA2, GBP6, MTMR4, DDX21, NR3C1, TUBD1, ZRANB2, POLR2K, HUNK, MYLK, WDR21A, GFRA2, SELI, GAS7, LOC729971, SLC16A6, ZNF441, HAS3, OMD, ELK1, CDADC1, ENOX1, GNG12, SMARCD1, RUNX1T1, MITF, IYD, MRS2, RUFY3, C11orf73, ZNF527, C10orf18, ATN1, EPM2A, IMPAD1, NKAIN3, C5orf4, ZNF91, KANK2, ARHGAP26, CSNK1G1, PIP4K2A, CLEC16A, ZNF771, THAP5, SPAG1, UBLCP1, POU4F1, DNAJC21, WDR40A, MEF2D, FLVCR2, PER2, METAP1, ASAH3L, PPM1B, LOC100134211, HS3ST3A1, SNAI2, VGLL4, C5orf22, KLF4, BCAS2, GPLD1, SLC10A4, CEP27, TMEM55A, GNG10, B3GALT2, CRTAP, KIAA1524, SNAP91, TRIM6, ANKRD28, ZNF594, SH3RF1, GLIPR1, MYO5B, TINP1, OR12D3, STX11 |
| **hsa-mir-1201** | SCHIP1, DGKB, SAMD12, GPD1L, C6orf64, SIAH1, CNOT8, STEAP2, C10orf118, WISP1, WWP1, TMEM27, MRPL40, SRF, CHP2, MEX3C, UFM1, LSM8, GPX8, KIFC2, RECK, AAK1, BMPR1A, ZNF681, EML4, KRTAP20-3, NLGN4Y, ZNF638, NT5DC3, LOC100133431, CNBP, PPT1, RNF43, CCDC109A, BRWD1, IMPG2, ATP2B3, FAM134A, C6orf204, MEF2A, MRS2, LOC100132161, LOC100132678, PPARA, CCNC, WDR68, BACH1, ZNF330, PKD2L1, TMEM30B, GLT25D1, GAPVD1, C20orf108, MAL2, ARSF, SIDT1, IL2, KRR1, CHP, MEIS1, CAB39, PDHA1, SRBD1, GRPEL2, ZNF514, LOC100134192, C7orf51, ATP6V1A, PSAP, PTGIS, KCTD14, RRM2B, HPGD, LOC441440, TMEM199, TTBK2, MBOAT2, DUT, KPNA1, TRHDE, hCG_18385, CLUAP1, ZNF25, TXNRD1, FERMT2, ENAH, SMARCA2, TAT, SLC20A1, FRMPD4, LOC100130965, SREBF2, LANCL3, EEPD1, ZNF197, NR4A3, CNTD1, DDAH1, PTPDC1, ZNF81, TM7SF3, DNAJC25 |
| **miRNA identifier** | **Computational targets** |
| **hsa-miR-866-3p** | CDK2AP1, C14orf4, MAFF, KCNJ15, GDAP1, MBNL2, LRRK2, CAPN3, TMEM80, GZF1, BCAS1, GOT1, CACNB1, KPNB1, TBX18, AZIN1, GABRP, TBX15, IL8, CTAGE5, CEP68 |
| **hsa-miR-29a** | ZBTB7A, SLC8A2, RP11-251J8.3, CXADR, septin6, FOXA3, USP41, SH2D4A, JMJD5, SPARC, SCUBE3, SLC36A1, LY6E, TTYH2, LHX6, NKAIN4 |
| **hsa-miR-106b** | NOVA1, SLC1A2, FAM169A, ARHGAP5, ERCC4, MAP4, C10orf141, TLR6, CNR1, PITX2, RBM4B, MFAP3L, AKAP8, ZNF81, EVI5, UNC45B, ZXDB, LOC100129165, TFPI, ANKRD50, ZC3H6, BMPR2, VGLL3, PROS1, AKAP7, C13orf18, RDH10, APPL1, SHROOM3, SEMA3A, ENAH, CXCL5, SOCS6, IKBKAP, ANKRD22, C11orf30, GABRA1, KLF11, PTEN, CFTR, WIPF1, ZNF543, BTN2A2, EVI2B, CSNK1G1, MYO6, DZIP3, FGFR2, septin9, B3GALT2, LRRC19, ZFHX3, NR2C1, ZNF25, MYLIP, NAV3, ZBTB8, YAF2, SRFBP1, VCPIP1, DPYD, BCL2L15, SHOC2, RFX7, CGGBP1, PRTG, LARP2, LRAT, BACH1, PITPNB, WDR26, SCD5, SLC26A3, CRKRS, CMPK2, ROCK1, FBXO34, SLC6A15, RB1CC1, KIAA0895, CAPS2, CUL4A, NAALADL2, TNKS, ACSL4, ANKS1B, DCP2, ATXN3, DSG2, MTUS1, PRKAB2, C2orf21, ACVR1C, PCDH19, 7A5, KIF3A, GRAMD1C, JAKMIP2, PDE4B, DDEF2, MYST3, NAB1, ATP8A1, JMJD2C, PCLO, NMNAT2, PBRM1, TMEM135, DNAJA2, HNMT, NAP1L2, LOC652725, MGA, GALR1, TET1, NADK, USP31, TCEA1, SCAMP1, FER1L3, COL6A6, SLC39A6, ARHGAP11A, PDS5B, HS2ST1, VPS13A, CAMK1, ZNF233, WDR89, DNAJC6, DCP1B, USP47, PDCL3, ZKSCAN2, CCT5, KIAA1147, NBEA, FGL2, DNHL1, C1QTNF7, EXOC8, C11orf54, SLC7A11, TACC1, GALNT7, LOC642558, EIF5, TMTC3, ZC3H12C, TMEM154, GULP1, SLC30A10, RNF138, RTKN2, INSC, HADH, LGR4, ZNF654, OSBPL6, TBC1D3G, LEFTY2, PIGA, AGPS, TMEM87A, POLR3G, SFRS7, SFRS17A, ATP8A2, NHLH2, ZIC2, TRDMT1, GCC2, EIF2C4, C6orf151, SENP3, FBXO9, KIAA0776, PDIK1L, ANXA11, FAM111A, PTGER3, KIF4A, C1orf26, H2AFB3, ZRANB3, GIN1, FAM155A, ENPP1, USP48, LOC647286, TDP1, PGAP1, LOC100133836, UBP1, ELF1, FBXW11, ARHGAP29, TWSG1, SERPINE1, SMN1, SEC24B, ARHGEF17, ARHGAP28, LOC100133835, HEPACAM2, MEGF9, C1orf198, SMN2, RAPGEF5, RPP30, GLCE, ZFP62, UBE2L6, SERPINB9, TSHR, LOC653382, ARFGAP3, RAI14, TMED2, COL4A5, CPT1A, SYPL1, SNAI2, LYSMD3, C15orf29, TRPS1, FGF14, LUZP2, ABCG5, GALNT1, PDLIM4, GPLD1, PPFIA1, HEATR5B, RWDD4A, TACC2, C1orf149, FNBP1L, FAM159B, UACA, ZNF264, HOXA10, PLSCR4, H2AFV, INTS2, BBS7, CEP120, ZNF705A, PPP2R3A, TNRC18, C11orf41, LOC145814, DBX2, PCOLCE, ZNF705G, GLI3, C9orf150, CSGALNACT2, NNT, C14orf82, ZNF705D, FLJ42562, TCP10L, USP6, LOC100134348, CTR9, AK5, LOC100131539, TLE1, SHISA2, HDAC9, USP32, CLPTM1L, POU3F2, SMURF2, ENTPD1, CCNT2, CLEC4D, PLEKHG1, MORC3, SFRS2IP, KHDRBS1, IGF2BP1, PHF16, GABRA5, SPATA16, PPM1D, IL1RAP, RGPD5, RGPD8, API5, SH3PXD2A, TBC1D3C, TBC1D3F, RFWD3, LOC729837, GPR158, LOC653380, TBC1D3E, ZFX, LOC653498, BCLAF1, A1CF, MED21, TBC1D3H, GPNMB, RGPD6, ZNF767, RGPD4, RGPD3, KLRC4, SLAIN2, RAP2C, PI15, C6orf91, USO1, ZFHX4, WDR90, LMOD3, CASP2, ZNF207, CYP26B1, FMO5, SLC30A4, SLC25A16, IL1R1, LIPH, LBA1, LOC282992, LOC653550, UTP15, ZBTB41, LOC729264, CHD9, STAT1, EIF2AK2, SLC35F5, S100A1L, CAND2, BOLL, NAAA, SERPINB4, HS3ST3A1, LOC643475, RHOXF2B, SNX9, SERPINB3, MDFIC, ZC3H7A, KCTD10, RAD51, CD28, RHOXF2, LOC100130053, ARID4B, BCAP29, C11orf61, HMGCS1, RRS1, LOC729240, IL26, WDR33, ADAMTSL3, YTHDF2, FLJ40296, TGIF1, BCOR, LOC729250, EDN1, LOC729246, DUOX2, ZBTB44, DSCC1, GK, ZCCHC8, MORF4L2, EIF2C3, NFKBIZ, KIAA1244, FAM3C, HMP19, LOC729233, GANC, ZNF621, ZNF750, TMEM26, SMARCE1, PLS1, BHLHB2, C5orf39, NLGN4Y, POM121, MAGEE1, TMEM90A, POM121C, KLHDC10, PTER, ZC3H12B, LOC100133431, SLC5A8, SAP18, REEP1, ZNF396, TBC1D14, KPNA1, FAM35A, SOCS5, GALNT2, NXPH1, ELMOD1, PSG2, FAM102B, FZD6, PTPN12, PPP1CB, SLC35A5, WDR44, UBASH3B, EML4, PPM1A, TXNL1, KIAA1632, H3F3B, CDH17, CAV2, FOXL1, ARHGEF10, PHYHIPL, TAOK3, SLC46A3, HAS2, MUT, HNRNPA3, RNF8, LONRF2, LOC552891, STAM, MTMR9, PPWD1, WWC3, PRDM4, SMAD1, TEX15, POLR3F, IL6ST, ENPEP, NPNT, LRFN5, PCDHB10, CCDC59, NPHS2, GNG10, WDR45L, ZSCAN23, BCHE, LYCAT, HIST1H2AG, CSMD3, FAM91A1, ARNTL, SCARB2, TARSL2, B4GALT6, MRPL43, SH3BP2, KMO, FSTL5, AFF1, MBNL2, LOC100128191, RAB40B, G3BP1, PTPRB, ADRA2A, TUBA1C, DSC3, UBE2J1, SEMA5A, SPSB4, CHCHD8, SEH1L, ARPM1, GFPT1, STON1, GAGE1, RAP1B, GPRC5B, UBQLN2, EPHB1, POLS, tcag7.1228, CH25H, RBM39, DSC1, RICH2, FIGF, ANKHD1, GGNBP2, WDR37, SOX1, ZBTB39, IL17RD, SFXN1, AOF1, PRDM6, CRIPT, CCNYL1, PXMP4, SOCS4, C1orf144, CCDC141, KLHL7, MLLT4, TOR1AIP1, MAP3K7IP3, ZNRF2, GRIK2, KIAA1549, LATS2, SLC7A2, LOC100133914, ALG8, MAP4K4, PPP1R12B, TEAD1, ELF2, HLA-DQA1, CDK6, TCEA2, NAALAD2, STARD3NL, BMP2K, DERL1, LMOD2, CD5L, RNF103, FREM2, LOC161527, LRRIQ3, NEDD4, FOXJ3, PUS7L, MAGEB4, ELOVL5, BMPER, PHEX, ZNF711, SH3PXD2B, ZNF12, MFN1, UGT2A3, STOML3, TAPBP, RAB5A, MICAL1, DENND5B, PFDN4, ABCC12, GUCA1B, KCTD14, UGT2B17, CNOT6L, IL16, LOC100129095, SEC22B, TMPO, IL12B, RERE, LOC283788, HIVEP1, CTBS, ABCA1, ZNF460, PDP2, LOC727944, ABCC5, FAM29A, C18orf54, OSGEPL1, PAK7, IFI44L, GPR26, COPB1, SLC5A7, CYB5A, ANP32E, TMEM19, ZNF257, MMD, ZNF281, C5orf32, PTPRK, C16orf57, ALG10B, LOC100132735, C12orf66, PGR, DDX17, SFRS2, MSL3L1, SDK1, GCLC, CCDC108, LOC100134141, WDFY1, CHCHD3, IL1A, FAM116A, UNC84A, SLFN11, CNIH, TTC14, ANKFY1, LOC100132892, TXNDC8, LOC285620, MS4A6A, SLC38A1, RASA3, TBC1D2B, LOC729272, LOC100133649, USP15, PHF8, GRPEL2, RET, PAK1, FLJ36131, ASF1A, PRKAA2, SUSD1, ZPLD1, SCFD1, SRP54, GLDN, C21orf63, GLIS3, PTGS2, GPR85, PSEN1, EN2, LOC645202, LOC440243, LOC283767, PDCL, PECAM1, JUND, RFK, DBN1, LOC727942, PHACTR2, PCSK1, TAC1, PMAIP1, MYC, JMJD1B, FAM120A, PLEKHA2, TRAPPC10, NAMPT, IKBKB, SPCS1, SKIL, LOC644914, PAP2D, TOX4, LHFPL3, MXD1, DCN, CCDC4, POF1B, RB1, PPP1R8, PIGK, TMEM187, MNDA, KLHDC6, CAMTA1, ANKRD6, AAK1, SH3RF3, STX6, KLHL13, TLK2, RAD51L3, F5, STK3, KIAA1045, ELOVL3, C10orf28, KIAA0515, MLL, ST8SIA2, WDR1, RAPH1, C17orf100, LYSMD4, FAM136A, WDR20, MBD5, GPR103, ZNF623, ZNF425, UPF2, ISCA1, FBXO48, UBQLNL, CREM, LAPTM4A, LCORL, ERLIN2, BBS10, PURB, LOC100130886, LOC644538, MSI1, HOXD13, LOC729355, SLC38A9, USP36, CFHR5, LOC100130890, C5orf24, RRP15, LOC100133862, RAD23B, TP53TG3, KBTBD3, C8orf37, WHSC1, RAB12, TGOLN2, FLJ42177, NUTF2, TXNDC10, CMTM4, WDR35, ALDH1B1, KIAA1715, UNC5D, ZMAT1, septin8, RAB11FIP2, LOC100134367, LOC100134211, BRMS1L, APOBEC4, PCDHB9, RALYL, SEC62, SHISA3, SKAP2, KIF2C, HEATR3, ETV3, C14orf4, CYP2J2, SPTLC3, LOC647795, TRAPPC2, EIF1B, CDS1, ARHGAP20, TNFSF10, SRGAP1, LRRFIP2, CUGBP2, MAP2K1, MYNN, C1orf55, SETD7, POLQ, PRPF4B, C3orf64, YWHAE, TBL1X, IGF1R, ADRBK2, GLT6D1 |
| **hsa-miR-107** | CSNK1G1, GNG13, PNPLA7, FBXO34, ZNF329, CCDC52, GLT8D3, C12orf4, SLC1A2, KIAA2018, KLF3, TNPO1, LOC100134785, XDH, ZNF346, CUBN, SH3TC2, GPR39, HRB, EPS8, CREB3L2, VPS53, ZFP41, CLK2, ADAM9, C3orf34, UNC5D, ANO6, MORC3, LOC100130216, VPS35, USP9Y, RGS5, LOC100133609, DCTN4, tcag7.1228, ENC1, ATAD2B, KRAS, RXRB, SNN, C1orf173, MCHR2, BAALC, DENND5B, CCRL1, C10orf25, ARFGEF1, ZNF84, SRRM2, PPP1R9A, NAPB, SNX10, BTG1, AQP4, LIN7C, NEO1, PDZD11, ZNF44, PPARGC1A, SLC5A3, BNIP3L, TRIM44, CTBP2, ELAVL2, GRSF1, DSP, ZNF514, ELOVL5, XRCC6BP1, FN1, QKI, AP1GBP1, RCAN1, TNRC6B, ZNF200, SBF2, FAM35A, RAPGEF5, TLR7, PRKCSH, MKLN1, PHF19, SERP1, ATP6V1G2, MPPED2, C14orf108, PURA, GCET2, SCN1A, CAGE1, ADAMTSL3, CENTB2, CRIPAK, USP47, NEBL, KRR1, LYRM7, KIAA0586, ZNF599, YTHDF3, CYTIP, CNTNAP2, KLC1, CNR1, MAL, UHRF1BP1, CHST6, LOC100133876, MGAT4A, SLC26A9, LOC100131686, C14orf147, KLHL12, LOC100133607, DDAH1, PFTK1, SLC6A3, TP53INP1, SAPS3, LONRF2, KIAA1328, TRIM10, ZBTB1, METTL10, ARMC4, BTBD11, CA13, RGS16, PXN, NR2C2, ADD3, MT1F, CCDC82, AGPS, MAGI2, USP31, GTF2I, C16orf55, TMEM173, ERAP1, LAMB4, MSL2L1, SURF6, PCP4, SCML4, DTNA, ZNF197, CTNND1, TMEM50B, VSX2, SYN2, PPAP2B, BHLHB5, TRAPPC10, CYP39A1, DCP2, PAP2D, ADH1B, STAT1, POLI, THUMPD1, ARHGEF9, NKIRAS1, LIFR, MTF1, SARM1, LOC100131180, LCORL, NDUFS1, SEMA4F, QRSL1, TBC1D9B, HSDL1, PLCXD3, LOC100134022, NCALD, SLC16A12, MRE11A, GRB10, KCNB2, ASF1A, LOC100133205, EI24, ZNF665, CSNK1A1L, RBPMS2, PDXK, PEX5, JARID1A, AP2B1, FIZ1, RBM12B, KAT2B, ZNF562, HHLA3, SMAD2, SMC1A, CHD1, PARP12, TRIM2, UGT2B15, EPS15, UBXN7, BTN1A1, SOD1, ALDH5A1, LOC728160, EYA1, MAP2, DNAJA2, BDKRB2, PDGFD, RAC2, CBLB, ZNF257, SMCR8, C16orf61, SMAD4, C10orf105, MID1, CRK, ALG9, GPX7, ZNF440, TMEM66, TPR, GALR1, IMPA2, DNASE2B, TRPM3, DCX, LOC100130890, TEAD2, DGKB, DISC1, MRPL14, SH3BP2, UBE2K, CYP1B1, KBTBD4, IKZF2, LOC100134259, CYP8B1, C8orf40, FBXO31, SLC30A8, HHIP, AK2, PTER, C11orf30, RORB, CDC40, STX5, FLJ35379, EVI2A, C12orf69, WDR33, ARHGEF12, TLE4, ZDHHC17, KAL1, KLHL4, LOC727942, PSRC1, SH3GL3, LOC646509, ZNF189, CTSO, MALT1, SCN9A, COL5A3, NIPA2, LOC100128403, FAM184A, JPH1, HCG27, C9orf152, LOC728320, DCUN1D4, USP4, WDR43, ACOX3, CAMK2D, ZNF624, SFMBT2, SSBP2, C1orf21, GRPR, PET112L, HEMK1, SNTA1, MAGOHB, MAPK14, MPPE1, FAM53A, STX17, EDIL3, ZC3H4, EARS2, ZNF275, MRO, KCNMB2, KRTAP6-3, ARFGAP3, septin14, ABCA9, RAB40C, TBR1, LOC100133749, MITF, ZC3H12B, INO80D, SNX30, LOC732437, CDK6, CCDC65, HIPK2, UGT2B28, NEU1, UBE2A, EVI5, ANKRD34B, FAM123B, DDT, ST8SIA1, TXNIP, LOC100132436, TOX4, LOC100133711, GRM8, ZDHHC7, FGL2, FAM105B, GNE, SYT1, MAP3K12, LATS2, KCTD14, NODAL, MLPH, NAPEPLD, PDLIM5, DLGAP2, GMFB, TTR, TSHR, ZNF415, INHBE, PIP4K2B, LOC730011, ZNF609, LOC100129858, UBA5, ERLIN2, LOC730236, ONECUT2, POLD3, LOC100130955, KL, MIER3, KIAA0802, OTUD4, PTPRN2, ZRANB2 |
| **hsa-miR-17** | GSG1L, SGOL1, KLRA1, RTKN2, MSR1, FAM53C, CREBL2, SLC16A7, BRWD1, SMG7, SEMA6A, ZNF267, GLRB, C6orf168, TMEM123, SCN3A, CCDC4, MUT, CLCC1, MYOT, SEPHS1, AGL, PRKAA2, PCSK6, TMEM184A, ATF1, KLHL14, DYNC1I1, DNAJB14, GPR161, SH3TC2, MCCC2, LOC730432, FAM45A, ZNF518B, ERBB4, C12orf71, PSD3, DYRK1A, LOC100132163, METAP2, MALT1, JARID1A, ZNF502, C3orf43, PIP4K2A, ANGEL2, SGK269, PRO0628, MSN, PHLDA1, TPRG1L, FAM153B, SEC63, CMTM6, BACH1, SYT14, ZNF566, GPR85, LOC727942, LY75, LOC100129413, AASDHPPT, HELZ, NCAPH, DMRT2, ARSJ, ZNF714, UBE2A, SORL1, ZCCHC6, YAF2, WDR77, SOCS4, FAM19A5, ZNF124, DUSP18, CLIP4, PROM1, IDS, SLC38A2, PTPLAD2, DLG1, DDI1, SLC25A27, NLRP3, IYD, C10orf30, KIAA1370, KCNS3, SLC35C1, VIM, C5orf36, KIAA0776, FAM84A, GNRHR, ADAMTS6, TSPYL4, CDH2, CAMK2G, FLJ20674, LHFPL3, STXBP5, RAD50, PTAR1, BAHD1, CHKA, DTL, LOC100132051, MITF, CD22, ACVR2A, CDO1, THRB, E2F8, TSPAN31, LOC26010, NDFIP1, IHPK1, CTSC, FRMD6, REPS2, FMNL3, C18orf34, RLBP1L1, EDA, GOLGA1, RRN3, AXIN2, SCML2, GRHL1, PRELP, RGMB, C20orf26, LIN54, LOC728849, UBE2G1, USP54, HCCS, SPAM1, ATRX, PMP2, PLEKHB2, FAM151B, KIAA0895, ACBD3, HECTD2, GRM1, SYT16, MGC3207, ARFIP1, CLEC6A, VPS53, ANKRD42, GPR26, LAX1, FSTL5, MIER1, ZNF425, KIAA0355, MIER3, KIAA1715, C16orf45, NIPBL, PAIP2B, KLC1, GRHL2, AP1S3, TAOK1, INPP4B, ESR2, BCL2L2, C10orf90, ZNF770, KCNAB1, EPC2, VCPIP1, GAPVD1, AFF3, LOC153469, C9, ZNF845, C21orf45, ALG10, SLAIN1, KIAA1377, SOX2, FAM98A, ZNF697, ANKFY1, SCN1A, ROPN1, DERA, IQCA1, NPAT, CLEC4C, CHST9, FGL2, SLC43A2, DCP2, XK, TET1, EHF, RIMBP2, ACADL, PEG3, PAPD1, PHYHIPL, GLIPR1, LOC100134793, AKAP9, TAF9B, QKI, LOC391343, LOC100130332, FGF5, ZIC3, IRAK2, FLJ44054, LOC286177, CISD1, DSCC1, EXOSC9, CALM2, ARL4C, FNDC3A, FLJ41481, CDC25A, FIGF, HHAT, ZNF254, FERMT2, KITLG, LRRTM2, UBXN2B, C16orf63, PLEKHG2, TNFRSF8, PCM1, TAP2, PML, C4orf40, TUBGCP3, SPCS2, RP5-1000E10.4, NDUFA5, ERCC4, CCNG2, RPP30, ADAT2, TRDMT1, CCDC88A, ZAK, ZNF470, GDAP1, MRPL19, RNF125, FLJ35220, GIGYF2, PLCXD3, MPZ, CALB1, TMEM132C, LOC400682, PGAP1, PHF19, MED22, GRAMD2, RAP2B, NRXN1, GDF2, DNAJC12, DENND2D, UNC5B, LOC283398, TBC1D4, C11orf54, KIAA1310, RNF182, AMMECR1, PRPF18, DUSP3, C9orf97, FECH, H2AFJ, PI15, ZCCHC14, TNFRSF10B, ARID3A, PITPNM1, C14orf45, NPFFR2, CIAO1, TRPS1, PTGER3, PCDHA9, PCDHA8, PCDHAC1, PCDHA12, WASF3, ENTPD7, FUT9, C17orf80, PSIP1, ISCA1, ZFP42, PCDHA6, PCDHA10, PCDHA13, NSL1, PCDHAC2, PCDHA5, GYS2, PCDHA1, IL7, PCDHA11, ATP6V1G3, PCDHA3, MIB1, RAB14, PCDHA7, GALNTL2, PCDHA2, PCDHA4, KIAA0196, PPHLN1, ATP1B1, USP37, AGPS, C1orf141, TM2D3, ATP2B1, ZNF304, RAVER2, DCX, ALG10B, C9orf123, PAPOLG, LOC100128006, LOC100130173, NUP160, TXNL4A, MCPH1, PPM1E, XG, RNF141, PRKCQ, THBS2, KAT2B, HLF, PBX3, AKAP2, PALM2-AKAP2, LOC727834, F8A1, ZNF227, COL6A6, OLFML2B, TRIM33, LRIT2, GYG2, QTRTD1, LOC153328, LOC642398, TMEM130, C5orf28, GANC |
| **hsa-miR-103** | A2BP1, LOC100133827, TSEN15, SARM1, ACPL2, CNOT2, DAPL1, CHRNA4, TMTC2, ESCO1, TNR, ANKRD23, C16orf89, LGR4, HIF1AN, WDR68, SLC5A7, RAB22A, PLCE1, PGM2, CLDN12, C3orf15, B3GALT1, PSTPIP2, NR3C1, PMP2, DDB2, LPPR2, C15orf57, NF2, KCNA7, EDC3, LYVE1, DIAPH2, C2orf30, TTYH3, ANXA11, EN2, SYNJ1, SNRK, MECP2, GPR116, EDARADD, TRIM39, HTF9C, ARHGEF3, LOC100134118, C6orf168, LOC100130311, TTC38, LOC100134245, MGC33894, LOC729010, DHDDS, REPS2, LOC648245, ARL6IP1, SCN9A, KNDC1, TRIM44, TRIM59, GEMIN8, UROC1, SNAP23, KREMEN1, ATAD3C, ZC3H7B, TMEM49, ATG4A, LOC100129034, PYGB, CXorf39, CLEC2B, FLJ14186, PBX2, FAM62A, GOLGA1, OTUD3, TIFA, C14orf82, YWHAZ, RSBN1L, CMTM4, FUT9, CA3, DLST, PTPN9, KIAA1147, RAD51L1, CD300LG, ARID4B, CRK, TESK2, RANBP10, MGC34761, VASH1, IPO9, MIB1, PTGIS, TIAM2, SFRP1, LOC348174, PPARD, LOC497190, CLIC5, ZAK, ACSS2, LOC338667, ARID1B, ORAI2, HIATL1, BDNF, PRF1, LRRTM4, KCNJ12, IL5, ORAOV1, RAG2, USP37, NECAB1, OLFML2A, KCTD16, DNAJA4, DNAJB1, KLHDC7A, CCDC76, CHST7, VSIG4, MAPRE1, FERMT2, FAM18B, CHRFAM7A, LOC651324, SLC2A1, FAM40A, LOC100130998, EPB41L5, FBXO10, NEUROD4, MAX, LOC100132901, ZSCAN22, SPTA1, SIX4, C1orf151, SOX11, USP8, C5orf4, ERMP1, ENTHD1 |
| **hsa-miR-191** | ESPN, CMIP, CST9, LSP1, PRRT1, NLRP8, GRIN2D, ACSL3, NXPH4, SCAMP4, PITX1, IQSEC2, C7orf26, ISLR2, DHTKD1, SLC24A6, septin5, ABO, STK11, PABPC1L2B, SCRT1, PABPC1L2A, WNK3, LOC727748, ZNF784, PRSS22, C3orf70, CDKN2A, LOC644596, MUC21, UBE2NL, PRX, WWOX, SLC17A7, FAM43A, GJD3 |
| **hsa-miR-130a** | IGF2, FAM109A, MECP2, SLC9A2, BNIP3L, SLC41A1, TTC35, PQLC1, LPAL2, WIF1, C1orf21, GTDC1, ACYP1, PTMA, PTPRJ, FLJ40142, RGL1, MRPL49, TMBIM4, FAM120C, GBP7, RAB38, USP9X, RFPL4B, NCAPG, CDAN1, BCHE, DLG1, CASP10, ITPR3, LOC100128049, C3orf63, C10orf65, F11R, RBM33, FAM123B, FCRL2, PACS2, TNFAIP3, TMEM41B, C11orf49, ZMYND11, BRWD1, MAGEA5, HLTF, CCDC59, SH3BP4, DOPEY1, KIF14, HRG, SLC39A7, LOC100128090, LOC100132816, LOC400352, BACH1, TAL1, CASC1, LASP1, DDX17, LOC728320, LOC100129846, BMP8A, EPB41L1, UNQ1887, TRIM24, ACCN2, ADAMTS5, SCN4A, TCF7L1, ZFYVE27, IDH1, CMTM4, GNAI3, TMEM71, ACVR1, ENPP5, RSPO4, ARMC5, ZBTB44, COQ10A, ZNF45, LHFPL4, ZNF763, FANCC, IL16, GALNTL2, ODF2, C22orf29, PIK3CG, MEIS1, GLT8D1, SPATA21, PCDHB4, SMPX, MTHFR, RPL28, POU4F2, STX12, LOC51057, OR13A1, DUSP11, LOC100129323, LOC100133827, EFNB1, YTHDC1, WDR77, BAZ1A, BCR, REM1, CNTN2, CYP27B1, XPO7, SNPH, TBL1X, RHOJ, LOC643287, CACNB2, MDH1B, ZNF510, SH3PXD2B, ZWILCH, SEMA3C, CNTLN, TRIM6, ZNF783, GAS2, MBD1, PLEKHA6, CLDN9, ID3, PBX4, C5orf13, EMILIN3, LOC730015, FJX1, ALPK3, DEFB132, TMEM132B, FBXW8, DKFZP434I0714, MDGA2, TEF, PTCD3, GTSE1, TFAM, SLCO2B1, ZFAND5, CTNS, EDA, HDHD1A, PDE6A, DGCR2, NFAM1, PPP1R15B, PCYT1B, IKZF2, GGA2, RASSF6, RILPL2, hCG_2045710, ZBTB33, RNF41, PRKACB, HLA-DQA2, NIPA1, CCL25, C4orf34, CARKD, A2LD1, FLJ43582, ORAI2, ARIH2, ATAD3C, NAAA, GRB10, MIPOL1, LRRC17, TATDN2, FLJ41603, SRR, CTNND1, MAP4, LIN28, PPP1R9B, HHLA3, TTBK2, GART, SLC16A1, SMAD2, SPR, C5orf24, KCNJ10, MOXD1, ERBB4, IDS, PAK6, NPNT, LOC100128988, BLCAP, PHF17, SLCO1A2, ODZ4, LOC284067, PABPC4L, SRI, GNGT2, SIRPB2, OGFOD1, TADA3L, LOC100133276, TRDN, EFNB3, ARMC8, DAG1, PDCD6IP, CPNE5, LOC100129513, NOS1AP |
| **hsa-miR-301** | RPS6KA5, DDX6, EIF2C4, PIGA, C1orf144, SOS2, MIER1, UBL3, TBL1XR1, MTMR9, C5orf30, VPS37A, LOC100128178, FBXO28, BTAF1, ACVR1, MYBL1, SLAIN1, PHF14, ABHD3, LDLR, CNTNAP3B, EPB41L5, LOC643827, LOC642373, AL953854.2, C10orf140, TNRC6B, ZNF217, C7orf60, CENTD1, PAN3, GOLSYN, ELK3, JARID2, ZFYVE26, DCP2, NUP133, TMEM50B, PHF3, SNIP1, MTMR10, STIM2, GOLT1B, SMOC2, SH3D19, WDR47, MET, RBM25, ZFPM2, PGM2L1, MEOX2, MEMO1, CBFB, LIPA, CDC2L6, NIPA1, ARL6IP1, FSTL5, IRF1, CLTC, ST18, LONRF1, PIK3C2A, RAB5A, USP28, DYNC1LI2, ARHGAP12, IGF1, KBTBD8, HSPC159, UBE2D1, ENPP5, ATP6V0A2, MLL3, HABP4, HBP1, ALDH3A2, GJA1, RNF145, C2orf15, WDR20, ASXL2, FAM59A, TMEM32, SYT6, BTBD3, NPAT, CHST1, GADD45A, ROBO2, USP48, PMEPA1, CFL2, PSAP, C18orf1, PDK1, GTF2H1, STEAP4, PTPRG, RTCD1, SULF1, CCDC6, FBXL11, WNT1, GDA, ACSL4, PARVA, NHLH2, PLAA, EPS15, PCDHB4, UBR7, ST8SIA3, MBNL1, SKP1, NPTN, KIAA1468, SERBP1, NCOA1, SNX2, EREG, HS3ST5, SGCB, CLIP1, ZDHHC23, OBFC2A, ESR1, AAK1, B4GALT5, FBXO48, NPEPL1, UBA3, DOCK3, DENND4C, KIAA0256, C16orf70, AKAP1, ADAMTS18, SESTD1, MAT2B, SAP18, FXR1, KCTD16, CCT6A, KIAA0802, NMUR2, LYCAT, ZNF614, RNF38, RP5-1022P6.2, TSC1, LOC653355, tcag7.1228, RNF216, RAN, ERBB2IP, CNTNAP3, LRRK2, ATG16L1, UBE3B, MREG, ULK2, OTUD3, RFX7, C21orf66, KRT23, TCF4, SMARCD2, PTEN, WASL, ZNF238, EIF4E3, UBE2D2, tcag7.1317, NEUROD1, LOC727944, SC4MOL, KLHL20, FRMD6, MTMR6, COX8C, DNAJC16, TGFBR2, CAV2, C3orf59, SNRPE, HOXC8, PEX5L, PRKD3, SMOC1, TMEM55A, FAM43A, WEE1, CNOT4, CEP55, FMR1, ERCC4, CTAGE4, LMTK2, MDFIC, GALNT13, EIF2C1, C3orf64, ATG2B, PDZD11, LOC100131208, NPNT, MPHOSPH9, LIX1, HIVEP2, INHBB, TRIM37, TOM1L2, ARX, ZAK, FAM53A, FAM179B, IER3IP1, FLJ20518, TMEM9B, ARHGAP21, HHEX, CXCL6, BMPR2, EIF5A2, UBXN2B, FBXO9, TRIM59, DCDC2, ZIC5, APPL1, NDEL1, ZEB2, E2F7, C22orf30, CLCN5, TMEM194B, GPATCH8, ACPL2, MID1IP1, MLLT10, KIAA0831, RAPGEF4, DYNLL2, BAI3, SNX27, SYT16, CAMTA1, SNAP25, RALBP1, FRZB, ATP6V1B2, NAP5, AMPD3, ITFG3, CAST, PSD, QKI, SNX5, NRBF2, HFM1, UNC13A, BTBD10, LOC100134741, ARFIP1, NBPF20, LOC100129280, CLCN6, TGFA, CBY1, ZNF853, RAB30, RASA1, CENPO, HOXA3, PTH, LOC100128716, GMFB, SOX4, BHLHB3, CEBPE, LOC727942, ADAM12, PHACTR2, CDKL2, S1PR1, FAM35A, C7orf53, C3orf58, MYT1, POU3F2, CEP170, LRP2, SERINC3, IER5L, CMPK1, JAKMIP1, NR3C2, LOC728934, ATAD3C, CERCAM, FOXF2, LOC730755, PLSCR4, SLMAP, CD69, CASP8, MPP7, PPARG, LOC728285, ANGEL2, MECP2, TRIM23, FOSL1, FYCO1, SLC6A6, FIBIN, ZNF609, ENPP6, ITGB8, DPYSL2, RPGRIP1L, LOC729979, CHD9, FZD6, ATXN1, TRIM2, EPC2, CCDC88A, TMEM63B, LRIG1, TGOLN2, CHMP4B, HPRT1, KIAA1217, PTGER2, TAF4B, RTN1, BIRC6, TWISTNB, ZNF3, SLC44A1, KIAA0528, FAM73A, WDR33, SLC2A2, MAFF, LOC100131551, METTL4, CYLD, ANKIB1, RFXAP, FBXL5, ZNF250, ATP6V0E1, NME7, CSF1, CALM2, CAMSAP1L1, RABL5, ANXA4, F3, MUM1L1, HCFC2, LOC439949, RAB40B, MYO1D, GATAD2B, ZDHHC14, ITPR1, DEPDC1, GLIPR1, LOC100132979, HCG27, CHCHD3, MLL, DUS4L, C15orf54, SERPINE1, LYSMD3, SPOPL, THSD7A, HOMER1, RAB34, ZCCHC14, RAG1, PBOV1, RSU1, FAM104A, CRISPLD1, CHRNB2, ZBTB40, CHD5, UBR1, R3HDM1, PIP5K3, IGSF3, FAM46B, CCDC85A, CCDC137, PPARGC1A, VPS13D, CCDC126, RBM17, PRR15, ADCY1, UBQLN1, ACBD3, TSHR, USP31, MEX3D, LRP12, SPG20, MMP13, KIAA1191, GAP43, MARCH2, PTPN20B, ERAP1, C8orf4, MED12L, PTPN20A, SRGAP2, DNAL1, CAPRIN2, MDM1, TNRC6A, CTSK, TFEC, PRSS16, BZRAP1, ZFP91, COX10, SBF2, PRKAA1, NRP1, TBC1D8, SOCS5, UBE2J1, POU6F1, IKZF4, INSIG1, CCNYL2, ABCB7, CLUL1, PTP4A1, ACBD5, ATP11A, KIAA1486, SH3PXD2A, PLCB1, DIP2A, WHSC1L1, SPHK2, CRCT1, CELSR1, CDV3, SDC4, PRICKLE2, C6orf211, DLG2, NCKIPSD, CASD1, TRIM3, ITPRIPL2, TNFRSF1B, PHF20, MMP10, CGGBP1, INO80, CSMD1, DSG1, IL23R, PHF17, PIK3IP1, HCCS, ADAM28, UBAP2L, LOC100130729, CSK, NOL4, SGTB, WDFY4, KLHL3, SMAD5, RAD51L1, EPHA10, MATN3, VCPIP1, FANCA, LOC100131023, CSNK1G1, SLC8A1, HSD17B11 |
| **hsa-miR-20a** | FYCO1, ZNFX1, PLEKHA3, NAPEPLD, MAP3K2, AAK1, BRMS1L, ADARB1, PKD2, PDCD1LG2, RPS6KA5, ARHGAP12, C2CD2, ZFYVE26, ZNF264, NEDD4L, ENPP5, CYBRD1, FNBP1L, C14orf28, SAMD12, FCHO2, DDHD1, ITPRIPL2, EIF5A2, MTMR3, VSX1, TNFRSF21, RNF128, PPP6C, GPR6, ATG16L1, TBC1D20, DYNC1LI2, EPHA4, LOC100134306, GOSR1, RAB22A, FGL2, MYT1L, SERF1A, KLHL28, SERF1B, SMOC2, SLAIN2, FBXL5, NFAT5, TXNIP, RUFY2, RSBN1, FGD4, C15orf17, IPO9, PTPRT, CEP97, NCOA3, CROT, ANKRD52, NPAS2, TGFBR2, FGD5, ARID4A, ANKH, GNPDA2, SORL1, FBXO48, KATNAL1, MAGI3, SLC7A11, KIAA0513, ZNF800, SACS, SR140, TRDN, ANKIB1, USP3, HN1, RBL1, C7orf43, LIMA1, TM2D2, PAK7, DUSP2, PGM2L1, BCL11B, RAB11FIP5, FAM129A, LMOD3, VLDLR, ITGB8, SH3TC2, CERCAM, C19orf2, PBX3, HIF1A, TNFAIP1, ETV1, CAMK2N1, PURB, NR2C2, RGMB, CCNG2, TMEM127, LOC100130053, EIF4G2, SLC46A3, NAT12, C10orf78, PAG1, DERL2, PTPDC1, AFF2, GPR137C, C11orf30, OBFC2A, ANKRD29, BMPR2, LAMA3, ULK1, CDC37L1, BTBD10, CHD9, FAM40B, SLITRK3, MTERFD2, PRRG4, UBE2W, SSX2IP, ARHGAP26, TMCC1, MKLN1, E2F5, UNK, PAFAH1B2, CAMTA1, OXR1, ARID4B, NKIRAS1, RGL1, ZNF512B, TSHZ3, AHCTF1, FIBIN, TRIP11, CXCL6, YOD1, FRMD6, MINK1, DPYSL5, AGGF1, LOC729637, MKRN1, IRAK4, C11orf79, C7orf60, NOTCH2NL, ZFP91, GOLGA1, PCDHAC2, BCL10, PFN2, STAT3, PHC3, SGTB, F3, ERAP1, MCHR2, INTS6, EGLN3, TNKS2, MFN2, PAPOLB, MASTL, MMP2, KIF23, CYP2U1, KPNA2, FAM148A, RASL11B, SPOPL, CDKN1A, LOC653355, VASH2, CNTNAP3, C1orf63, MAPRE3, FAM134A, CNOT4, PPP3R1, CNTNAP3B, YPEL2, LOC643827, GAB1, SLC22A23, CTSS, YTHDC1, ZNF295, OLFM3, PBK, AL953854.2, LOC642373, PTGDR, MMAA, CFL2, RRM2, MED12L, RAB10, LAPTM4A, EGLN1, EZH1, PPP1R3B, KAT2B, ESR1, PKNOX1, BTG3, MAT2B, C5orf41, GLIS3, FTSJD1, TET3, CMPK1, FRS2, TIMM17A, PRRG1, COL4A3, SOS1, LOC100128510, SSH2, PAPOLA, LOC649970, MFAP3L, SAR1B, LOC731391, TMEM123, ITFG1, LOC100128039, XRN1, BCL2L15, NAGK, PTPN4, CRY2, SLC16A9, ITSN2, E2F1, GNB4, FNDC3A, WDR37, PTHLH, TWF1, KLHL20, SLC40A1, TNRC6B, LIMK1, MAP3K8, FBXO39, TMEM138, LRRC10, NPAT, UBXN2A, DOCK4, TMEM192, SERP1, CLEC4D, CDCA7, KIAA0922, RAB11FIP1, ABL2, DCUN1D3, LHX8, RASSF2, ZFPM2, NR4A2, APBB2, ZNF532, FAM57A, EIF4H, KIAA1191, BAHD1, CHIC1, MAP3K9, TRIP10, SMAD7, ST8SIA2, CHAF1A, RP5-1022P6.2, DIP2A, POLR3G, SUMF1, RACGAP1, RGMA, TAOK3, SYTL4, TMEM25, MCM3, RBBP7, P2RY14, ST6GALNAC3, CHP2, RNASEL, RAP2C, RAB5B, C2orf21, PRR16, CCDC128, COMMD6, RSRC2, PHTF2, LPGAT1, RELL1, ATAD2, TOPORS, RNH1, MORF4L1, MKNK2, CD69, CDC40, GPATCH2, PCDHA10, LRRC20, BAMBI, NUP35, PCDHA12, KIAA1383, PCDHA6, PCDHA7, FAM8A1, DMTF1, HBP1, SENP1, BRWD1, UBE3C, C2orf64, PCDHA11, ARHGEF3, PCDHA4, CYP20A1, GALNT3, PON2, LDLR, PCDHA5, LOC100132249, LOC643395, PCDHA13, RNF125, GNS, PCDHA3, PCDHA1, PCDHAC1, PCDHA2, SEMA5A, C7orf41, HAS2, KLF10, FAM102A, IL17RD, FAM13C1, RP5-1000E10.4, ABCG2, ZAK, VANGL1, WEE1, UEVLD, EID2B, PTPN3, PPARA, UBR5, APOBEC4, FOXJ3, USP31, BNIP3L, AGTPBP1, KIAA1128, DKFZp667G2110, DENND5B, TMEM167A, FNDC3B, MAP3K14, DNAL1, CRK, WASF3, SGMS1, SYT16, NPAS3, LHX6, OSM, FAM126B, NTN4, XRRA1, JAK1, IFIT5, RNF6, NRBP1, C12orf36, RUNDC1, ADAM9, MAPRE1, PNPLA4, CDC14A, ZBTB33, NR4A3, LYST, KIAA0574, ARL4A, SLC25A36, YTHDF3, CENPQ, DRD1, STK38, KIAA0494, ATXN1L, HECTD2, STK17B, ANKRD12, ANKRD50, PBLD, SPG20, RNF145, ZBTB9, ANKRD28, NR2E3, MAP7, ANTXR1, MCL1, ZNF148, NIPA1, EIF2C1, PPP3CA, CSGALNACT1, USP24, AP2B1, KIF26B, KLK7, TSG101, DPYD, RPGR, HSPA8, CRIPT, GABBR2, FJX1, ZNF367, PIGM, ADAT2, GNB5, RBL2, GJA1, PHLPPL, USP28, ENSA, MYCN, FAM123A, POLQ, C9orf82, CTSK, ENTPD4, LRRC45, BMP8B, TRIM3, USP46, FAM117B, CLIP4, PFKFB3, OTUD4, ZBTB7A, NLRP3, ANKRD57, ASAH1, TPRG1L, GLO1, RASD1, BICD2, BTN3A2, C18orf32, MDFIC, HS3ST5, FOXL2, SRrp35, LACE1, PEX5L, NMUR2, EREG, LOC100133296, ZNF280B, RHOC, SPRED1, GK5, UTP14C, TUSC2, CNN1, C14orf138, ZBTB47, OSTM1, ZBTB8, SAMD9L, ZFYVE9, LOC100134763, ZBTB6, CNOT7, NBEA, FGF5, NAP5, NY-SAR-48, SC4MOL, CASP8, LOC100131022, SOD2, CAPRIN2, AKAP5, AADACL1, FBXW11, LOC100134440, C2orf69, RBM12B, ORMDL3, OR7D2, LOC100130111, CLSTN1, PHACTR4, MYO1D, CTAGE4, TMUB2, RIMBP2, ZNF238, ELAVL2, DUSP8, STK33, SOX4, PAM, PIP4K2A, RNF149, LAMP3, ATL3, SNX11, LOC100134369, SERTAD2, SUV420H1, CEP120, GOLM1, FBXO21, WDR33, KIAA1598, DPY19L3, B3GALT2, PXDN, FBXO31, REPS2, ZNF362, RSAD2, ZNF697, MDM4, DMP1, ZIM3, F2R, TGM2, ARL1, ZBTB4, FAM53A, OSR1, SCARA5, MPZL2, SDC2, FAM177A1, GNPTAB, CEP57, STYX, LRRC57, FBXL11, PCDHA8, TCF7L1, PCDHA9, TBC1D15, MUM1L1, SCN1A, ACSL4, TANC1, PLAGL2, PLSCR4, CCL1, KIAA1627, MKL2, AHRR, CAPN7, BNIP2, ZFP3, TXNDC10, WHSC1, ZNF25, PKHD1, SLC2A2, ZSCAN20, MLXIP, PPP1R15B, PPP2R2A, CNRIP1, WWP2, MEX3D, EPHA5, UCP3, PTGR2, C3orf63, DPYSL2, CPNE1, L3MBTL3, APCDD1, TLE4, MXI1, TBC1D9, PDLIM4, MED17, LOC338809, CASC4, ZBTB41, ATP1A2, PTPN21, PFKP, ACADSB, ARL4C, MGEA5, REEP5, MTCH2, C4orf39, BTBD7, WFS1, CASP2, CREB5, LYSMD3, MUC17, UBE2B, C1orf135, RAN, KBTBD8, LONP2, VPS26A, RABEP1, CHRM2, NPL, LOC388177, PKIA, LOC100131936, NPLOC4, PDE3B, CNTN3, MMP3, NOL4, SGK269, IQSEC2, IL8, MAP3K5, CRCT1, BTN3A1, TET1, PDGFRA, THAP2, FXR1, NPM1, ABCG4, TMEM154, CENTD1, KIAA1147, NBL1, PGBD5, ZBTB38, NPHP3, C1orf173, PKN2, CCDC14, EIF2S1, IKIP, TAGAP, TP53INP1, NSL1, GUCY1A3, ACPL2, TRIM36, DGCR14, NEUROG1, PLEKHG1, C9orf40, PRR15, RFXAP, RP1-21O18.1, CCDC93, PLCXD3, GTDC1, DIRC2, SCN2A, ABI2, GABRA1, SNX9, AK3L2, SYNE1, FZD6, FBLN1, F2RL2, TRIM37, KLF12, METAP1, FBXO40, ARHGAP1, LOC731985, ARCN1, LMO3, IKZF4, KPNA3, MFSD9, RNF2, GOLSYN, C14orf82, CRIM1, RCCD1, TBC1D2, CNR1, SASH1, ATP12A, NANOS1, FAM80A, ACBD5, OLIG1, SLC30A7, ZFYVE16, RBM20, EEA1, NETO2, DNAJB6, GALNT6, DUSP18, DMRTA2, FOXJ2, HEG1, PGP, C18orf19, RB1CC1, ORAI1, JRKL, LOC100130373, TRIM55, CYP4F3, ELK3, TRPV6, MGC26718, LOC732096, ITCH, AMIGO2, TIMP2, LOC286177, IRF9, SEMA4B, C6orf201, VDAC1, SFRS14, RGPD5, DDX5, C20orf197, MSTO1, RHOV, IQSEC1, ABCA10, STX6, MCC, PPM1A, SNIP, C21orf66, HPS5, ANKRD13C, LOC152742, ATG12, LOC100134134, TBC1D8B, MLL4, MSR1, PDGFD, CALD1, MSTO2, RND3, ZNF473, FOXQ1, TNRC6A, HOOK3, INO80, TNFSF11, HLF, STRBP, ZFYVE20, KCTD18, RB1, THEX1, FAM46C, MYO10, SCN3A, COX8C, THAP6, MYNN, RNF24, UBE2Q2, MAP3K1, SRGAP2, NR2C2AP, BVES, LOC730432, TACC1, TMEM194B, KIAA1377, MAPK9, SPRY4, SSR1, RAPGEF4, FLJ41200, EFHC1, IPO5, ABT1, CORO2B, STC1, WDR48, HMGB3, CRLS1, PRDM6, CASP7, CTSA, FRMD4B, LDLRAD3, SH3PXD2A, UXS1, MRPL43, SALL1, ZNF239, TRIM8, GBP3, FURIN, RAPGEFL1, SOLH, ARHGAP5, TBL1X, WDFY3, IRF1 |
| **hsa-miR-106a** | SH3TC2, FYCO1, AAK1, ARHGAP12, DYNC1LI2, BMPR2, PLEKHA3, EIF5A2, RAB22A, KATNAL1, ZNFX1, ADARB1, FCHO2, RPS6KA5, PAG1, CYBRD1, NCOA3, NEDD4L, ITPRIPL2, PKD2, MAP3K2, PPP6C, MTMR3, KLHL28, BRMS1L, DDHD1, PGM2L1, ARID4A, GNPDA2, SLAIN2, TMEM127, C2CD2, PDCD1LG2, ZFYVE26, ZNF264, TXNIP, RSBN1, TNFRSF21, SAMD12, NAT12, ATG16L1, EPHA4, NAPEPLD, MYT1L, TMCC1, SERF1A, SERF1B, UBR5, LOC100134306, TBC1D20, ENPP5, FGD4, SMOC2, DOCK4, GPR6, RUFY2, FBXL5, ANKH, C14orf28, SLC46A3, VSX1, PBX3, SPOPL, CHIC1, TGFBR2, RNF128, MAGI3, GLIS3, SORL1, LIMA1, STAT3, ETV1, PPARA, NPAS2, FGL2, ANKRD29, XRN1, KIAA0513, RGL1, CROT, DERL2, CHD9, YTHDC1, VLDLR, USP3, SSX2IP, ARID4B, MFAP3L, GJA1, LMOD3, GOSR1, MTERFD2, AFF2, PURB, SLITRK3, C7orf43, PAK7, TNFAIP1, UBE2W, PAPOLB, CDC37L1, GPR137C, HIF1A, FGD5, CEP97, CAMK2N1, FNBP1L, PHC3, SSH2, ANKRD52, CERCAM, C15orf17, FIBIN, OBFC2A, RASSF2, PRRG4, C7orf60, EIF4G2, RBL1, OXR1, DUSP2, TRDN, RAB11FIP5, BCL11B, RGMB, C19orf2, LAMA3, RAB10, MAPRE3, NKIRAS1, PTPDC1, ZNF800, PPP3R1, CCNG2, MMP2, PTPRT, COL4A3, ITGB8, SR140, CNOT4, GNS, PKNOX1, ZNF295, SLC16A9, C10orf78, ABCG2, ZFP91, TSHZ3, SGTB, PAFAH1B2, ARHGEF3, TRIP11, E2F5, PKN2, SLC7A11, SACS, USP28, MAP3K14, PPP3CA, PHTF2, ITSN2, FAM134A, ZNF512B, LHX8, MINK1, TMEM192, AGGF1, PFN2, RP5-1022P6.2, LDLR, F3, PFKFB3, ZNF148, TNKS2, LPGAT1, KPNA2, EGLN3, VASH2, ZBTB9, TM2D2, MFN2, CTSS, OLFM3, RBBP7, CLIP4, FTSJD1, KIF23, BTBD10, WDR37, ZNF697, TMEM123, CNTNAP3B, SENP1, TNRC6B, NR2C2, FAM40B, CDKN1A, AHCTF1, IRAK4, CFL2, LOC643827, PBK, AL953854.2, ULK1, C5orf41, C11orf79, FAM148A, LOC642373, KAT2B, IPO9, BCL2L15, KIAA1128, RASL11B, ANKIB1, HN1, XRRA1, FBXO39, TMUB2, MKRN1, ESR1, UBE3C, SLC22A23, ZNF714, PAPOLA, FAM126B, MCHR2, TIMM17A, RRM2, SOS1, IL17RD, RBM12B, GAB1, CMPK1, EZH1, UNK, MAT2B, ARL4A, MUM1L1, EGLN1, C1orf63, SYTL4, ERAP1, MASTL, PTGDR, ZNF532, DENND5B, ITFG1, DPYSL5, NOTCH2NL, BCL10, BTG3, CYP2U1, GOLGA1, CAMTA1, TWF1, FAM57A, KLHL20, STK17B, LOC649970, LOC100128039, KIAA0922, CRY2, LOC731391, PCDHAC2, INTS6, GALNT3, FBXW11, MAP3K8, PTPN4, SRrp35, ABL2, DCUN1D3, NAGK, ANKRD50, E2F1, SAR1B, MMAA, KLF10, RNF145, MAP3K9, PTHLH, SUMF1, MAP7, LOC100134440, LRRC10, CLEC4D, P2RY14, CDCA7, FAM129A, NR4A2, TMEM138, NR4A3, LOC729637, NFAT5, SLC40A1, TET3, MYCN, VISA, JAK1, MORF4L1, DIP2A, FRMD6, RAB11FIP1, CREB5, C11orf30, BRWD1, RELL1, CCDC128, PHLPPL, RSRC2, SMAD7, BAHD1, ST8SIA2, MED12L, ATAD2, MCM3, ZFP3, CRIM1, LOC653355, SERTAD2, PON2, TRIP10, HAS2, RGMA, CHAF1A, ZFPM2, POLR3G, FNDC3A, WEE1, SERP1, TAOK3, LOC100132249, SEMA5A, CNTNAP3, PRR16, HECTD2, EREG, FBXO48, RAB5B, LOC643395, TPRG1L, PRRG1, CTSK, TMEM25, DMTF1, USP46, RAP2C, AGTPBP1, PDPK1, MRPL19, COMMD6, ATXN1L, RNF125, BMP8B, GPATCH2, MCC, RNASEL, SYT16, C2orf21, CXCL6, LHX6, NAP5, C2orf64, CHP2, ZNF362, MPZL2, ARHGAP26, APOBEC4, CDC40, ANKRD12, KIAA0574, LOC100133296, KIAA1383, SGMS1, BAMBI, HBP1, MKLN1, NUP35, PKHD1, STK38, BNC2, PPP2R2A, IFIT5, NRBP1, TMEM167A, NPAS3, NBEA, EID2B, CASP8, SOX4, UEVLD, TBC1D8B, SPRED1, IQSEC2, PTPN3, KIAA1191, DKFZp667G2110, EIF4H, PPP1R15B, LIMK1, SPG20, TP53INP1, CSGALNACT1, MAPRE1, RUNDC1, KLF12, NTN4, ARL1, AADACL1, KIAA0494, FMNL3, KLK7, ENSA, PNPLA4, PLCXD3, CD69, PIGM, HS3ST5, UBXN2A, ANKRD28, WASF3, FAM13C1, MTCH2, APBB2, NLRP3, RACGAP1, RNF6, LYST, LAPTM4A, ACADSB, USP31, TOPORS, CDC14A, F2R, ZNF367, ZAK, NR2E3, WDFY3, ZSCAN20, ADAM9, LRRC45, CENPQ, EIF2S1, FGF5, HLF, MCL1, ENTPD4, FOXL2, ANTXR1, TSG101, VANGL1, DNAL1, HDHD1A, CTAGE4, FAM102A, POLQ, SNX9, MYO1D, BNIP3L, BICD2, CNR1, C9orf5, HSPA8, SUV420H1, NMUR2, CRK, PTPRJ, MDFIC, PEX5L, PCDHA13, HEG1, PCDHA1, C9orf82, TRIM3, LACE1, PCDHA12, NIPA1, PCDHA3, GABBR2, ASAH1, PCDHA6, PCDHA7, YOD1, PCDHA10, PDGFD, TAGAP, PCDHAC1, PCDHA11, GNB4, FAM117B, PCDHA4, PCDHA5, ANKRD57, PCDHA2, WDR33, SC4MOL, JRKL, ATP1A2, C18orf32, WHSC1, ST6GALNAC3, ABI2, TUSC2, GLO1, RNH1, PPP1R3B, NETO2, MKL2, GNB5, RASD1, DUSP8, USP24, SLC25A36, ZBTB47, KIF26B, EEA1, RHOC, OLIG1, ZC3H12C, GNPTAB, DRD1, TANC1, MKNK2, SAMD9L, LOC100131022, CAPRIN2, SCARA5, NY-SAR-48, RFXAP, LRRC20, C10orf97, STK33, LOC100130111, EIF2C1, C14orf138, ACSL4, LOC100134763, EXOC5, CNN1, DMRTA2, SOD2, B3GALT2, CNOT7, ACBD5, KIAA1598, CLSTN1, RNF149, PIP4K2A, TET1, C12orf36, DPYD, C7orf41, ANKFY1, THAP6, UXS1, FNDC3B, MDM4, ARHGAP5, AP2B1, LOC100130053, ATL3, FAM53A, CALD1, ARL4C, MYNN, SNX11, ZNF238, PAM, UTP14C, REEP5, PXDN, ZBTB33, FAM177A1, SCN2A, C12orf5, CNTN3, GTDC1, ORMDL3, ELAVL2, ATG2B, ABCA1, NOL4, FAM123A, CEP57, KIAA1627, UBE2B, VPS26A, BTBD7, SLC2A2, FAM8A1, PTH, DPYSL2, RP5-1000E10.4, RBL2, DMP1, RSAD2, CEP120, TLE4, MYO5B, YTHDF3, CCNT2, GABRA1, AKAP5, PGPEP1, FBXO21, MEX3D, TGM2, OSR1, ZNF280B, LOC388177, NANOS1, CCL1, C14orf82, ELK3, FAM105B, ZIM3, WWP2, CPNE1, FJX1, RAN, ZBTB4, RND3, LOC338809, OSTM1, TXNDC10, AGTR2, CRIPT, RPGR, FBXL11, PGBD5, CNRIP1, PDLIM4, BNIP2, GIPC2, THAP2, L3MBTL3, ZNF25, CYP20A1, FRS2, UCP3, MYLK3, STX6, FZD6, TRIM37, ZFYVE9, DDX5, SPTLC2, RPS6KA2, ZBTB38, SLC16A12, BTN3A2, NPAT, TMEM154, APCDD1, OR7D2, CCDC14, ATP2B1, LOC100131936, CRCT1, CPEB3, ADAT2, C4orf39, PDE3B, KIF5C, DGCR14, GATAD1, NSL1, WFS1, KCTD18, C1orf135, IL8, C1orf173, SPTBN1, CHRM2, MAP3K5, MAP3K1, ERBB3, INPP5F, HRH2, POFUT1, CASP2, ACPL2, MUC17, PLEKHG1, BTBD9, C3orf63, C2orf69, METAP1, C9orf40, ABCA10, BCL2, FBXO40, CENTD1, MSTO2, ZNF704, NBL1, MMP3, SMOC1, PRR15, RP1-21O18.1, FBXO31, TCF7L1, LOC731985, DNAJC27, BTN3A1, TNS1, MSTO1, ABCG4, TIMP2, NPHP3, DPY19L3, ATG12, ARHGAP1, TBC1D2, ARCN1, FOXJ3, NEUROG1, PANX1, SLC25A27, ZADH2, FBLN1, GUCY1A3, SLC30A7, RABEP1, LOC732437, NPL, ZBTB6, NPLOC4, PTPN21, DNAJB6, SASH1, PBLD, ZBTB7A, IRF9, LMO3, LRRC57, SOBP, LOC100128510, PGP, PTGR2, PLS1, TTPAL, DLGAP2, TMEM194B, TACC1, LYSMD3, MIDN, EPHA5, STYX, MGC26718, TMEM56, RGPD5, C6orf201, YPEL2, PCDHA9, GTPBP10, PCDHA8, MTF1, E2F2, KIAA0317, WNK3, QKI, SCN3A, NHLRC3, CASC4, ZBTB41, GALNT6, OCRL, LOC732096, PDZD11, SFRS14, RIMBP2, RB1CC1, C8A, AMIGO2, HPS5, DUSP18, ATP12A, ATP8B1, C21orf66, TXLNA, YPEL1, C18orf19, SPRY4, RB1, UBE2Q2, TBC1D15, PLAGL2, PHACTR4, C2orf67, TMEM182, AHRR, LONP2, MFSD9, RHOV, IKIP, VDAC1, FOXJ2, IQSEC1, GOLSYN, SEMA4B, WAC, FOXQ1, MLXIP, TNFSF11, THEX1, TRIM55, NR2C1, MRS2, LOC100134134, ANKRD13C, OTUD4, TAOK2, RNF24, GOLM1, MAPK9, SH3PXD2A, LOC100134369, IRF1, OSM, SNIP, SALL1, SYNE1, MXI1, INO80, PKIA, GABPB1, SPTY2D1, MSR1, ELL2, TNRC6A, TMEM133, HOOK3, ZNF320, HMGB3, MED17, ABCC5, NPM1, PLEKHG4B, STRBP, SUSD1, BCL2L11, MGEA5, RAPGEF4, REEP3, LOC100130373, GBP3, CAPN7, PRDM6, ASF1A, ALDH9A1, C1orf9, ERG, KLF3, ZNF473, RAPGEFL1, TRPV6, ZNF239, CRLS1, CCDC137, COX8C, KIAA1377, WDR82, CASP7 |
| **hsa-mir-18a** | INADL, HIF1A, THEX1, PHC3, TMEM170B, KCNA1, ESR1, NEDD9, GLRB, MAP7D1, JARID1B, CDC2L6, SULT1C2, QKI, FAM3C, CA13, KCNJ2, HSF2, HCFC2, TRPC4, ATM, ZNF367, DSC1, RNF145, HMBOX1, NCOA1, RABGAP1, NKIRAS1, PHF20L1, ETV6, CREBL2, HNMT, TNRC6B, ASXL2, ALCAM, PARD6B, VPS13A, RAD51AP1, KCMF1, KLHL20, ORAI3, LOC100128071, RFC4, CHRM2, TMCC3, DOCK4, SMAD2, DIP2C, PRKACB, HMGCS1, ZC3H6, KPNA6, RBBP8, SDC4, MBNL1, EPB41L1, PHF2, CYP4F11, FRYL, SAR1A, TBC1D9B, FCHSD2, SH3RF3, PDGFC, INPPL1, ACAD11, TXK, ZCCHC3, TTPAL, EHMT1, GABRA4, ESCO2, ERLIN1, CEP57, TNFAIP3, DSG4, VTCN1, LOC100129268, PARP11, PSD3, DCLRE1C, PLA2G1B, UBTD2, LRRFIP1, RIMS2, UQCRQ, CAD, TMEM2, FAM73A, RAB9A, PFTK1, LOC221710, NUFIP2, DAAM2, PDE4D, PHF19, LSM14B, MAPK4, LOC728946, C5orf30, KLF6, IRF2, PNLIPRP3, OPRM1, SEC23IP, FAM8A1, KRTAP1-1, UQCRB, ATXN1, SLC35B4, CTGF, ADD3, RAB5A, BRWD1, POLR3F, KLHDC5, NEDD4, ZDHHC23, FLJ41856, ZNF365, DCUN1D4, C6orf65, DICER1, LOC100133945, ENC1, C20orf30, LOC729122, ZNF709, JUB, BTG3, GUCY1A3, HEXIM1, LOC100129211, LOC100133946, PCID2, LOC728903, SRGAP3, ST8SIA4, ZNF470, EYA4, KCNMA1, TRIB2, C11orf87, C1orf9, ZHX2, KIAA0746, KCNS2, LYCAT, ITGA2, PTGFRN, AP3S2, C1orf173, TRIOBP, SLC40A1, DENND3, CENTA2, AEBP2, TBPL1, ANKRD13C, ZNF536, KIAA0999, TOR1B, HHIPL2, HMGN2, TLL2, MEP1A, GCLC, NHSL1, RNASE9, WDR68, KIAA0513, C7orf42, ACSL3, PRDM6, FAM149B1, SEC61A1, LOC100130557, LOC100134390, KCNH8, ARL15, OLFML2B, KRTAP24-1, LOC644006, RNF4, FBXL11, LOC388177, DDX18, NEO1, WASF3, LOC648691, TRADD, FKTN, RNASEL, SMAP2, LOC100129175, MAP3K1, PAPSS2, ZNF84, BTN1A1, IDH3G, ZNF501, KIAA1012, FLJ78302, KCNH7, DZIP3, C9orf70, SON, CCR2, SH3KBP1, TGFBR3, POU6F1, TUBB4, NR1I2 |
| **hsa-mir-31** | RSBN1, LOC728054, VPS53, ZNF74, LOC728417, PEX26, NSF, HSD17B6, SH2D1A, TRIM67, C10orf4, CREG1, PIK3C2A, LATS2, MYO5A, KIAA1967, PDZD2, SEC24A, FLJ14186, MTL5, ATP11C, PAX9, PRDX3, SERPINB8, ACSL4, PDAP1, PPP2R2A, PRKCE, SAPS3, TACC1, C2orf67, DCBLD2, MBOAT2, FAM123B, RHOBTB1, ETNK1, SLC6A6, RNF144B, BEST3, LOC729010, CAMK2D, GLT8D3, LOC100133422, SYNC1, CLCN3, AFF1, PPP1R9A, TBX19, SYDE2, UBE2K, WDR5, RASA1, UGT3A1, LBH, RIMS3, RGS4, IGSF11, JMJD6, JARID1A, ECHDC1, INSC, RAPGEF5, GSTCD, TSGA10, USP28, KANK1, KCTD21, SLC2A4, ZNF512, TM2D2, PAXIP1, C1orf25, SATB2, LOC100134757, LOC100131258, FZD3, TMPRSS11F, CD55, MAGEA1, EDNRB, LOC100129754, KCNA6, VGLL3, ARHGEF2, TFRC, OAS2, IL1R1, HIF1AN, NHSL1, VPS26B, NUP153, COPS2, GNAI3, BTBD11, TBXA2R, hCG_38480, SLC5A3, ZNF275, AKAP7, APBB2, LITAF, DKFZp667G2110, DPY19L2, TMED10, RAB23, STX12, CLASP2, CDC42EP4, MAP4, C1orf150, PPP3R2, LOC100131332, KIAA1429, RNF144A, HIAT1, CIAPIN1, CAB39, EIF5, CDC42SE1, KHDRBS3, MLXIP, RFWD3, GBP3, PVRL4, KIAA1024, MAGEA2B, CACNG4, SLC1A2, TMEM145, MAGEA2, C17orf78, SP1, HLF, EGLN3, FLJ43692, HLA-A, VEZT, TRAM2, SERPINA5, MAGEA12, PARP1, GLT6D1, SDC3, GPR183, FTMT, SCCPDH, NARG1, STARD13, KIF4B, LOXL4, TMEM161B, LOC644714, ATP8A1, ARID1A, RHBDL3, CTNND2, YLPM1, SRD5A1, MET, SLMO2, HRH4, C19orf12, SSH1, OPRL1, C20orf11, ZNF662, RNF135, GTF2E1, HADH, PXMP3, HSPC159, ASB13, LASS6, ZDHHC18, MAP1B, FCGR1A, SNX18, PLCXD3, PSMB11, MAPKAPK2, ZFP30, GAS8, MAGEA9B, MAGEA9, CARD8, HAO2, NAGA, SNX4, FAM134B, SH3TC2, KSR2, FAM110B, CDC20B, C13orf37, CDC23, MRPL38, LOC100129380, DDX3X, YWHAE, RND3, ZNF3, PEX5, MUTED, C11orf57, FMO4, OXSR1, MAGEA5, NUMB, TESK2, AIM1, CALU, AHCYL1, MTFR1, MRPS16, SUPT16H, VTI1A, KIF13B, DUSP19, C12orf52, MGAT1, KIAA1211, WDR42A, SEPHS1, TTC23, ZNF573, XRCC5, ZBTB7C, PLXNA4, WWC2, RAB5B, PPP6C, KRT6A, LOC728351, LOC729446, SHC4, FUSIP1, TNS1, LOC100127909, LOC642558, TBX5, DCX, FLJ44450, LASP1, TACC2, BTBD3, PCDH21, HPGD |
