# Supplementary material for: Nuclear Outsourcing of RNA Interference Components to Human Mitochondria
Source: PLoS One. 2011 Jun 13;6(6):e20746. doi: 10.1371/journal.pone.0020746 (PMC3113838; doi:10.1371/journal.pone.0020746)
Supplement: Table S4 — Mitochondrial enrichment in miRNA targets predicted for mitomiRs and control miRNAs. (DOC) [file pone.0020746.s009.doc]

**Supporting information**

**Table S4: Mitochondrial enrichment in miRNA targets predicted for mitomiRs and control miRNAs**

| **MitomiRs** | **Mitochondrial targets** | **Total targets** | **Mitochondrial enrichment (%)** |
| --- | --- | --- | --- |
| hsa-mir-1973 | 4 | 21 | 19.0 |
| hsa-mir-1275 | 47 | 317 | 14.8 |
| hsa-mir-494 | 106 | 779 | 13.6 |
| hsa-mir-513a | 67 | 365 | 18.4 |
| hsa-mir-1246 | 59 | 367 | 16.1 |
| hsa-mir-328 | 25 | 150 | 16.7 |
| hsa-mir-1908 | 6 | 36 | 16.7 |
| hsa-mir-1972 | 37 | 218 | 17.0 |
| hsa-mir-1974 | 16 | 150 | 10.7 |
| hsa-mir-1977 | 17 | 144 | 11.8 |
| hsa-mir-638 | 2 | 6 | 33.3 |
| hsa-mir-1978 | 38 | 246 | 15.4 |
| hsa-mir-1201 | 18 | 104 | 17.3 |
|  |  | MEAN + SD | 17.0+ 3 |
|  |  |  |  |
| **Control miRNAs** | **Mitochondrial targets** | **Total targets** | **Mitochondrial enrichment (%)** |
| hsa-mir-886-3p | 2 | 8 | 25.0 |
| hsa-mir-29a | 44 | 366 | 12.0 |
| hsa-mir-106b | 135 | 817 | 16.5 |
| hsa-mir-107 | 76 | 389 | 19.5 |
| hsa-mir-17 | 143 | 886 | 16.1 |
| hsa-mir-103 | 75 | 387 | 19.4 |
| hsa-mir-191 | 5 | 48 | 10.4 |
| hsa-mir-130a | 83 | 484 | 17.1 |
| hsa-mir-301a | 84 | 495 | 17.0 |
| hsa-mir-20a | 142 | 833 | 17.0 |
| hsa-mir-106a | 148 | 864 | 17.1 |
| hsa-mir-18a | 36 | 213 | 16.9 |
| hsa-mir-31 | 61 | 247 | 24.7 |
|  |  | MEAN + SD | 17.6+2.8 |
