# Supplementary material for: Nuclear Outsourcing of RNA Interference Components to Human Mitochondria
Source: PLoS One. 2011 Jun 13;6(6):e20746. doi: 10.1371/journal.pone.0020746 (PMC3113838; doi:10.1371/journal.pone.0020746)
Supplement: Table S5 — Compilation of target genes and/or genes co-regulated with hsa-miR-328, hsa-mir-494, hsa-mir-513 and hsa-mir-638. (DOC) [file pone.0020746.s010.doc]

**Supporting information**

**Table S5 – Compilation of target genes and/or genes co-regulated with hsa-miR-328, hsa-miR-494, hsa-miR-513 and hsa-miR-638**

| **miRNA ID** | **Gene symbols with positive correlation** | **Gene symbols with negative correlation** |
| --- | --- | --- |
| **Hsa-miR-328** | AAK1, CST3, CTGLF6, DNAJB13, DUSP26, ETS2,  LOC202051, MGLL, MOBKL2B, MPI, MPZ,  MT1H, MUC20, MXI1, MYLK2, MYO15B, MYOCD, NDRG2, NEURL, NFASC, NHSL1, NID1, NKD1, NLGN3, NR0B2, NRCAM, NRP1, NTF3, NXNL1, OBP2A, OCIAD1, OR7E104P, P2RX2, PANX2, PCDHGA10, PDE4C, PDE4DIP, PDGFRA, PDLIM1, PDXDC2, PHC2,  PHLDB1, PKNOX2, PKP2, PLA1A, PLEK2, PLXNA2,  PNCK, RHD, SEMA4G,  SH3D19, TJP2 | ADA, ANKRD30A,  APOBEC3B, ARHGDIB,  ASCC3L1, C11orf82,  C8orf38, CD37, CD69,  CD79A, CD83, CDCA2,  CENPA, CENPE, CENPK,  CENPL, CEP135, CHI3L2,  CXCR4, DBF4, DCK,  DSCC1, DTL, EEF1D,  EIF4A1, ELOVL5, ESPL1,  FAM119A, FCRLA, FLJ12595, GEN1, GLYATL2, hCG_1815491, HIRA, HJURP, HMMR, IGHD, IGHG1, IGHM, IRX5, KIF11, KIF14, KIF15, KIF2C, KNTC1, KRT81, KYNU, LOC201725, LOC286434, LOC647121, MAGOHB, MASTL, MKI67, MND1, MPHOSPH6, MREG,  MS4A1, MYB, MYNN, MYO1G, MYSM1, N4BP3, NCAPG2, NCAPG, NCBP1, NEIL3, NEK2, NFKB2, NHEDC2, NQO1, NTS, NUCKS1, NUDCD1, NUDT1, NUF2, NUP160, NUP54, NUP62, NUPL1, NUSAP1, OBP2B, OIP5, OLFM2, P2RX5, PAICS, PANX1, PBK, PCNA, PEX13, PGD, PHF14, PLEKHG4B, PLK1, PMF1, PMS2L5, POLH, POLQ, POLR3B, POP1, POU2AF1, PPIL5, PRIM2, RAD51, RAD54L, RALGPS2, REL, RFWD3, RHOH, PL10A, RPL23A, RPS11, RPS14, RPS9, SAMSN1, SCGB2A2, SHCBP1, SNHG1, SNHG8, SNX8, TRAF4, TRIM16, UBE2T, WHSC1, YEATS4, ZBTB24, ZNF107, ZNF678, ZYG11A |
| **Hsa-miR-494** | C, IGHD, LOC728448, MMP7, MUC1, MUC20, NAT6, NAT8B, NBR1, ND2, NEK11, NFKBIZ, NMNAT2, NPAS2, NPAS3, NPTXR, NR6A1, NUDT16P, NUDT4P1, OBP2A, ODZ4, OLFM4, OPHN1, OR2A4, PART1, PAX2, PCDH19, PCDHA10, PCDHGA10, PDE6B, PDXDC2, PDZK1IP1, PIGG | FH, MPHOSPH6, RIP, MRPL19, MRPL23, MRPL27, MRPL38, MRPL39, MRPL50,  MRPL52, MRPS11, MRPS15, MSH2, MT1H, NAE1, NAP1L4, NDUFA11, NDUFA6, NDUFB6,  NDUFB8, NDUFS2, NDUFS3, NDUFS4, NDUFV2, NPM3, NQO2, NT5C, NUDT21, PAIP2, PANK1, PCMT1, PDXP,  PFTK1, PIAS4 |
| **Hsa-miR-513** | FAM153B, IGHD, MLLT4,  MRPL20, MSLN, MUC1,  MUC4, MYCNOS, NAT8B,  NDUFA10, NPAS2, NPAS3,  NUDT4P1, ODZ4, OR2A4,  OR2F2, PABPC1, PARD6B,  PCDH19, PCDHGA11, PCDHGC4, PDCL3, PNOC,  POLE, RPL10A, RPL23A,  RPS14, RPS3A, RPS7, SSX2 | MLX, MRPL41, MT1M, NAPA, NAT1, NDUFA11, NDUFA6, NDUFAF1, NDUFS4, NDUFV2, NT5C, ORMDL3, P117, AKAP2, PCGF5, PDXK, PIM3, PIP, PMF1, PMP22, PNPO, POLR2J, RNH1, SDHALP1, SGK3, TBC1D10A, VCP, VKORC1, WIBG |
| **Hsa-miR-638** | IGHG1, MMP7, MRLC2, MSLN, MUC1, MUC20, NPAS2, NPTXR, NR6A1, OXTR, PABPC1, PALM3, PART1, PAX2, PCDHA10, PCDHGA10, PDCL3, PELI2,  PIGG, PLAGL2, PML,  PNOC, RPL36A, RPS14, RPS3A, RPS7, TNFRSF10C | MRPL23, MT1H, NDUFA11,  NDUFS1, NDUFS4, PIP,  POLR2J, SHB |
