# Supplementary material for: Nuclear Outsourcing of RNA Interference Components to Human Mitochondria
Source: PLoS One. 2011 Jun 13;6(6):e20746. doi: 10.1371/journal.pone.0020746 (PMC3113838; doi:10.1371/journal.pone.0020746)
Supplement: Table S6 — Number of miRNA target sites on the mitochondrial genome predicted for mitomiRs and control miRNAs. (DOC) [file pone.0020746.s011.doc]

**Supporting information**

**Table S6: Number of miRNA target sites on the mitochondrial genome predicted for mitomiRs and control miRNAs**

|  | **Computational tools** | | | |
| --- | --- | --- | --- | --- |
|  | **RNA22** | **RegRNA** | **MiRWalk** | **Target Scan** |
| **mitomiRs** | | | | |
| hsa-miR-1973 | 0 | 0 | 0 | 0 |
| hsa-miR-1275 | 11 | 3 | 1 | 3 |
| hsa-miR-494 | 0 | 0 | 0 | 0 |
| hsa-miR-513a-5p | 0 | 0 | 0 | 0 |
| hsa-miR-1246 | 2 | 0 | 1 | 2 |
| hsa-miR-328-5p | 1 | 0 | 0 | 0 |
| hsa-miR-1908 | 5 | 11 | 1 | 4 |
| hsa-miR-1972 | 4 | 1 | 0 | 1 |
| hsa-miR-1977 | 7 | 1 | 0 | 5 |
| hsa-miR-638 | 22 | 1 | 1 | 1 |
| hsa-miR-1974 | 10 | 2 | 0 | 5 |
| hsa-miR-1978 | 4 | 0 | 0 | 7 |
| hsa-miR-1201 | 3 | 0 | 0 | 0 |
| Total number of target sites = 120 | | | | |
| **control miRNAs** | | | | |
| hsa-mir-886-3p | n.a. | 0 | 0 | n.a. |
| hsa-mir-29a | 0 | 0 | 0 | 0 |
| hsa-mir-106b | 1 | 0 | 1 | 1 |
| hsa-mir-107 | 1 | 0 | 0 | 0 |
| hsa-mir-17 | 0 | 0 | 1 | 1 |
| hsa-mir-103 | 1 | 0 | 0 | 0 |
| hsa-mir-191 | 0 | 0 | 0 | 0 |
| hsa-mir-130a | 2 | 0 | 1 | 1 |
| hsa-mir-301a | 1 | 0 | 1 | 1 |
| hsa-mir-20a | 1 | 0 | 1 | 1 |
| hsa-mir-106a | 1 | 0 | 0 | 1 |
| hsa-mir-18a | 3 | 0 | 0 | 0 |
| hsa-mir-31 | 0 | 0 | 0 | 1 |
| Total number of target sites = 20 | | | | |

n.a. indicates that prediction was not available.

For those miRNAs, which had at least one target, we compared the number of hits on mitochondrial genes of mitomiRs versus cytosolic miRNAs using the Mann Whitney test independently for each computational tool. P-values were significative with RNA22 (p=0.003) and TargetScan (p=0.007). Calculation was not applicable with RegRNA and miRWalk.
