# Supplementary material for: Nuclear Outsourcing of RNA Interference Components to Human Mitochondria
Source: PLoS One. 2011 Jun 13;6(6):e20746. doi: 10.1371/journal.pone.0020746 (PMC3113838; doi:10.1371/journal.pone.0020746)
Supplement: Table S7 — Lengths and thermodynamic features of mitomiRs. (DOC) [file pone.0020746.s012.doc]

**Supporting information**

**Table S7: Lengths and thermodynamic features of mitomiRs**

|  | **Length of mature miRNA** | **Length of**  **pre-miRNA** | **MFE**  **(kcal/mol)** | **AMFE**  **(kcal/mol)** | **MFEI** |
| --- | --- | --- | --- | --- | --- |
| **mitomiRs** | | | | | |
| hsa-miR-1973 | 19 | 44 | -9,6 | 21,82 | 0,44 |
| hsa-miR-1275 | 17 | 80 | -26,43 | 33,04 | 0,64 |
| hsa-miR-494 | 22 | 81 | -34,1 | 42,1 | 1,1 |
| hsa-miR-513a | 18 | 129 | -62,9 | 48,76 | 1,1 |
| hsa-miR-1246 | 19 | 73 | -18,81 | 25,77 | 0,59 |
| hsa-miR-328 | 22 | 75 | -45,91 | 61,21 | 0,9 |
| hsa-miR-1908 | 21 | 80 | -45,2 | 56,5 | 0,71 |
| hsa-miR-1972 | 22 | 77 | -38,12 | 49,51 | 1,06 |
| hsa-miR-1974 | 23 | 70 | -18,4 | 26,26 | 0,68 |
| hsa-miR-1977 | 22 | 79 | -20,5 | 25,95 | 0,68 |
| hsa-miR-638 | 25 | 100 | -54,7 | 54,7 | 0,68 |
| hsa-miR-1978 | 21 | 53 | -13,7 | 25,85 | 0,57 |
| hsa-miR-1201 | 24 | 85 | -20 | 23,53 | 0,59 |
| **Control miRNAs** | | | | | |
| hsa-mir-886-3p | 21 | 121 | -35,1 | 29 | 0,53 |
| hsa-mir-29a | 22 | 64 | -24,9 | 38,91 | 1,13 |
| hsa-mir-106b | 21 | 82 | -43,9 | 53,54 | 0,88 |
| hsa-mir-107 | 23 | 81 | -29,6 | 36,54 | 0,78 |
| hsa-mir-17 | 23 | 84 | -34,3 | 40,83 | 0,95 |
| hsa-mir-103-2 | 23 | 78 | -28,1 | 36,03 | 0,8 |
| hsa-mir-191 | 23 | 92 | -49 | 53,26 | 0,89 |
| hsa-mir-130a | 22 | 89 | -42,6 | 47,87 | 0,95 |
| hsa-mir-301a | 23 | 86 | -32,8 | 38,14 | 0,94 |
| hsa-mir-20a | 23 | 71 | -31 | 43,66 | 1,15 |
| hsa-mir-106a | 23 | 81 | -34,7 | 42,84 | 1,02 |
| hsa-mir-18a | 23 | 71 | -22 | 30,99 | 0,69 |
| hsa-mir-31 | 21 | 71 | -36 | 50,7 | 1 |

MFE indicates minimal folding free energy

AMFE indicates adjusted minimal folding free energy

MFEI indicates minimal folding free energy index
