# Supplementary material for: Nuclear Outsourcing of RNA Interference Components to Human Mitochondria
Source: PLoS One. 2011 Jun 13;6(6):e20746. doi: 10.1371/journal.pone.0020746 (PMC3113838; doi:10.1371/journal.pone.0020746)
Supplement: Table S8 — Comparison of MFE, AMFE, MFEI of mitomiRs and control miRNAs. (DOC) [file pone.0020746.s013.doc]

**Supporting information**

**Table S8 : Comparison of MFE, AMFE, MFEI of mitomiRs and control miRNAs**

|  | **mitomiRs** | | | **Control miRNAs** | | |
| --- | --- | --- | --- | --- | --- | --- |
|  | **MFE** | **AMFE** | **MFEI** | **MFE** | **AMFE** | **MFEI** |
| **Mean** | -31,41 | 38,08 | 0,75 | -34,15 | 41,72 | 0,9 |
| **Variance** | 281,51 | 208,40 | 0,05 | 57,82 | 62,83 | 0,03 |

MFE indicates minimal folding free energy

AMFE indicates adjusted minimal folding free energy

MFEI indicates minimal folding free energy index

The units for MFE and AMFE is kcal/mol.
